# Aspirin and Preterm Birth Among Pregnant People With Increased Heat Exposure: Secondary Analysis of a Randomized Clinical Trial

**DOI:** 10.1001/jamanetworkopen.2026.11402

**Published:** 2026-05-06

**Authors:** Gabriella Y. Meltzer, Luke P. Duttweiler, Sarah Saleem, Kartik Shankar, Manolo Mazariegos, Antoinette Tshefu, Jackie K. Patterson, Elwyn Chomba, Waldemar A. Carlo, Shivaprasad Goudar, Richard Derman, Archana B. Patel, Patricia L. Hibberd, Edward Liechty, Fabian Essamai, Edwin Asturias, Denise C. Babineau, Janet Moore, Cascade Tuholske, Andrew Zimmer, Brent A. Coull, Elizabeth McClure, Robert L. Goldenberg, Nancy Krebs, Matthew K. Hoffman, Blair J. Wylie

**Affiliations:** 1 Department of Environmental Science, American University, Washington, DC; 2 Department of Health Studies, American University, Washington, DC; 3 Department of Biostatistics, Harvard T. H. Chan School of Public Health, Boston, Massachusetts; 4 Department of Community Health Sciences, The Aga Khan University Medical College, Karachi, Pakistan; 5 Department of Pediatrics, University of Colorado Anschutz Medical Campus, Aurora; 6 Instituto de Nutrición de Centro América y Panamá, Guatemala City, Guatemala; 7 Kinshasa School of Public Health, Kinshasa, Democratic Republic of the Congo; 8 Department of Pediatrics, University of North Carolina School of Medicine, Chapel Hill; 9 Levy Mwanawasa Medical University, Lusaka, Zambia; 10 Department of Pediatrics, Heersink School of Medicine, University of Alabama at Birmingham; 11 Jawarhalal Nehru Medical College, Belagavi, Karnataka, India; 12 Thomas Jefferson University, Philadelphia, Pennsylvania; 13 Lata Medical Research Foundation, Nagpur, India; 14 Datte Meghe Institute of Higher Education and Research, Wardha, India; 15 Department of Global Health, Boston University School of Public Health, Boston, Massachusetts; 16 Department of Pediatrics, Indiana School of Medicine, Indiana University, Indianapolis; 17 Department of Child Health and Pediatrics, Moi University School of Medicine, Eldoret, Kenya; 18 RTI International, Durham, North Carolina; 19 Department of Earth Sciences, Montana State University, Bozeman; 20 Geospatial Core Facility, Montana State University, Bozeman; 21 Department of Obstetrics and Gynecology, Columbia University Irving Medical Center, New York, New York; 22 Department of Obstetrics and Gynecology, Christiana Care Health System, Newark, Delaware; 23 Department of Obstetrics and Gynecology, Beth Israel Deaconess Medical Center, Brookline, Massachusetts

## Abstract

**Importance:**

Scalable interventions are urgently needed to mitigate the adverse effects of heat on pregnancy and newborn health.

**Objective:**

To evaluate whether low-dose aspirin modifies the association between heat exposure and preterm birth.

**Design, Setting, and Participants:**

This secondary analysis of the Global Network for Women’s and Children’s Health Research Aspirin Supplementation for Pregnancy Indicated Risk Reduction in Nulliparas (ASPIRIN) randomized, double-blinded, placebo-controlled clinical trial was conducted from March 2016 to June 2018. Statistical analyses were performed from June 2024 to June 2025. The study settings included the Democratic Republic of Congo, Zambia, Kenya, Guatemala, Pakistan, and Belagavi and Nagpur, India. Participants included nulliparous individuals between 6 and 13 weeks’ gestation recruited through local clinics and communities, with delivery at 20 or more weeks’ gestation.

**Exposures:**

Prenatal care site–specific daily maximum humid heat averaged across gestation and by gestational week, and randomization to aspirin or placebo.

**Main Outcome and Measure:**

The main outcome was preterm birth (delivery between 20 and <37 weeks’ gestation) with gestational age confirmed by enrollment ultrasonography.

**Results:**

Of 11 558 participants (mean [SD] age, 20.9 [3.3] years), 5787 were randomized to receive aspirin and 5771 to receive placebo. Preterm birth occurred among 754 placebo recipients (13.1%) and 668 aspirin recipients (11.6%). In mixed-effects pooled logistic regression, each 1 °C increase in mean daily maximum shaded wet-bulb globe temperature across gestation was associated with a 5% increased odds of preterm birth (adjusted odds ratio, 1.05; 95% CI, 1.01-1.10). In stratified analyses, this increased risk was observed only among placebo recipients (adjusted odds ratio [AOR], 1.07; 95% CI, 1.02-1.13), not among aspirin recipients (AOR, 1.03; 95% CI, 0.97-1.10). In pooled mixed-effects logistic distributed lag models, increased odds of preterm birth were observed 17 to 19 weeks before delivery among individuals whose daily maximum shaded wet-bulb globe temperature exceeded the site-specific 75th percentile compared with the lowest 3 quartiles. This vulnerability was not observed among aspirin recipients. In contrast, the association of heat with perinatal mortality was observed only among those receiving aspirin (AOR, 1.15; 95% CI, 1.05-1.26) and not among those receiving placebo (AOR, 1.03; 95% CI, 0.96-1.11).

**Conclusions and Relevance:**

The findings of this secondary analysis of the Global Network ASPIRIN trial suggest that low-dose aspirin initiated early in pregnancy among nulliparous individuals may mitigate the effects of heat exposure on preterm birth. The increasing global prevalence of heat stress warrants testing its efficacy more broadly among pregnant people as well as its safety with respect to perinatal mortality.

**Trial Registration:**

ClinicalTrials.gov Identifier: NCT02409680

## Key Points

**Question:**

Does low-dose aspirin modify humid heat’s effect on preterm birth incidence?

**Findings:**

In this secondary analysis of a randomized clinical trial, a greater mean daily maximum humid heat across gestation and in the 17 to 19 weeks predelivery was associated with increased odds of preterm birth among nulliparous individuals randomized to placebo, but not among those randomized to aspirin. However, heat was associated with perinatal mortality only among recipients of aspirin.

**Meaning:**

These findings suggest that low-dose aspirin initiated early in pregnancy may mitigate the effects of heat exposure on preterm birth in nulliparous individuals, and increasing prevalence of heat stress warrants testing its broader efficacy in pregnant people and its safety regarding perinatal mortality.

## Teaser

This secondary analysis of randomized clinical trial evaluates whether low-dose aspirin modifies the association between heat exposure and preterm birth.

## Introduction

Extreme heat events caused by climate change are intensifying in frequency and severity, with the 10 warmest years on record all occurring in the past decade.^1^ There is mounting evidence that extreme heat exposure is associated with adverse maternal and newborn health outcomes, particularly preterm birth (PTB) and low birth weight.^2^ These effects may be even more pronounced in low- and middle-income countries (LMICs) that face disproportionate effects of climate change and worse perinatal outcomes.^3^ Feasible, low-cost, and effective scalable interventions are needed to mitigate heat-related adverse pregnancy and newborn health outcomes.

Research has confirmed the association between extreme heat and PTB across diverse geographies. In a 2020 systematic review and meta-analysis, Chersich et al^4^ found that the mean odds of PTB increased by 5% for each 1 °C increase in mean temperature throughout gestation and increased by 16% on heatwave days compared with nonheatwave days. Using demographic and health survey data from 14 LMICs, McElroy and colleagues^5^ found that exposure to higher maximum temperatures and smaller diurnal temperature ranges in the week preceding birth significantly increased the risk of PTB. Although mechanisms linking heat stress to adverse pregnancy outcomes remain incompletely understood, inflammation appears to play a role. Heat stress during pregnancy can lead to reduced splanchnic and placental blood flow and an increased release of heat shock proteins, both of which can induce inflammatory responses.^6^

Roughly 12 to 15 million births annually are preterm, and 1 million of these infants die from complications of prematurity. PTB rates globally range from 9.3% to 12.6%, with 90% occurring in LMICs.^7^ Despite multinational investments in global maternal and child health, there has been no meaningful decrease in PTB rates in recent years.^8^ Progress has also largely been eroded by the effects of climate change in LMICs with fragile, vulnerable health systems.^9^ Even slight temperature-related increases in PTB risk associated with climate change will result in substantial maternal and child morbidity and mortality, especially in LMICs.

The Global Network (GN) for Women’s and Children’s Health Research is a network of US and international investigators in sub-Saharan Africa, South Asia, and Latin America that conducts studies testing feasible and sustainable interventions to improve maternal and newborn health.^10^ From March 2016 to June 2018, the GN for Women’s and Children’s Health Research conducted the Aspirin Supplementation for Pregnancy Indicated Risk Reduction in Nulliparas (ASPIRIN) trial, a randomized, double-blinded, placebo-controlled trial across 7 international sites evaluating whether low-dose aspirin initiated during the first trimester reduced the risk of PTB and other adverse outcomes. The study demonstrated that participants randomized to aspirin were significantly less likely to experience PTB (relative risk [RR] 0.89; 95% CI, 0.81-0.98) and/or perinatal mortality (RR, 0.86; 95% CI, 0.73-1.00).^11^

This secondary analysis used data from the GN ASPIRIN trial to evaluate low-dose aspirin as a potential intervention to mitigate extreme heat exposure’s adverse effects on the incidence of PTB. Specifically, the study aimed to answer (1) whether acute and/or long-term prenatal ambient heat exposure was associated with PTB or other adverse pregnancy outcomes and (2) whether the administration of low-dose aspirin modified these heat-related effects.

## Methods

### Study Sample

This secondary analysis of a randomized clinical trial used data from the now-completed ASPIRIN trial (trial protocol and statistical analysis plan in Supplement 1).^11,12^ In brief, trial participants were recruited using clinic- and community-based methods from 105 enrollment clusters where participants received antenatal care in proximity to their homes, located within 7 GN study sites: the Democratic Republic of Congo, Zambia, Kenya, Guatemala, Pakistan, and Nagpur and Belagavi, India. Eligible participants were nulliparous and aged 14 to 40 years, with a singleton pregnancy between 6 weeks’ and 0 days’ and 13 weeks’ and 7 days’ gestation confirmed by ultrasonography. Enrolled participants were randomly assigned to either daily administration of 81 mg of acetylsalicylic acid (aspirin) or an identical placebo from the time of enrollment through week 36. In addition to monitoring clinical outcomes, participants were queried about sociodemographics, medical history, and prenatal care. All participants provided written informed consent. Procedures were approved by the ethics review committees of all involved international sites and US partner institutions (Belgaum, India: JN Medical College, Belagavi, India and Thomas Jefferson University, Philadelphia, PA; Democratic Republic of Congo: Kinshasa School of Public Health, Kinshasa, Democratic Republic of Congo and University of North Carolina School of Medicine; Guatemala: Instituto de Nutrición de Centroamérica y Panamá and University of Colorado Health Care System; Kenya: Moi University School of Medicine and Indiana University School of Medicine; Nagpur, India: Lata Medical Research Foundation, Nagpur, India and Boston University School of Public Health; Pakistan: The Aga Khan University Medical College, Karachi Pakistan and Columbia University; Zambia: University Teaching Hospital, Lusaka, Zambia; University of Alabama at Birmingham; and RTI International, Research Triangle Park, NC). This secondary analysis was not prespecified and is reported in accordance with the Strengthening the Reporting of Observational Studies in Epidemiology (STROBE) reporting guideline.

### Exposure

Our primary exposure of interest was ambient heat, measured using shaded wet-bulb globe temperature (WBGT). WBGT is an internationally used metric for heat stress that is a weighted mean of the ambient, wet-bulb, and globe temperatures that incorporates thermal, solar, and convective heat transfers from ambient temperature, humidity, solar radiation, and wind speed, conceptualized more easily as the “felt” temperature.^13^ WBGT is frequently used in epidemiologic studies evaluating extreme heat during pregnancy.^14,15,16,17,18^

We calculated shaded WBGT from ECMWF Reanalysis version 5 (ERA5) climate reanalysis.^19^ Available hourly since 1940 at 0.25° × 0.25° resolution (approximately 31 km), ERA5 reanalysis is widely used in heat-epidemiological research in LMICs.^16,20,21^ We used the hourly 2-m air temperature, the dew point temperature, and surface pressure data to estimate the hourly relative humidity.^22,23^ We then estimated the hourly heat index from the hourly 2-m air temperature and relative humidity following the US National Weather Service procedure,^24^ converting to hourly shaded WBGT using a quadratic transformation.^25^ This estimation of shaded WBGT has been shown to be accurate when compared with station data across a range of climates.^26^

We chose to use the maximum daily WBGT rather than the mean or minimum WBGT because (1) pregnant individuals in GN settings often engage in outdoor labor and are exposed to high daytime temperatures and related physiological stress, and (2) we aimed to evaluate aspirin as a tool to protect against exposure to increasing extreme heat.^27^ Within each site, we geocoded participants to their antenatal care enrollment cluster, as people seek care at facilities accessible to their homes. For long-term heat exposure, we averaged each participant’s daily maximum daily WBGT exposure across gestation, beginning at the ultrasonography-derived conception date until delivery. For acute heat exposure, we averaged the maximum daily WBGT across each gestational week until delivery, investigated the percentile of heat exposure for that gestational week relative to all other participants in the same GN country site, and compared those exposed to acute heat events (>75th percentile vs <75th centile). Site-specific centiles were constructed to avoid heat being a proxy for GN country location, as there was wide variation in mean temperatures across our sites.

### Outcomes

Our primary outcome of interest was PTB, defined as a stillbirth or live birth between 20 weeks and 0 days and 36 weeks and 6 days, which we modeled against both long-term and acute heat exposure. We also examined several secondary outcomes against long-term heat exposure only, including hypertensive disorders of pregnancy (HDPs), small for gestational age (SGA), and perinatal mortality. HDPs were based on the participant developing blood pressure higher than 140/90 mm Hg after 20 weeks’ gestation, with either elevated systolic or diastolic blood pressure measured on 2 occasions or diagnosed at delivery.^28^ SGA was defined as a sex- and gestational age–specific birth weight less than the 10th percentile using INTERGROWTH-21st (International Fetal and Newborn Growth Consortium for the 21st Century) standards, measured within 4 days of a live birth.^29^ Perinatal mortality was defined in the trial as a stillbirth or neonatal death before 7 days after delivery. Our effect modifier of interest was treatment group (low-dose aspirin or placebo randomization).

### Statistical Analysis

We limited analyses to the trial’s modified intent-to-treat population, which included eligible participants who were randomized and delivered after 20 weeks. We used mixed-effects pooled logistic regression to evaluate the association between long-term heat exposure—mean maximum daily WBGT across gestation—and PTB.^30^ We treated PTB as a time-to-event outcome, allowing each participant to contribute WBGT exposure data for all gestational weeks until delivery, with birth as a binary outcome for each gestational week. This approach pooled multiple data frames for each gestational week into a single model, estimating the association between long-term heat exposure and PTB. We treated HDPs and SGA as binary outcomes and used mixed-effects logistic regression to estimate their associations with long-term heat exposure. We considered several covariates, including maternal age, education, body mass index, gravidity, infant sex, adequate antenatal care visits (≥4), delivery location, delivery mode, gestational age, and study site. Using a directed acyclic graph, we retained maternal age, gestational age at delivery, infant sex, and study site in the final models.^31^ Gestational age was included as a covariate when treating PTB as a time-to-event outcome, as the probability of delivery necessarily increases with gestational age. We included study site as a random intercept to avoid the mean maximum daily WBGT being a proxy for a given GN site. To evaluate aspirin’s potential effect modification on the association between long-term heat exposure and each outcome, we first stratified each model by treatment group and evaluated effect estimates by strata. We then included an interaction term between the mean maximum daily WBGT and treatment group and tested its statistical significance. *P* values were 2 sided, and *P* < 0.05 was the threshold prespecified for statistical significance.

To evaluate the acute association of heat exposure with PTB, we combined the mixed-effects pooled logistic framework with distributed lag models (DLMs) to identify time-varying estimates of the lagged association between the mean gestational week–specific maximum daily WBGT and the odds of PTB.^32^ Whereas mixed-effects pooled logistic regression can model the overall risk of time to a given event, incorporating the DLM framework allowed us to identify critical windows of gestation during which acute elevated heat exposure would increase the risk of PTB. We set our lag to 20 weeks before delivery as inclusion in our analytic sample required delivery at 20 or more weeks, and the DLM approach requires a square exposure data structure with equal contribution from all individuals. The lagged weeks did not correspond to a specific gestational week, but rather weeks “lagged” before delivery, whether full term or preterm. This nonetheless enabled us to examine whether acute extreme heat would trigger PTB (manifested as risk highest in the weeks immediately preceding delivery), as well as whether acute heat exposure would initiate a cascade of events leading up to later PTB (manifested as risk highest the farther the lag from delivery). For each lagged gestational week, the model estimated the odds of PTB for participants whose mean maximum daily WBGT exceeded their GN site-specific 75th percentile compared with those below this threshold. Models were adjusted for maternal age, gestational age, and infant sex, with study site as a random intercept. We stratified the models by treatment group (aspirin vs placebo) and compared any identified weeks of increased susceptibility to heat for PTB. We conducted all analyses in R, version 4.4.3 (R Project for Statistical Computing) from June 1, 2024, to June 29, 2025, and fit DLMs using the R package dlnm.^33^

## Results

### Study Participants, Heat Exposure, and Clinical Outcomes

**Figure 1.**
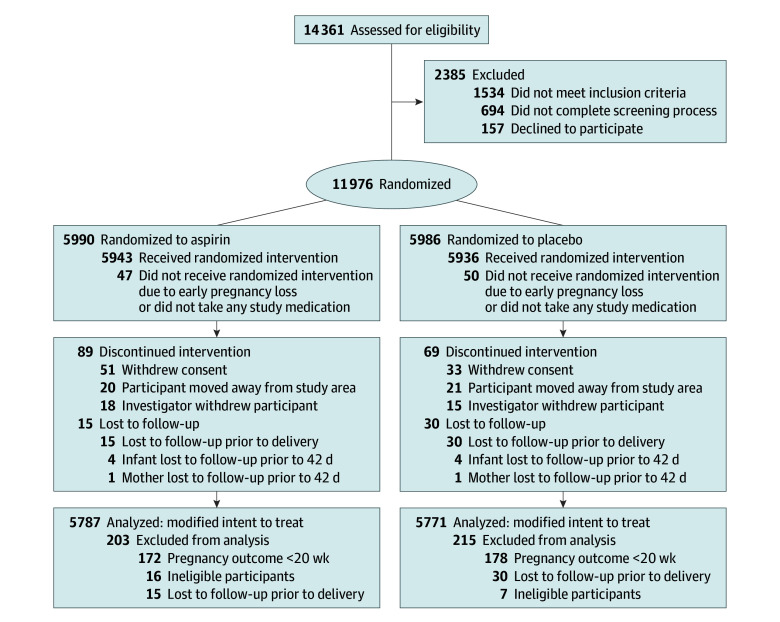
Flow Diagram of Randomization and Follow-Up in the Aspirin Supplementation for Pregnancy Indicated Risk Reduction in Nulliparas Trial

Our analytic sample included 11 558 participants (49.9%; mean [SD] age, 20.9 [3.3] years), 5787 (50.1%) randomized to aspirin and 5771 randomized to a placebo (Figure 1).^11^ The age breakdown among the participants was as follows: 6801 (58.8%) were 20 to 29 years, 4506 (39.0%) were younger than 20 years, and only 126 (2.2%) were older than 29 years. A slight majority were recruited after 10 weeks’ gestation (6188 [53.5%]). Most participants were in India (2650 [22.9%] in Belagavi and 2046 [17.7%] in Nagpur), followed by Guatemala (1671 [14.5%]), Pakistan (1533 [13.3%]), Kenya (1328 [11.5%]), the Democratic Republic of Congo (1320 [11.4%]), and Zambia (1010 [8.7%]). Sociodemographic characteristics are shown in Table 1.

**Table 1.** Heat Exposure, Baseline Characteristics, and Clinical Outcomes Among ASPIRIN Study Participants by Treatment Arm (N = 11 558)^a^

Characteristic	Participants, No. (%)			*P* value^b^
	Full cohort (n = 11 558)	Aspirin (n = 5787)	Placebo (n = 5771)	
Heat exposure				
Mean (SD) daily maximum WBGT across gestation	23.8 (3.1)	23.8 (3.1)	23.8 (3.1)	NA
Mean (SD) daily maximum WBGT in trimester 1	23.7 (3.5)	23.7 (3.5)	23.8 (3.5)	NA
Mean (SD) daily maximum WBGT in trimester 2	23.9 (3.6)	23.9 (3.6)	23.9 (3.6)	NA
Mean (SD) daily maximum WBGT in trimester 3	23.8 (3.5)	23.8 (3.5)	23.8 (3.5)	NA
Baseline characteristics				
Maternal age, y				
<20	4506 (39.0)	2233 (38.6)	2273 (39.4)	NA
20-29	6801 (58.8)	3429 (59.3)	3372 (58.4)	NA
>29	251 (2.2)	125 (2.2)	126 (2.2)	NA
Gravida				
Primiparous	10 500 (90.8)	5274 (91.1)	5226 (90.6)	NA
1 Prior loss	920 (8.0)	451 (7.8)	469 (8.1)	NA
2 Prior losses	138 (1.2)	62 (1.1)	76 (1.3)	NA
Gestational age at enrollment, wk				
<10	5370 (46.5)	2718 (47.0)	2652 (46.0)	NA
≥10	6188 (53.5)	3069 (53.0)	3119 (54.0)	NA
Infant sex^c^				
Male	5943 (51.6)	2982 (51.7)	2961 (51.5)	NA
Female	5580 (48.4)	2789 (48.3)	2791 (48.5)	NA
Maternal education, years of schooling^c^				
None	1658 (14.3)	828 (14.4)	830 (14.3)	NA
<7	1713 (14.8)	856 (14.8)	857 (14.8)	NA
≥7	8185 (70.8)	4086 (70.8)	4099 (70.8)	NA
Maternal BMI categories				
Underweight (<18.5)	2986 (25.9)	1488 (25.7)	1498 (26.0)	NA
Normal (18.5-24.9)	7039 (60.9)	3509 (60.7)	3530 (61.2)	NA
Overweight or obese (≥25)	1526 (13.2)	784 (13.6)	742 (12.9)	NA
Antenatal care visits (≥4)	9213 (79.7)	4600 (79.5)	4613 (79.9)	NA
Delivery location^c^				
Hospital	6973 (60.4)	3479 (60.1)	3494 (60.6)	NA
Clinic or health center	3583 (31.0)	1818 (31.4)	1765 (30.6)	NA
Home or other	997 (8.6)	488 (8.4)	509 (8.8)	NA
Method of delivery^c^				
Vaginal	8581 (74.3)	4257 (73.7)	4324 (75.0)	NA
Cesarean	2963 (25.7)	1523 (26.3)	1440 (25.0)	NA
Site				
Democratic Republic of Congo (14 clusters)	1320 (11.4)	655 (11.3)	665 (11.5)	NA
Zambia (11 clusters)	1010 (8.7)	499 (8.6)	511 (8.9)	NA
Guatemala (18 clusters)	1671 (14.5)	836 (14.5)	835 (14.5)	NA
Belagavi, India (16 clusters)	2650 (22.9)	1327 (22.9)	1323 (22.9)	NA
Pakistan (14 clusters)	1533 (13.3)	771 (13.3)	762 (13.2)	NA
Nagpur, India (20 clusters)	2046 (17.7)	1026 (17.7)	1020 (17.7)	NA
Kenya (12 clusters)	1328 (11.5)	673 (11.6)	655 (11.4)	NA
Clinical outcomes				
Preterm delivery <37 wk^c^	1422 (12.3)	668 (11.6)	754 (13.1)	.01
Small for gestational age^c^	3070 (28.0)	1506 (27.4)	1564 (28.6)	.17
Hypertensive disorders of pregnancy^c^	677 (5.9)	352 (6.1)	325 (5.6)	.32
Stillbirth^c^	307 (2.7)	141 (2.4)	166 (2.9)	.16
Birth weight measured within 4 d of delivery, mean (SD), g	2787 (511)	2790 (493)	2786 (529)	.71
Maternal mortality^c^	20 (0.2)	8 (0.1)	12 (0.2)	.50
Perinatal mortality^c^	327 (2.9)	153 (2.7)	174 (3.1)	.23

Abbreviations: ASPIRIN, Aspirin Supplementation for Pregnancy Indicated Risk Reduction in Nulliparas; BMI, body mass index (calculated as weight in kilograms divided by height in meters squared); NA, not applicable; WBGT, wet-bulb globe temperature.

^a^ Modified intent-to-treat population based on having been randomized to the ASPIRIN trial, eligible, and delivered at 20 weeks or more.

^b^ Determined by use of the χ^2^ test.

^c^ Numbers do not add up due to missing values.

**Figure 2.**
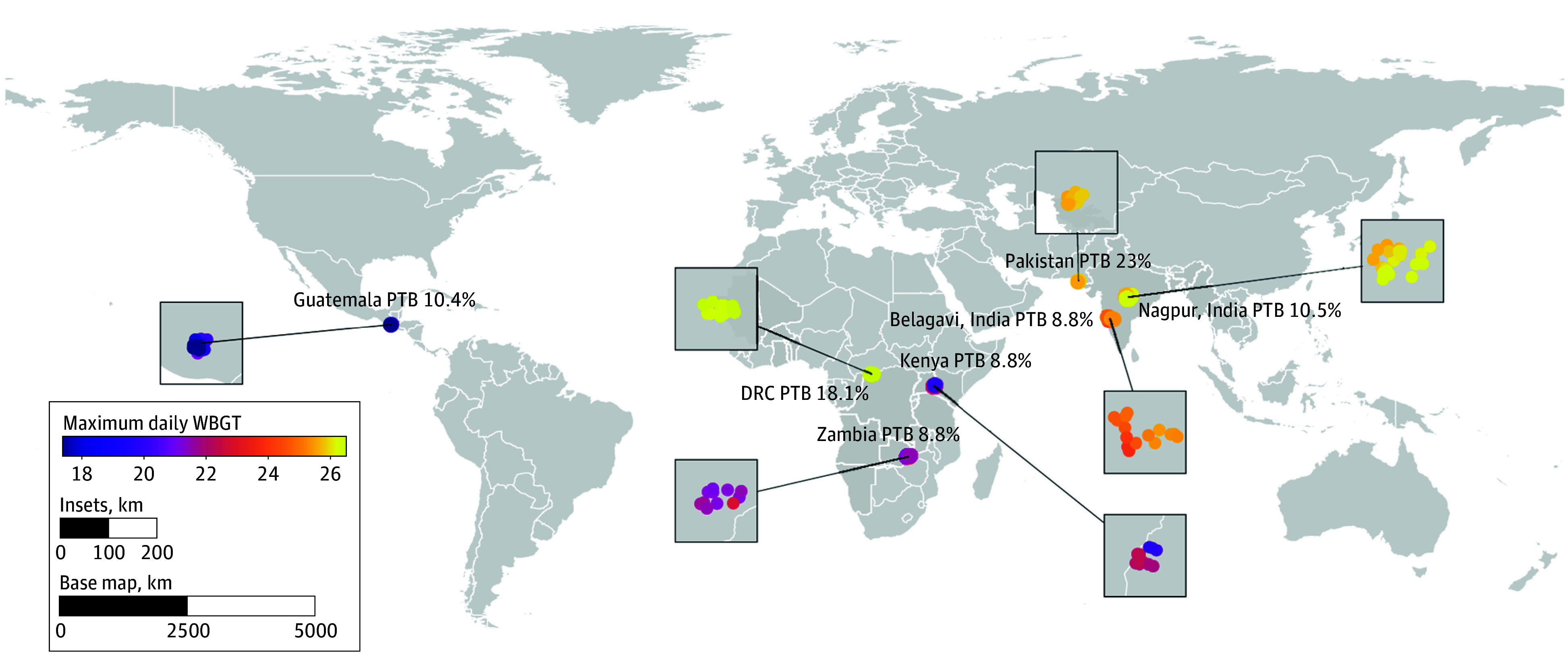
Map Showing Geographic Distribution of Study Sites Showing Mean Maximum Daily Wet-Bulb Globe Temperature (WBGT) and Preterm Birth (PTB) Rates DRC indicates Democratic Republic of Congo.

The mean maximum daily WBGT differed across study sites (range, 18.4 °C to 26.5 °C) (Figure 2; eTable 1 in Supplement 2). The mean (SD) maximum daily WBGT across gestation in both treatment groups was 23.8 °C (3.1 °C). In total, 1422 of 11 558 participants (12.3%) in the overall cohort gave birth preterm, and PTB rates differed across sites, with the highest in Pakistan (353 of 1533; 23.0%) and the lowest in Belagavi (234 of 2646; 8.8%), Zambia (89 of 1009; 8.8%), and Kenya (117 of 1327; 8.8%) (Figure 2). As shown in the original ASPIRIN trial, 673 (11.6%) of aspirin recipients gave birth preterm compared with 754 (13.1%) randomized to a placebo (*P* = .01).^11^ None of the other considered adverse pregnancy outcomes differed by treatment group (Table 1).

### Long-Term Heat Exposure

Each 1 °C increase in the mean maximum daily WBGT across pregnancy was associated with 5% greater odds of PTB (adjusted odds ratio [AOR], 1.05; 95% CI, 1.01-1.10) (Table 2). In stratified analyses, the mean maximum daily WBGT across pregnancy was associated with greater odds of PTB among those randomized to a placebo (AOR, 1.07; 95% CI, 1.02-1.13), but this was not observed among those randomized to aspirin (AOR, 1.03; 95% CI, 0.97-1.10) (Table 2). The maximum daily WBGT and treatment assignment interaction were not statistically significant.

**Table 2.** Association Between Daily Maximum Daily WBGT Averaged Across Gestation and Adverse Pregnancy Outcomes

Outcome^a^	Adjusted odds ratio (95% CI)		
	Overall cohort	Aspirin group	Placebo group
Primary outcome			
Preterm birth^b^	1.05 (1.01-1.10)^c^	1.03 (0.97-1.10)	1.07 (1.02-1.13)^d^
Secondary outcomes			
Hypertensive disorders of pregnancy^e^	1.03 (0.95-1.12)	1.04 (0.93-1.16)	1.01 (0.90-1.13)
Small for gestational age^e^	0.99 (0.95-1.04)	1.04 (0.97-1.11)	0.96 (0.90-1.03)
Perinatal mortality^e^	1.08 (1.01-1.16)^c^	1.15 (1.05-1.26)^d^	1.03 (0.96-1.11)

^a^ The effect estimates represent an increase in the risk of the adverse outcome per 1° increase in the mean maximum daily wet-bulb globe temperature (WBGT) across gestation.

^b^ Adjusted odds ratio and 95% CI from pooled mixed-effects logistic regression adjusted for gestational age at birth, maternal age, infant sex, and study site as a random effect.

^c^ *P* < .05.

^d^ *P* < .01.

^e^ Adjusted odds ratio and 95% CI from mixed-effects logistic regression adjusted for maternal age, infant sex, and study site as a random effect.

Among secondary outcomes, HDPs and SGA were not associated with the mean maximum daily WBGT across pregnancy. Perinatal mortality occurred in 327 of 11 558 participants (2.9%) and was associated with a higher mean maximum daily WBGT across gestation (AOR, 1.08; 95% CI, 1.01-1.16) (Table 2). In stratified models, this association was present among those randomized to aspirin (153 of 5787 [2.7%]; AOR, 1.15; 95% CI, 1.05-1.26) but not among those randomized to placebo (174 of 5771 [3.1%]; AOR, 1.03; 95% CI, 0.96-1.11) (Table 2), in contrast to our observations for PTB.

### Acute Heat Exposure

**Figure 3.**
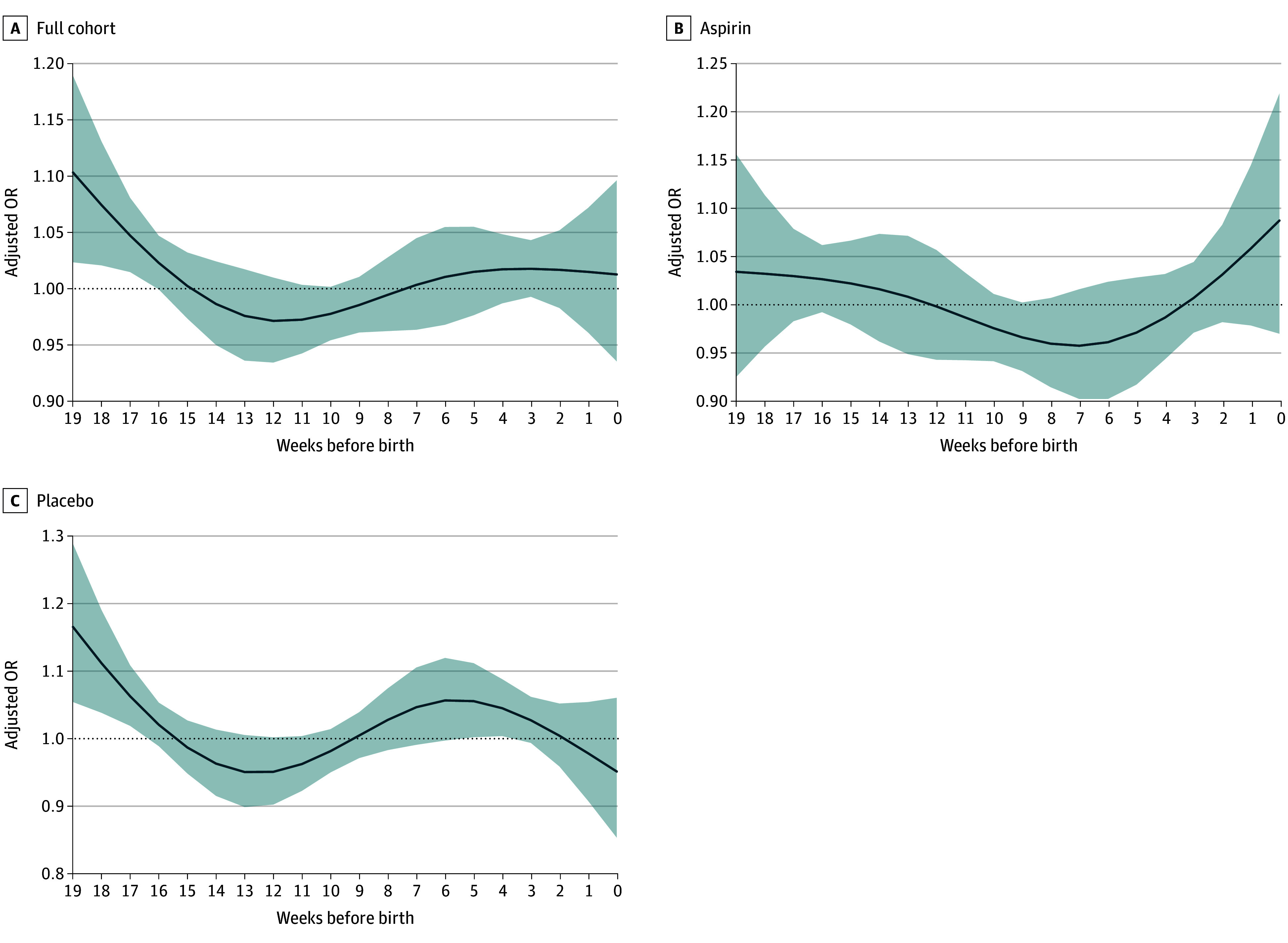
Line Graphs of Association Between Weekly Mean Maximum Daily Wet-Bulb Globe Temperature (WBGT) Exceeding Site-Specific 75th Percentile and Preterm Birth Models were adjusted for maternal age, infant sex, and gestational week, with random effects for site, and show risk of preterm birth based on mean maximum daily WBGT greater than 75th percentile. The curves indicate weekly adjusted odds ratios (ORs), and the shaded areas indicate 95% CIs. Week 0 on the right-hand side of the x-axis indicates time of delivery.

Pooled mixed-effects logistic DLMs showed that in the full cohort, there were significantly increased odds for PTB among those whose mean maximum weekly WBGT was greater than their site-specific 75th percentile in the 17- to 19-week window before delivery compared with those in the lower 3 quartiles of heat exposure (Figure 3; eTable 2 in Supplement 2). Stratified analyses showed that these associations occurred only among those randomized to placebo (Figure 3).

## Discussion

This secondary analysis of the GN’s ASPIRIN trial examined whether long-term and/or acute heat exposure in LMICs was associated with elevated risk of PTB and whether low-dose aspirin initiated early in pregnancy modified this risk. For long-term heat exposure, we found that a 1 °C increase in the mean maximum daily WBGT across pregnancy was associated with 5% greater odds of PTB, and this association was only observed among those randomized to a placebo. Among aspirin recipients, there were no increased odds of PTB associated with heat exposure. This indicated aspirin’s potential protective effect against PTB, although our study lacked sufficient power to detect a significant effect modification. Among secondary outcomes, perinatal mortality was also associated with a greater maximum daily WBGT averaged across pregnancy in the entire cohort, and, in contrast to PTB results, this association was demonstrable only among those randomized to aspirin. Evidence of the effect of long-term heat exposure on HDPs and SGA was less clear.^34,35,36,37^

In identifying critical gestational windows for PTB risk, we did not observe extreme heat to trigger delivery in the weeks immediately preceding birth. Rather, we observed increased odds of PTB among those whose mean maximum weekly WBGT was greater than or equal to their site-specific 75th percentile in the 17- to 19-week window preceding delivery. Similar to long-term exposure models, this risk was observed only among placebo recipients and not among those randomized to aspirin. These results suggested that extreme heat exposure may have initiated a cascade of events that ultimately led to spontaneous PTB, which could have been mitigated by low-dose aspirin’s anti-inflammatory or antithrombotic effects.^38^ Although DLMs have been used to examine critical gestational windows and the risk of PTB,^39,40,41,42,43,44,45,46,47^ few studies have been conducted in LMICs that bear the greatest burden of both PTB and extreme temperatures and the least access to indoor cooling.^5^ Furthermore, we provided a methodologically novel approach applying DLMs in pooled logistic regression, allowing the DLM framework to be used for a time-to-event outcome.

Our analysis identified low-dose aspirin initiated early in gestation as a possible low-cost, scalable intervention to reduce PTBs associated with heat exposure. Several studies have evaluated community-based interventions to address extreme heat exposure in pregnancy in LMICs, including in Kenya and Burkina Faso.^48,49^ LaPointe and colleagues^50^ found that participation in a cash transfer program in rural northern Ghana negated the association between heat and low birth weight. In Egypt, pregnant people who worked outdoors and were assigned to a clinic-based educational intervention on health-related behaviors associated with heat were less likely to experience anemia, PTB, gestational hypertension, fetal growth restriction, antepartum hemorrhage, or low birth weight.^51^ Administering low-dose aspirin could be a cost-effective, straightforward individual-level strategy to save lives and reduce health care expenditures associated with prenatal heat exposure.^52^

Our analysis is hypothesis generating. Caution is warranted, as aspirin did not mitigate the association between heat and perinatal mortality; instead, we noted an increased effect size among aspirin recipients compared with placebo recipients. Our hypothesis centered around PTB, as this was the primary outcome in the original ASPIRIN trial. It is unclear whether our observations for perinatal mortality reflected the true effect of aspirin or whether the small sample size of perinatal deaths contributed to type I error. In the original study, there was no evidence of increased hemorrhagic adverse events, and perinatal mortality was marginally lower among those randomized to aspirin. Residual confounding by factors such as malaria could have also contributed. In a substudy of approximately 10% of the original ASPIRIN trial, peripheral blood was tested for the presence of malaria parasites via polymerase chain reaction twice during antenatal visits.^53^ Malaria modified the association between aspirin exposure and perinatal mortality. Among pregnancies complicated by maternal malaria parasitemia, aspirin was associated with an increased risk of perinatal mortality compared with placebo (RR, 1.69; 95% CI, 0.91-1.34), whereas among pregnancies without parasitemia, aspirin remained protective (RR, 0.56; 95% CI, 0.31-1.03; *P* = .01 for interaction). As malarial status was obtained only in this subsample, we were unable to adjust for it in our models. Our findings with respect to perinatal mortality underscore the need for research in larger cohorts, especially in malaria-endemic climates.

### Limitations

This study had several limitations. First, it was unclear how well enrollment cluster temperatures reflected study participants’ daily heat exposure, given factors such as distance traveled to receive antenatal care, time spent indoors, and limitations of climate reanalysis data. There may have been exposure misclassification because, despite being widely used in environmental epidemiology, the resolution of grids in ERA5 is 25 km × 25 km. In addition, ERA5 air temperature data are known to have a cold bias,^54,55^ and we may not have measured the effects of truly high air temperatures. We were also limited by the inability to discern the etiologies of PTB and whether indications for PTB differed between aspirin and placebo recipients, as this information was unavailable in the parent trial.

## Conclusions

This secondary analysis of the GN ASPIRIN trial pointed to the potential mitigating effects of low-dose aspirin initiated early in pregnancy on heat exposure-related PTB. Larger cohort studies in LMICs are needed to confirm aspirin’s potential protective effect, including among multiparous individuals, and affirm its safety in malaria-endemic climates.^56,57^ Given climate change’s role in shaping maternal and child health, research on low-dose aspirin’s scalability as a feasible and accepted component of multipronged interventions to prevent population-level risk of PTB due to extreme heat in low-resource settings is warranted.

## Notes

Supplement 1.
Trial Protocol and Statistical Analysis Plan

Supplement 2. **eTable 1.** Mean daily maximum, daily minimum, and daily mean wet bulb globe temperatures (in degrees Celsius) by site of the Global Network for Women’s and Children’s Health Research low-dose ASPIRIN trial, during the study period **Table 2.** Time-varying associations between exposure to averaged maximum wet bulb globe temperature exceeding the site-specific 75th percentile at specific weeks before birth and risk of preterm birth (adjusted odds ratios and 95% confidence intervals)

Supplement 3.
Data Sharing Statement
