# Supplementary material for: Aspirin and Preterm Birth Among Pregnant People With Increased Heat Exposure: Secondary Analysis of a Randomized Clinical Trial
Source: JAMA Netw Open. 2026 May 6;9(5):e2611402. doi: 10.1001/jamanetworkopen.2026.11402 (PMC13150641; doi:10.1001/jamanetworkopen.2026.11402)

Aspirin Supplementation for Pregnancy Indicated Risk Reduction In Nulliparas (ASPIRIN)

ClinicalTrials.gov number:	NCT02409680
Protocol number:	GN-LDA.8.1
Lead Study Investigator(s):	Matthew Kendall Hoffman, MD, MPH-Christiana Care<br>Richard J Derman, MD, MPH-Thomas Jefferson University<br>Bhala S Kodkany, MD-Jawaharlal Nehru Medical College (JNMC)<br>Shivaprasad S Goudar, MD, MHPE- JNMC
Subcommittee Members:	Marion Koso-Thomas, MD-NICHD<br>Menachem Miodovnik, MD-NICHD<br>Dennis Wallace, PHD-RTI International<br>Elizabeth McClure, PHD-RTI International<br>Jennifer J. Hemingway-Foday, MPH-RTI International<br>Robert Silver, MD-University of Utah
Version Number:	Version 1.3
Version Date:	February 21, 2019
Funding:	*Eunice Kennedy Shriver* National Institute of Child Health and Human Development

Version Tracking

Version	Date	Authors	Comments
0.0.1	03 Nov 2014	MH, DW, NG, EMac	Draft reviewed by WG at November 12 planning meeting
0.0.2	17 Dec 2014	JHF	Incorporated WG comments and added more detail to sections on study procedures and data forms in preparation for GN Investigator review
0.1.0	18 Dec 2014	NG, JHF	Minor revisions. This version will be sent to GN investigators for initial review.
0.1.1	10 Jan 2015	JHF in response to PI/SFI review	▪ Made revisions to improve clarity and fix grammar/spelling as suggested by reviewers.<br>▪ Incorporated comments from R. Derman, S. Goudar, E. Liechty, F. Jaeger, C Bose, W. Carlo that need to be discussed further.<br>▪ Updated Sample Size section to consistently note a 20% reduction instead of 25%.<br>▪ Added secondary outcomes from the survey responses.<br>▪ Added comments and information from the survey responses for discussion/consideration by WG.
0.2.0	19 Jan 2015	JHF and MH	▪ Finalized revisions from all reviewers. This version was circulated to GN Investigators in advance of the Jan SC meeting.
0.2.1	29 Jan 2015	JHF and MH	▪ Revisions based on investigator feedback at SC Meeting
0.2.2	06 Feb 2015	JHF	▪ Further revisions based on investigator and WG feedback
0.2.3	12 Feb 2015	JHF/RTI	▪ Revisions based on internal review at RTI
0.3	16 Feb 2015	Aspirin WG	▪ Updates to language re: pill packaging, anemia monitoring, sample size. Appendix 2 and 3 added.
0.3.1	19 Feb 2015	NICHD	▪ Suggestions from Menachem and Marion
0.3.2	20 Feb 2015	NICHD, MH, RD, RTI	▪ Revisions based on group review
0.3.3	24 Feb		▪ Updated based on group review
0.3.4	26 Feb	NICHD, MH, BM, JH	▪ Updates following protocol review
0.3.5	05 Mar 2015	MKT, DW, JHF	▪ Updates to Section 2.3 (study flow chart), Section 2.4.3.1 (Assigning EDD), Section 2.11 (Risk/benefit), Section 3. (Secondary analysis, DSMB/DMC description), Section 4 (form description).
0.4.0	09 March 2015	SG, MKT, JHF	▪ Revised sample consent, updated secondary outcome to include perinatal mortality.
1.0	10 March 2015	WG	▪ Final revisions to fix consistency issues. This is the version circulated to sites for IRB/EC submission.
1.1	21 March 2015	JHF	▪ Removed “perinatal mortality and morbidity” from the Section 1.1 (Primary Hypothesis or Problem).<br>▪ This version was sent to the sites to replace version 1.0 for IRB submission.
1.2	05 Dec 2016	NG	▪ Included ClinicalTrials.gov number, added protocol number, made hypertension exclusion explicit with definition, updated site information and fixed minor typographical errors.
1.3	21 Feb 2019	NG	▪ Replaced ‘heart rate greater than 110 bpm’ with ‘presence of a heartbeat’ in Section 2.2.1 Inclusion Criteria.<br>▪ Added ‘(hypertensive disorders of pregnancy)’ to Preeclampsia and eclampsia in Section 2.6 Secondary Outcomes for clarification.<br>▪ Included ‘Preterm, preeclampsia’ in Section 2.7 Other outcome of interest – Maternal outcomes.

Table of Contents

Aspirin Investigators 6

Acronyms 9

Abstract 10

1. Statement of Problem 11

1.1 Primary Hypothesis or Question 11

1.2 Secondary Hypothesis 11

1.3 Background and Rationale 11

1.3.1 Biology of Aspirin in Pregnancy 11

1.3.2 Aspirin & Preterm Birth 12

1.3.3 Aspirin & Preeclampsia 12

1.3.4 Aspirin & Growth Restriction (Small for Gestational Age) 13

1.3.5 Aspirin safety 13

1.3.6 Summary 13

2. Methods 14

2.1 Study Design 14

2.2 Study Population 14

2.2.1 Inclusion Criteria 14

2.2.2 Exclusion Criteria 14

2.3 Sequence of study activities 14

2.4 Detailed Study Procedures 16

2.4.1 Initial Screening 16

2.4.2 Consent 16

2.4.3 Ultrasound Screening 17

2.4.4 Enrollment Procedures 18

2.4.5 Monitoring 19

2.5 Primary Outcome 19

2.6 Secondary Outcomes 19
2.7 Other Outcomes of interest 20
2.8 Safety monitoring 20
2.9 Compliance Monitoring 21
2.10 Site Preparation 21
2.11 Potential Risks and Benefits to Participants 21
3. Analytical Plan 23
3.1 S tatistical Analysis Plan 23
3.1.1 Primary Analysis—Risk of Preterm (<37 weeks) Birth 23
3.1.2 Secondary and Exploratory Analysis 23
3.2 Sample Size and Power Estimates 24
3.3 Available Population 26
3.4 Projected Recruitment time 26
3.5 Study Monitoring Plan 26
3.5.1 Reporting Serious Adverse Events 26
3.5.2 Method and Timing for Reporting Serious Adverse Events 27
3.5.3 Data Monitoring Plan and Stopping Rules 27
3.6 Data Management Procedures 28
3.7 Quality Control 29
3.7.1 Training 29
3.7.2 Study Monitoring 29
3.7.3 Drug Quality Assurance and Monitoring 29
3.7.4 Ultrasound Gestational Age Dating 29

4. Data Forms 30

5. References 31

Appendix 1. Schedule of Study procedures 35

Appendix 2. Sample Informed Consent 36

Appendix 3. Outcome Definitions 40

Appendix 4. Study Timeline 42

Appendix 5. Aspirin Protocol Steering Committee and Technical Advisors 43

ASPIRIN INVESTIGATORS

Central Team: Global Network Site 08 (Belgaum, India)

Richard Derman, MD, MPH
Principal Investigator
Thomas Jefferson University
Philadelphia, PA
Richard.Derman@jefferson.edu

Bhalchandra Kodkany, MD, MBBS
Senior Foreign Investigator
KLE University's J N Medical College
Belgaum, India
drkodkany@jnmc.edu

Shivaprasad S. Goudar MD, MHPE
Co-Investigator
KLE University's J N Medical College
Belgaum, India
sgoudar@jnmc.edu

Matthew K. Hoffman, MD, MPH
Lead Investigator for Aspirin Protocol
Christiana Care
Newark, Delaware
Mhoffman@christianacare.org

Site Investigators

Global Network Site 02 (Democratic Republic of Congo)

Carl Bose, M.D. .
Principal Investigator
University of North Carolina School of Medicine
Chapel Hill, North Carolina
cbose@med.unc.edu

Antoinette Tshefu, M.D, Ph.D., M.P.H
Senior Foreign Investigator
Kinshasa School of Public Health
antotshe@yahoo.com

Global Network Site 03 (Zambia)

Wally Carlo, MD
Principal Investigator
University of Alabama at Birmingham
Birmingham, Alabama
Wcarlo@PEDS.UAB.EDU

Elwyn Chomba, MBChB, DCH, MRCP
Senior Foreign Investigator
University Teaching Hospital
Lusaka, Zambia
echomba@zamnet.zm

Global Network Site 06 (Guatemala)

Michael Hambidge, MD
Principal Investigator
University of Colorado Health Care System (UCHSC)
Denver, Colorado
Michael.hambidge@ucdenver.edu

Nancy Krebs, MD
Co-Investigator
UCHSC
Denver, Colorado
Nancy.krebs@ucdenver.edu

Ana Garces, MD, MPH
Senior Foreign Investigator
INCAP
Guatemala City, Guatemala
agarces@incap.net

Global Network Site 09 (Pakistan)

Robert L. Goldenberg, MD
Principal Investigator
Columbia University
New York, New York
rlg88@columbia.edu

Sarah Saleem, MD
Senior Foreign Investigator
Aga Khan University
Karachi, Pakistan
Sarah.saleem@aku.edu

Global Network Site 11 (Nagpur, India)

Patricia L. Hibberd, MD, PhD
Principal Investigator
Boston University
Boston, Massachusetts
plh0@bu.edu

Archana Patel, MD, DNB, MSCE
Senior Foreign Investigator
Lata Medical Research Foundation
Nagpur, India
Dr_apatel@yahoo.com

Global Network Site 12 (Kenya)

Ed Liechty, MD
Principal Investigator
Indiana University School of Medicine
Indianapolis, Indiana
eliecht@iu.edu

Fabian Esamai, MBChB, MMed, PhD
Senior Foreign Investigator
Moi University School of Medicine
Eldoret, Kenya
fesamai2007@gmail.com

National Institute of Child Health and Human Development (NICHD)

Marion Koso-Thomas, MD, MPH
Medical Officer, Global Network for Women's and Children's Health
Eunice Kennedy Shriver National Institute of Child Health and Human Development (NICHD)
kosomari@mail.nih.gov

Menachem Miodovnik, MD
Director – Global Network for Women's and Children's Health
Eunice Kennedy Shriver National Institute of Child Health and Human Development (NICHD)
menachemmiodovnik@mail.nih.gov

RTI International

Elizabeth McClure, PhD
Principal Investigator, Data Coordinating Center
mcclure@rti.org

Dennis Wallace, PhD
Co-Principal Investigator, Data Coordinating Center
Senior Statistician
dwallace@rti.org

Jay Hemingway-Foday, MPH, MSW
Protocol Manager for Aspirin Protocol
hemingway@rti.org

ACRONYMS

**ACOG**	American Congress of Obstetricians and Gynecologists
**APS**	Antiphospholipid syndrome
**ASA**	Acetylsalicylic acid
**BP**	Blood pressure
**CLASP**	Collaborative Low-dose Aspirin Study in Pregnancy
**COX**	Cyclo-oxygenase
**CRL**	Crown rump length
**DCC**	Data Coordinating Center
**DMC**	Data monitoring committee
**DMS**	Data Management System
**DRC**	Democratic Republic of Congo
**EAGER**	Effects of Aspirin in Gestation and Reproduction Study
**EDD**	Estimated due date (or estimated date of delivery)
**ERC**	Ethical review Committee
**GA**	Gestational age
**GN**	Global Network for Women's and Children's Health Research
**Hb**	Hemoglobin
**JNMC**	Jawaharlal Nehru Medical College
**IRB**	Institutional Review Board
**IUGR**	Intrauterine growth restriction
**LBW**	Low birth weight
**LDA**	Low dose aspirin
**LMP**	Last menstrual period
**MNH**	Maternal and Newborn Health
**NICHD**	Eunice Kennedy Shriver National Institute of Child Health and Human Development
**NIH**	National Institutes of Health
**NSAID**	Non-steroidal anti-inflammatory drug
**PIH**	Pregnancy induced hypertension
**PMNCH**	The Partnership for Maternal, Newborn, & Child Health
**PROM**	Premature rupture of membranes
**PTB**	Preterm birth
**RCT**	Randomized controlled trial
**RTI**	Research Triangle Institute International
**SAE**	Serious adverse event
**SFI**	Senior Foreign Investigator
**SGA**	Small for gestational age
**SPTB**	Spontaneous preterm birth
**VLBW**	Very low birth weight
**WHO**	World Health Organization

ABSTRACT

Background: Preterm birth (PTB) remains the leading cause of neonatal mortality and long term disability throughout the world. Though complex in its origins, a growing body of evidence suggests that first trimester administration of low dose aspirin (LDA) holds promise to reduce the rate of PTB substantially.

Hypothesis: First trimester administration of aspirin will reduce the risk of preterm birth.

Study Design Type: Prospective randomized, placebo-controlled, double-blinded multicenter clinical trial. Trial will be individually randomized with one-to-one ratio (intervention/control)

Population: Nulliparous women between the ages of 18 and 40, with a singleton pregnancy between 6 0/7 weeks and 13 6/7 weeks gestational age (GA) confirmed by ultrasound prior to enrollment, no more than two previous first trimester pregnancy losses, and no contraindications to aspirin. Minors who are ≥ 14 years of age may be enrolled if permitted by the country's ethical guidelines.

Intervention: Daily administration of low dose (81 mg) aspirin [also known as acetylsalicylic acid (ASA)], initiated between 6 0/7 weeks and 13 6/7 weeks GA and continued to 36 0/7 weeks GA, compared to an identical appearing placebo. Compliance and outcomes will be assessed biweekly.

Outcomes:
Primary outcome: To determine whether daily LDA initiated between 6 0/7 -13 6/7 weeks GA and continued to 36 0/7 weeks GA reduces the risk of PTB (birth prior to 37 0/7 weeks GA).

Secondary outcomes of interest are the rate of preeclampsia/eclampsia, small for gestational age (SGA), and perinatal mortality.

1 STATEMENT OF PROBLEM

1.1 PRIMARY HYPOTHESIS OR QUESTION

Our primary hypothesis is that nulliparous women with no more than two previous first trimester pregnancy losses who are treated with LDA daily beginning between 6 0/7 weeks and 13 6/7 weeks GA through 36 0/7 weeks GA will reduce the rate of preterm birth from all causes.

1.2 SECONDARY HYPOTHESIS

Women who take antenatal daily LDA initiated at 6 0/7 to 13 6/7 weeks GA will have lower rates of:

- Small for gestational age (SGA)
- Eclampsia and preeclampsia
- Perinatal Mortality

1.3 BACKGROUND AND RATIONALE

Preterm delivery, defined as delivery prior to 37 weeks 0/7 days gestation, remains the dominant cause of neonatal morbidity and mortality throughout the world[1–3] and directly leads to 28% of neonatal deaths within the first seven days of life [4] . Moreover it is responsible of up to 50% of pediatric neurodevelopmental disorders [5]. Infants born prematurely are likewise at increased risk for a variety of long term medical complications such as respiratory, gastrointestinal, cardiovascular, and metabolic disorders [6,7].

Compounding the issue, the risk of preterm birth is highest in developing countries where an estimated 12% of births are preterm compared to 5-7% in developed countries [8]. Of the near 13 million preterm births worldwide in 2005, 11 million were in Africa and Asia [9]. Given the tremendous medical, financial and emotional burden of preterm birth in the developing world and the limited resources to provide postnatal care, any interventions with the potential to reduce the rate of preterm birth deserve consideration. If one were to choose an intervention, it would be one that is widely available, inexpensive, and safe for the mother and fetus. Low dose aspirin may be just such an intervention.

1.3.1 Biology of Aspirin in Pregnancy

Aspirin is best known for its analgesic properties; however, it is well documented that aspirin is an anti-inflammatory and potent inhibitor of platelet aggregation, contributing to its anti-thrombotic effects. Both inflammation and thrombosis have been demonstrated to be the pathways responsible for the majority of preterm birth, preeclampsia and fetal growth restriction. The primary biologic effects of aspirin are mediated by inhibition of the enzyme cyclooxygenase (Cox) [10–12], which produces substances known to be involved in the defined pathways of preterm birth and placental dysfunction (Preeclampsia/growth restriction/stillbirth)[13]. By decreasing these mediators and in turn decreasing inflammation and placental dysfunction due to thrombosis, aspirin may reduce the rates of the major obstetrical complications of preterm birth, preeclampsia and growth restriction.

Equally important to the mechanisms by which Aspirin works, may be the time frame in which it is initiated. Biologically it has been demonstrated that the process of preterm birth generally begins prior to 16 weeks [13]. Likewise placental invasion of the maternal decidua is noted to occur in the first trimester and underlies the pathologic processes of preeclampsia and pathologic growth restriction. Thus aspirin may be maximally effective if initiated in the first trimester as proposed in this study.

1.3.2 Aspirin & Preterm Birth

Though the available evidence is promising, data regarding the early use of aspirin in pregnancy to prevent preterm birth are limited to two main sources: (1) Meta-analyses of prior trials and (2) a single trial of preconception aspirin (Effects of Aspirin in Gestation and Reproduction (EAGeR) Study). Multiple meta- analyses have been performed showing a limited impact on preterm birth. For example Roberge, in a meta-analysis of LDA included 22 trials that included 11,302 women, found a 19% reduction in the overall rate of preterm birth (RR 0.81, 95% CI 0.71 to 0.92) [14]. When this meta-analysis was restricted to women who initiated aspirin prior to 16 0/7 weeks a 65% reduction was observed (6 trials including 904 women, RR 0.35, 95% CI to 0.22 to 0.57).

The EAGER trial shows similar trend towards risk reduction in a cohort of 1,078 women who were randomized to LDA prior to conception. If only the original cohort (women with just one prior loss or less) is examined, there is a 55% reduction in preterm birth (RR 0.45; p-value of 0.087). When expanded to include the entire cohort (includes women with more than one fetal loss), there is a 28% risk reduction (RR of 0.72; p value 0.260). The lack of significant difference in this trial is not surprising as the primary outcome was not preterm delivery and the low risk nature of the enrolled women [15]. Nonetheless, the point estimates of the sole prospective pre conception LDA trial (EAGeR) and the multiple meta-analysis of women who initiated LDA early, suggest that LDA is associated with a decrease in the rate of PTB between28% and 65%.

1.3.3 Aspirin & Preeclampsia

Many large randomized controlled clinical trials have been conducted in both high and low risk populations (with regard to the development of preeclampsia). Duley and colleagues performed a systematic review that included 51 trials involving 36,500 women treated with antiplatelet agents for the prevention of preeclampsia [16]. Forty-four of these trials involved the use of aspirin alone (compared with placebo or no treatment) while the remainder included other treatments, often in conjunction with aspirin. Overall, the use of antiplatelet agents conferred a 19% reduction in the risk of preeclampsia (RR 0.81; 95% CI, 0.74-0.96). There was a greater risk reduction in women treated with doses greater than 75 mg/day (RR 0.49, 95% CI 0.38-0.65) compared to lower doses (RR 0.86, 95% CI 0.79-0.93). Others reviews emphasize the increased benefit from LDA in women with historical risk factors (high risk for preeclampsia) and suggest that focusing on at risk groups would decrease the number of women it is necessary to treat to prevent a single case of preeclampsia [17–19]. Like prematurity, one sees a difference in effect size based on the time of initiation. Roberge in a meta-analysis of 33 trials found a risk reduction of 0.62 (0.49 to 0.78); however, when this was restricted to women who began therapy <16 weeks the effect size was much greater (RR 0.47 (0.36 to 0.62). Of course, the "risk: benefit" ratio also is influenced by risk, which appears to be quite low for LDA. Thus, treatment of low risk women may yet prove to be justified.

1.3.4 Aspirin & Growth Restriction (Small for Gestational Age)

Low dose aspirin (LDA) also may reduce the risk of other adverse perinatal outcomes such as small for gestational age (SGA) fetus and late fetal death. In 32 trials of 24,310 women, antiplatelet therapy conferred an 8% reduction in SGA (RR 0.92, 95% CI 0.85-1.00) in women treated with the intent to prevent preeclampsia [16]. A small trial showed no benefit from LDA in women treated after the diagnosis of SGA fetus [20]. LDA also has not been effective when started after the diagnosis of preeclampsia [21,22]. Once again early initiation may be the key to success. Roberge in a like meta-analysis found a modest impact of LDA on growth restriction (RR 0.86, 95%CI 0.75-99); however when only women who initiated therapy at or before 16 weeks were examined the effect size was markedly more pronounced (0.46, 95%CI 0.33-0.66)[14].

The review by Duley and colleagues noted a 16% reduction in combined fetal, neonatal, and infant mortality in women taking antiplatelet therapy (RR 0.84, 95% CI 0.74-0.96)[23]. Perinatal death and SGA fetuses were not primary end points of these trials and results should be interpreted with caution.

1.3.5 Aspirin safety

Low dose aspirin is interesting as a potential therapy for reproductive disorders because it has a demonstrated track record of both fetal and maternal safety. Randomized controlled trials (RCTs) in thousands of women showed no increase in adverse fetal sequelae in doses < 150 mg per day [16,22,24]. In a meta-analyses of 22 studies, aspirin was not associated with an overall increase in the risk of congenital malformations [25]. The same meta-analysis reported an increase in the risk of gastroschisis (OR 2.37; 95% CI, 1.44 – 3.88) in infants exposed to high-dose aspirin (325 mg per day) in the first trimester [25]. This is biologically plausible since this malformation may be caused by vascular disruption of mesenteric vessels [26]. Results were not confirmed in a recent population based case-control study [27,28] and the association between aspirin use and gastroschisis remains uncertain. Indeed, aspirin is often used empirically to treat infertility, recurrent miscarriage and Antiphospholipid syndrome (APS). Studies regarding safety are difficult to compare due to different doses, duration, and timing of aspirin use (with regard to pregnancy). Nonetheless, the majority of data indicates minimal fetal risk from *in utero* LDA exposure. In fact, a recent study noted that LDA was actually associated with a reduction in neurobehavioral difficulties in very preterm infants [29].

1.3.6 Summary

We intend to study the effects of LDA in nulliparous women for several reasons. First, although it would be of interest to study the effect of LDA in women at high risk for preterm birth (e.g. prior preterm birth), such women may undergo interventions intended to decrease their risk of preterm birth. Thus, a study in patients with prior preterm birth would have numerous potential confounders. Conversely, multiparous women with prior term births would be at very low risk for preterm birth and also be a suboptimal population to study. Nulliparous women appear to be an ideal population since they will not undergo special interventions in an attempt to avoid preterm birth. Also, it appears that the risk of preterm birth in nulliparous women is higher than for the general obstetric population. In summary, available data suggest that LDA may be a safe, widely available and inexpensive intervention that may significantly reduce the risk of preterm birth. However, this possibility needs to be proven in a properly designed RCT with preterm birth as the primary outcome. Such a clinical trial in a racially, ethnically and geographically diverse population could best be accomplished through the established infrastructure of the Global Network for Women's and Children's Health Research (Global Network or GN).

2 METHODS

2.1 STUDY DESIGN

We propose a randomized, placebo-controlled, double-blinded multicenter clinical trial to assess the efficacy of LDA in the reduction of preterm birth. Women will be randomized equally to receive either daily LDA (81 mg) or an identical appearing placebo beginning between 6 0/7 weeks and 13 6/7 weeks GA and continuing until 36 0/7 weeks GA or delivery.

2.2 STUDY POPULATION

Studying the effect of LDA in women at very high risk for preterm birth (e.g., prior preterm birth) would have numerous confounders, as these women are likely to undergo interventions intended to decrease their risk. Conversely, multiparous women with prior term births would be at very low risk for preterm birth, making them a suboptimal study population. Nulliparous women appear to be the ideal population to study the effects of LDA since they will not undergo special interventions to avoid preterm birth. Multiple studies have shown that the risk of preterm birth in nulliparous women is higher than for the general obstetric population.

A total of 11,920 nulliparous women will be enrolled (5960 per group) across seven sites in sub-Saharan Africa, South Asia, and Latin America. For balance, each site will enroll no more than 25% of the total sample. To study the possible effect of LDA on anemia will enroll 500 women, with each site contributing at least 10% of the sample.

2.2.1 Inclusion Criteria

- Nulliparous women between 18 – 40 years of age. Minors who are ≥ 14 years of age may be enrolled if permitted by the country's ethical guidelines.
- No more than two previous first trimester pregnancy losses
- No medical contraindications to aspirin;
- Single live intrauterine pregnancy (IUP) between 6 0/7 and 13 6/7 weeks GA corroborated by an early dating ultrasound and with presence of a heartbeat.

2.2.2 Exclusion Criteria

- Women prescribed daily aspirin for more than 7 days;
- Multiple gestations;
- Fetal anomaly by ultrasound (Note most fetal anomalies are not detectable by ultrasounds done at this early gestation. Subsequent discovery of a fetal anomaly is not viewed as an exclusion.);
- Hemoglobin < 7.0 g/dl at screening;
- Systolic blood pressure ≥ 140 and diastolic ≥ 90 at screening;
- Any other medical conditions that may be considered a contraindication per the judgment of the site investigator (e.g., Lupus, Type 1 Diabetes, or any other known significant disease)

2.3 SEQUENCE OF STUDY ACTIVITIES

The sequence of study activities is described in **Figure 1**. A detailed schedule of study procedures is found in **Appendix 1.**

Figure 1. Flowchart of Study Activities

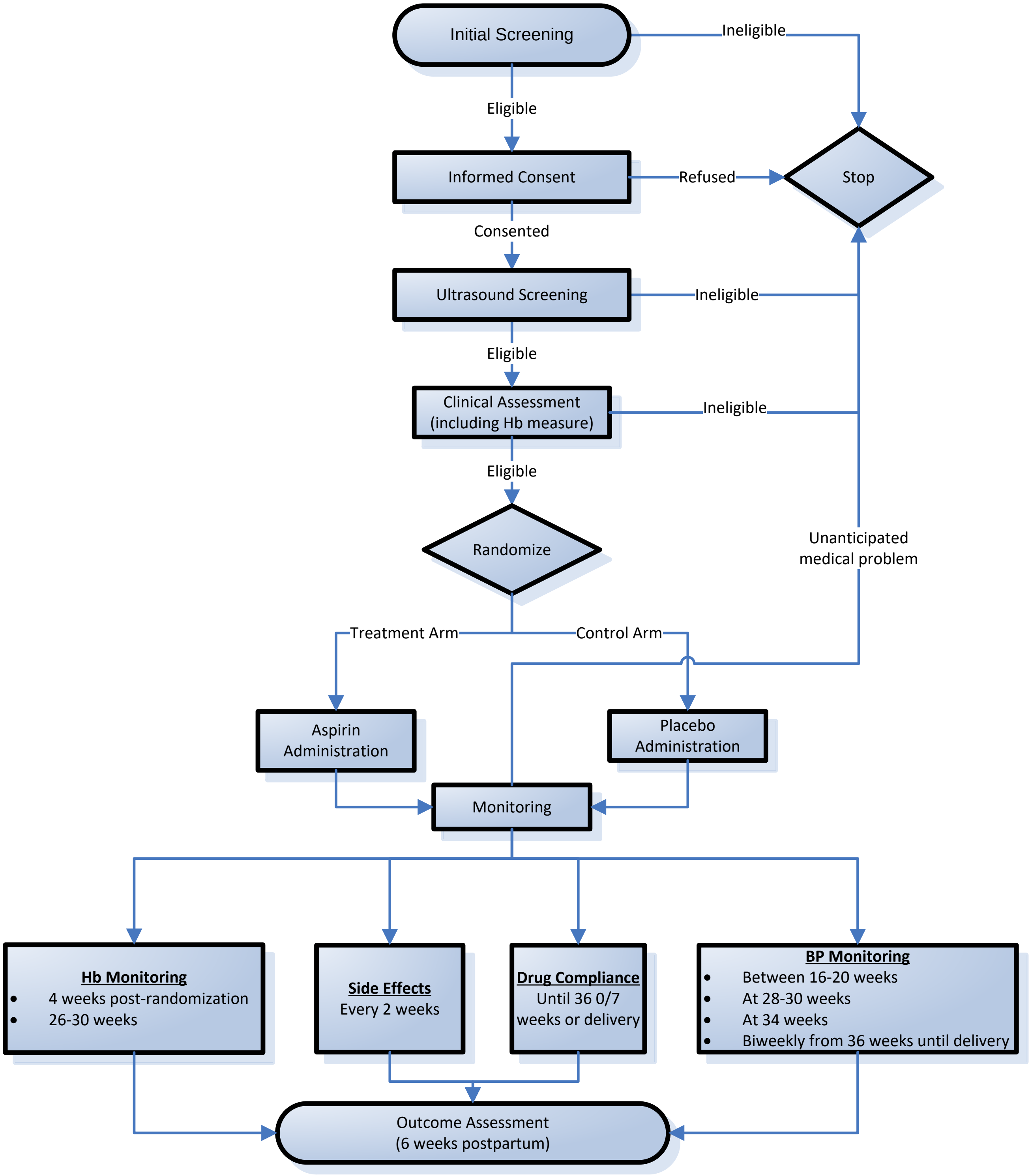

2.4 Detailed Study Procedures

2.4.1 Initial Screening

Potential study participants will be recruited from multiple sources so as to reach a diverse study population that includes nulliparous women who are pregnant. To reach the maximum number of potential participants, clinic-based and community-based recruitment methods will be used. Sites are to determine the most effective method for their site.

Upon identification, a brief assessment of eligibility will be made to determine whether the patient is nulliparous and pregnant in the required GA window based on last menstrual period (LMP), has no more than two previous 1st trimester pregnancy losses, and has no medical contraindications to aspirin or pregnancy. Recognizing that literacy may be a recruitment challenge, study staff will be provided with an obstetrical wheel to facilitate GA dating. Knowledge of exact LMP and/or GA are not absolute contraindications to study enrollment, as ultrasound will ultimately determine the participant's GA. Nonetheless, it should be noted that the rate of screened versus enrolled participants will be tracked centrally and used for study monitoring.

The initial screening will include collection of information on specific medical conditions, prior surgical procedures, medication use, allergy history, and outcomes of any prior pregnancies. If a contraindication to participation in the trial is found, the woman will be excluded from the trial at this point. If the field staff are uncertain about whether a woman is an appropriate candidate, her enrollment will be deferred until the study investigators can review her medical history. Women who meet the medical history eligibility criteria will proceed to have an ultrasound performed. Potential participants will be advised that ultrasound screening activities can be completed on a different day, as long as the woman is enrolled during the correct GA window.

2.4.2 Consent

Before a woman participates in any research activity, the research staff must obtain her informed consent to voluntarily take part in the study. Consent will be obtained from women ≥18 years of age or minors 14-17 years of age in countries where married or pregnant minors (or their authorized representatives) are legally permitted to give consent. When enrolling minors, we will follow the in-country policies for human research protection and the guidelines approved by the local ethical review committees (ERCs). In the case of pregnant minors, this may require that written consent is obtained from her parents/guardians or husband, with written assent from the minor.

The research staff will give adequate opportunity to each potential participant and/or immediate family members to read the consent form and ask questions. Recognizing that literacy levels will vary and may be a challenge, the consent process will include a verbal review of the consent form. If the participant cannot read, the form will be read aloud to her by a person unaffiliated with the study. Alternatively, the Program Coordinator or a designate may read the consent, but in the presence of a witness who is unaffiliated with the research study. Potential participants will be given an opportunity to discuss the study procedures and ask questions. Fair balance will be maintained while describing the risks and benefits of participation in the study. No undue pressure will be placed on the potential participant to enroll in the trial. It will further be explained that lack of participation will not affect the usual and anticipated standard of care.

After the potential participant has read the consent form, but before she signs, the research staff will show her a sample study pill and confirm that she is willing and able to take the study pills as prescribed. Only if she is willing to commit to taking the pills will she be enrolled; otherwise, this will be recorded as a refusal of consent. Following review of the consent and drug administration procedures, the participant (or parent/guardian) will be asked to sign the form. If the participant (or parent/guardian) is unable to sign her name, she will be asked to use her thumbprint to indicate written approval. In both cases, the unaffiliated person will also sign the consent form. Both the research staff and the study participant retain signed copies of the form. The potential participant may also take the form home to discuss with her family before signing.

An eligible woman may refuse to participate in the trial at the time of recruitment. This will be recorded in the Screening and Recruitment Form. She may also choose to withdraw from the study at any time after enrollment. This will be recorded on the Withdrawal/Termination Form. Similarly, if an enrolled woman becomes ineligible after the time of randomization, she will be withdrawn from the study and this will be recorded.

All research staff responsible for obtaining consent will be trained and certified in the protection of human subjects and the study-specific consent procedures. A model written informed consent form, developed according to the requirements of the U.S. Office of Research Protections (OHRP), is found in **appendix 2**. Each site may modify the model consent to conform to local standards, but the OHRP required elements must be maintained. The research sites will also be responsible for translating the consent form into the appropriate language(s) for their local context.

Global Network countries with legislation regarding the need to videotape consents will comply with the country regulations; however this is not part of the consent form requirements. This will not be required by protocol but rather decided by each site so as to comply with country's rules and regulations.

2.4.3 Ultrasound Screening

To be included in the study, the following three findings must be obtained via ultrasound:

- A single intrauterine gestation
- Presence of a fetal heart rate >110 beats per minute
- Crown rump length (CRL)

Ultrasound will be assessed transabdominally in all participants consistent with local good clinical practice. To obtain the CRL, a total of 3 measurements should be obtained in millimeters and the average should be utilized. Other measurements, such as the size of the gestational sac, will not be used. To determine the CRL, the sonographer is to find the mid-sagittal section of the fetus, which should be horizontal (at 90º to the angle of insonation). Ideally, the fetus should be in a neutral position (not hyperextended or flexed) and the fetal image should fill at least 30% of the monitor screen. Measurement of the CRL is done by placing the intersection of the calipers on the outer borders of the head and rump. All study sonographers will be trained to assess gestational age using CRL measurements and quality will be monitored as described in Section 3.7.4. The ultrasound screening does not need to be completed on the same day as the other screening activities, as ultrasound is likely to be conducted by different staff and location than initial screening.

2.4.3.1 Assigning Projected Estimated Due Date (EDD)

It is noted that an accurate LMP will frequently be unavailable for sizable numbers of women enrolled in this study. The assignment of the projected due date will be consistent with the recent direction provided by the NICHD consensus statement as seen in **Table 1** [30].

Table 1. Guidelines for Assigning Projected Due Date

Gestational Age by LMP	Difference between LMP and US GA	Basis for assigning projected due date
Unknown/Not available	N/A	**CRL**
< 9 0/7 weeks	> 5 days	**CRL**
	≤ 5 days	**LMP**
≥ 9 0/7 and ≤13 6/7 weeks	> 7 days	**CRL**
	≤ 7 days	**LMP**

2.4.4 Enrollment Procedures

2.4.4.1 Clinical Assessment

Following confirmation of eligibility and attainment of signed informed consent, each participant will be assessed to determine clinical status. The assessment will consist of a brief physical examination, medical history, and questions about the use of other medicines or medicinal products. It will also include a baseline hemoglobin (Hb) measurement. If the Hb measurement is < 7.0 g/dl, she will be excluded from the study.

2.4.4.2 Randomization Procedures

Randomization of subjects will be carried out to obtain a 1:1 allocation ratio between the treatment and placebo arms. Randomization will be stratified by site. A computer algorithm generated by the data coordinating center (DCC) will create the random assignment to one of the treatment arms based on randomly permuted block design with randomly varied block sizes. The block sizes will be known only by the DCC personnel.

2.4.4.3 Study Intervention and Comparison

The study intervention is 81 mg of aspirin administered daily beginning between 6 0/7 weeks and 13 6/7 weeks through 36 0/7 weeks or delivery. Though 81 mg and 75 mg doses may both be found as standard doses in developing countries, the 81 mg dose was selected because it is consistent with the dosage used in other large trials such as EAGER, is the standard in five of the seven participating sites, and introduces no additional risk over the 75 mg dose. In fact, Duley's meta-analysis showed a greater risk reduction for the development of preeclampsia associated with proteinuria in women treated with doses greater than 75 mg/day (RR 0.49, 95% CI 0.38-0.65) compared to lower doses (RR 0.86, 95% CI 0.79-0.93).[23]

Following randomization, each woman will be provided with a supply of either LDA or an identical appearing placebo. The drug or placebo will be enteric coated pills, provided in identical, child-resistant packaging, with written instructions for use. To ensure that literacy levels will not affect proper use, the study staff will provide verbal instructions at randomization and reinforce these guidelines at subsequent follow-up visits. Each woman will also receive a back-up supply of medication or placebo, which will be maintained throughout the duration of the study. The purpose of the backup is to bridge

circumstances wherein the woman misses or is late to a planned follow-up visit or the primary supply of study drug/placebo is misplaced or destroyed. Medication compliance will be monitored and the drug/placebo supply will be replenished during routine study visits.

2.4.4.4 Blinding/Masking

Both the formulation of the medication and placebo will be procured from the same manufacturer. The packaging will be standardized across sites and will be labeled as ASA 81 mg/placebo, with the expiration data and a unique identifier. A certificate of authenticity will likewise be provided.

Throughout the study, research staff and local health providers will be blinded to treatment status unless there is a serious adverse event potentially related to the treatment modality that requires unblinding for safety reasons. There will be one pharmacist at each site who will remain unmasked to monitor randomization, drug supply, and safety as needed.

2.4.5 Monitoring

Participants will meet with study staff biweekly to monitor medical side effects and other medical co-interventions. As this is a pragmatic trial, no limitations on local treatment will be prescribed but rather simply documented. An assessment of unplanned medical visits will also be made at this time. The routine study visits will also provide an opportunity to monitor drug compliance and exchange her completed drug/placebo supply for a new allotment of medication. This process will be completed until the beginning of 36 0/7 weeks GA or the participant delivers.

In order to assess the development of preeclampsia, blood pressure will be monitored at the following time points:

- Between 16-20 weeks
- At 28-30 weeks
- At 34 weeks
- Every 2 weeks beyond that until delivery, alternating with pill monitoring visits

Each visit will consist of routine blood pressure measurements using a standardized blood pressure instrument and protocol. If the blood pressure is found to be >140/90 and indicated by review of symptoms, then proteinuria will be evaluated by urine dipstick and they will be referred, as indicated.

2.5 Primary Outcome

The primary outcome of this study is preterm birth, which will be defined as delivery at or after 20 0/7 weeks and prior to 37 0/7 weeks. This will be determined based on actual date of delivery in comparison to the projected EDD, independent of whether or not the preterm delivery is indicated or spontaneous.

Primary outcome variable: Delivery at < 37 0/7 weeks gestation

2.6 Secondary Outcomes

- Preeclampsia and eclampsia (hypertensive disorders of pregnancy)
- SGA
- Perinatal mortality

2.7 Other Outcomes of interest

Maternal outcomes:

- Vaginal bleeding
- Antepartum hemorrhage
- Postpartum hemorrhage
- Maternal mortality
- Late abortion
- Change in maternal hemoglobin
- Preterm, preeclampsia

Fetal **outcomes**:

- Preterm birth <34 0/7 weeks of pregnancy
- Birth weight <2500g and <1500g
- Fetal loss
- Spontaneous abortion
- Stillbirth
- Medical termination of pregnancy

The primary, secondary, and other outcomes are defined in **Appendix 3**.

2.8 Safety monitoring

It should be noted that aspirin remains the oldest prescribed drug in human medicine. As such, the safety profile has been well described. Large longitudinal studies of both adult populations and more specifically pregnant populations have been performed, with minimal or no side effects detected. A 2014 systematic review of the use of LDA for the prevention of morbidity and mortality from preeclampsia found a 10% reduction in risk of preeclampsia, 20% reduction of Intrauterine growth restriction (IUGR), and a 14% reduction of preterm birth [31]. Further, there was no evidence of serious harms associated with LDA use during pregnancy. Bleeding-related complications, such as postpartum hemorrhage, maternal blood loss, and neonatal intracranial or intraventricular bleeding were not found. The evidence on longer-term outcomes for offspring from in utero aspirin exposure (low-dose) is limited, but follow-up data from one large RCT is reassuring [31]. Nonetheless, it should be noted these studies have not assessed the safety of aspirin in pregnancy in the developing world where unique circumstances such as endemic anemia may be present. Therefore safety monitoring remains a strong focus of this project and can be divided into three distinct areas:

1. ***Active surveillance of maternal side effects and medical complications associated with aspirin:*** Maternal surveillance will be composed of active assessment of unintended medical visits. Likewise, where obtained for clinical care, hemoglobin will be recorded as well as administration of both oral and intravenous iron. Likewise the incidence of postpartum hemorrhage, antepartum hemorrhage, cesarean delivery and maternal death due to postpartum hemorrhage will likewise be monitored on an ongoing basis.
2. ***Evaluation of fetal side effects:*** If occurrences of major fetal abnormalities or fetal loss are discovered during ultrasound procedures or follow-up visits, they will be noted and the woman will be referred according to local standard of care. Likewise, stillbirth and late abortion (delivery between 16 and 20 weeks) will be monitored via established maternal and newborn health

registry that is conducted within the Global Network infrastructure at each site. Fetal anomalies and loss will also be reviewed at least twice a year by the Data Monitoring Committee (DMC)

3. ***Evaluation of changes in maternal hemoglobin:*** Anemia will be monitored in all women, with sequential hemoglobin measurements at randomization and again between 26-30 weeks GA. Women with hemoglobin < 7.0 g/dl at randomization will be excluded from the study. Hemoglobin will also be measured at 4 weeks post-randomization in the first 500 available women who agree to participate, with changes in hemoglobin assessed by treatment group. Recognizing that site-specific characteristics may affect maternal hemoglobin, the 500 woman sample will include at least 50 women from each site. Women with hemoglobin levels of < 7.0 g/dL or with a change of more than 3.5 g/dl when measured post-randomization will be referred to a health provider to receive the local standard of care.

2.9 Compliance Monitoring

The participant will take daily LDA from the time of randomization until 36 0/7 weeks GA or delivery. Drug compliance will be routinely monitored throughout this period through pill counts and the completion of a medicine compliance form each time the pill supply is replenished. An interval medical obstetrical history will also be taken during these scheduled visits. Study staff will also provide reminders about proper drug administration and the importance of compliance during bi-weekly visits to monitor side effects.

2.10 Site Preparation

In preparation for study implementation, the sites will meet with health authorities and conduct community sensitization activities to ensure that study procedures are appropriate for the local context and to encourage commitment and engagement at the facility and community level. Site preparation activities will focus on:

- Identifying and hiring study staff;
- Developing site-specific procedures for safety monitoring (blood pressure, anemia) and procuring the necessary equipment;
- Exploring locally-acceptable methods to monitor and improve medication compliance.
- Identifying potential implementation challenges and developing culturally-appropriate solutions.
- Identifying local medicines/treatments that contain aspirin or have contraindications for its use.
- Educating health workers and community members on the use of aspirin in pregnancy (including safety of aspirin use)

2.11 Potential Risks and Benefits to Participants

There are several potential direct and indirect benefits of this trial. In developing countries, including those of Global Network partners, the rate of preterm births can be as high as 19% of all deliveries amounting to an estimated 15 million preterm births in 2012 [1,9]. Should this trial be successful and the rate of preterm deliveries be dropped by 20%, we could eliminate 3,750,000 of these early births per year. Aspirin has also been shown to decrease the incidence of stillbirth and delivery of SGA infants [20,21,32,23]. In addition to the reduction of preterm birth, there is clear evidence of maternal benefit. There are data supporting the use of daily LDA to stave off preeclampsia[23,33,34]. This is an important benefit, as

pregnancy induced hypertension (PIH) is a major contributor to maternal mortality [35]. This benefit is described in more detail in **Section 1.3.3**.

By reducing the number of preterm deliveries, the trial also has the potential to decrease need for extended and recurrent hospital stays that are frequent in this population, as well as impacting the cost of care for the long-lasting neurodevelopmental disorders and chronic health conditions incurred later on in life [5–7]. Moreover, with this simple strategy, we could potentially save the lives of over 1 million neonates that die due to prematurity every year[1]. Globally, preterm delivery remains a major health care financial burden [1,36]

The efficacy and side effect profile of aspirin is well-established and thoroughly investigated. Women with a known adverse reaction to acetylsalicylic acid (aspirin) will be excluded from the trial. This occurs rarely, but an allergic reaction to the intervention medication remains a small risk. Fetal concerns are also limited. Though the risk of congenital malformations has been mostly negated by a meta-analysis [25], a conflicting case-control study demonstrated an association between high-dose aspirin use and gastroschisis [26]. Most notably, LDA has actually been shown to improve neurologic outcomes in preterm infants [29]. In light of such findings, the medical community acknowledges that antepartum exposure to low dose aspirin incurs only marginal fetal risk. Overall, the profound benefits of this intervention greatly outweigh the minimal risks to both mother and child.

3 ANALYTICAL PLAN

3.1 STATISTICAL ANALYSIS PLAN

Prior to conducting formal analyses baseline demographic characteristics and key clinical measures will be compared between the women in the two treatment arms using contingency table approaches for categorical variables and analysis of variance models and t-tests for continuous variables. For all of these analyses, comparisons will be made within Global Network site and overall across the sites controlling for site in the models.

3.1.1 Primary Analysis—Risk of Preterm (<37 weeks) Birth

The primary analysis will compare the risk of preterm birth between the two treatment arms using two complementary approaches. First, the formal test of the primary hypothesis that the risk of preterm birth differs between the two arms will utilize a modified intention-to-treat approach based on a Cochran-Mantel-Haenszel test stratified by Global Network sites. The approach is characterized as a modified intention to treat approach because the analysis population for the test will be all randomized pregnancies for which the delivery occurs after 20 weeks. Earlier deliveries (miscarriages) will be considered missing completely at random for purposes of this analysis.

In order to evaluate the sensitivity of the primary hypothesis test to the assumption by which miscarriages are treated as randomly missing and to control for potential confounders of the treatment effect, additional analyses will be conducted using a series of three generalized linear models. These generalized linear models will be fit with the binary outcome of preterm (<37 week delivery) as the outcome measure. The initial model will include terms for treatment, site, and a treatment by site interaction. If the treatment by site interaction term is found to be significant ($p<0.05$), then all subsequent models will include the interaction term and all effects will be reported by site. If the interaction term is not significant, it will be removed from the model and this initial model be used to estimate an unadjusted treatment effect controlling for site. The second series of models will include any demographic or clinical variable found to differ significantly between the treatment arms in the preliminary analyses described above. These models will be used to generate adjusted estimates of the treatment effect controlling for potential confounders. The third set of models will utilize extensions of the generalized linear model that incorporate inverse probability weighting to evaluate the sensitivity of the inference to treatment of miscarriages as randomly missing [37].

3.1.2 Secondary and Exploratory Analysis

This study has been designed to evaluate formally the differences between the two treatment arms for three secondary outcomes: the risk of perinatal mortality; the risk of eclampsia/preeclampsia, and the risk of a SGA delivery. As with the primary analyses, each of these outcomes will be examined individually using formal tests of hypotheses that the risk differ between the two arms based on Cochran-Mantel-Haenszel tests stratified by Global Network sites. The analyses for each of these 3 secondary outcomes will utilize the same modified intention-to-treat population used for the primary outcome. Each of these hypothesis tests will be conducted at the 0.05 level of significance with no adjustment for multiple comparisons. In addition to this set of formal hypothesis tests, model based analyses comparable to those conducted for the primary analyses will be used to evaluate potential heterogeneity of treatment effect across sites and evaluate potential confounding. For each of the 3

outcomes, an initial model will include terms for treatment, site, and a treatment by site interaction. If the treatment by site interaction term is found to be significant ($p<0.05$), then all subsequent models will include the interaction term and all effects will be reported by site. If the interaction term is not significant, it will be removed from the model and this initial model be used to estimate an unadjusted treatment effect controlling for site. The second series of models will include any demographic or clinical variable found to differ significantly between the treatment arms in the preliminary analyses described above. These models will be used to generate adjusted estimates of the treatment effect controlling for potential confounders.

In addition to these planned formal secondary outcome analyses, we will also conduct exploratory outcomes for a number of other binary outcomes. The analytic approach for these exploratory analyses, will be comparable to that planned for the primary and formal secondary analyses but will focus on estimation of effect sizes and generation of hypotheses planned for this study are all binary outcomes, therefore the analytic approach will be comparable to the approach described for the primary analysis. Initially, contingency tables will be used to generate estimates of the risk of each outcome for the two treatment arms, both overall and separately by Global Network (GN) site and across all sites, and the relative risk associated with treatment will be estimated by site and across sites controlling for site. For each outcome a series of generalized linear models analogous to those described for the primary outcome to generate unadjusted estimates of risk, estimates of risk adjusted for potential confounders, and estimates of risk adjusted to account for any possible differences in risk between the arms associated with miscarriages.

3.2 Sample Size and Power Estimates

The risk of preterm birth < 37 weeks gestation in untreated nulliparous women was estimated to be about 8.0% for the EAGeR trial [38,15]. Unpublished data for 2012 from the GN sites show preterm birth rates, ranging from 2.9% to 9.8%, while WHO estimates the range for the countries in which GN sites are located to be 7.7% to 16.7% [1]. In developing sample size estimates we considered risks in the range of 8% to 14% and ultimately selected sample sizes based on a conservative estimate of 8%. Because the association of LDA and preterm birth may differ in international settings, we examined sample size requirements for reductions of 40%, 25%, and 20%. As shown in **Table 2** below, the number of evaluable participants needed to detect a 20% reduction from a usual rate of 8% preterm births with 90% power is 5,483 per treatment arm. To account for the loss of evaluable subjects due to miscarriages, which are anticipated to occur in approximately 5% of participants) and loss to follow-up (assumed to be in the 1% to 2% range), sample sizes were increased by 8% to obtain a final sample size of 11,920 (or 5,960 per treatment arm).

The Global Network has access to approximately 68,000 births per year. It is estimated that 40% would be nulliparous, yielding 23,800 potential pregnancies per year. With an enrollment rate of one third of eligible women, this trial could easily be accomplished by the Global Network within an 18 month enrollment period.

Table 2. Evaluable Sample Size Estimates

PTB rate in Placebo Arm	PTB rate in ASA	% Red	Sample size per group	PTB rate in ASA	% Red.	Sample size per group	PTB rate in ASA	% Red.	Sample size per group
Power=80% (α=0.05; β=0.2)									
8%	4.8%	40%	918	6.0%	25%	2,554	6.40%	20%	4,096
10%	6.0%	40%	721	7.5%	25%	2,005	8.00%	20%	3,213
12%	7.2%	40%	591	9.0%	25%	1,638	9.60%	20%	2,625
14%	8.4%	40%	497	10.0%	25%	1,377	11.20%	20%	2,204
Power=90% (α=0.05; β=0.1)									
8%	4.8%	40%	1,228	6.0%	25%	***3,419***	6.40%	20%	***5,483***
10%	6.0%	40%	965	7.5%	25%	2,683	8.00%	20%	4,301
12%	7.2%	40%	790	9.0%	25%	2,193	9.60%	20%	3,513
14%	8.4%	40%	665	10.5%	25%	1,842	11.20%	20%	2,950

Sample Size—Secondary Outcomes: One of the key secondary objectives of this trial is to evaluate the effect of low dose aspirin on perinatal mortality. We evaluated the reduction in perinatal mortality that could be detected with 80% power and 90% power under the assumption that the sample size for the study would be based on that needed to achieve the primary aim of demonstrating a 20% reduction in low-birth weight infants. The analyses were based on the assumption that the perinatal mortality rate in the GN population is approximately 45 perinatal deaths per 1000 deliveries (unpublished data from the MNH Registry for 2013). With this estimated underlying mortality rate and 5,483 evaluable subjects per treatment arm required to demonstrate the 20% reduction in low birth weight prevalence as outlined in the primary aim, the study will have 80% power to detect a 24% reduction in perinatal mortality and 90% power to detect a 27% reduction in perinatal mortality.

To determine the sample size actually needed to demonstrate the reduction in perinatal mortality actually expected if the intervention results in a 20% reduction in prevalence of low birth weight infants, we examined both the prevalence of low birth weight infants across the GN sites and the perinatal mortality risk in both the low birth weight and normal birth weight infants. Based on 2013 MNH Registry data, the prevalence of low birth weight among all GN deliveries was 16.3% and the perinatal mortality rates were 20.5 per 1000 deliveries among normal birth weight infants and 162.3 per 1000 deliveries among low birth weight infants; these values yield a weighted average perinatal mortality rate of 43.6 per 1000 deliveries. If the low dose aspirin intervention is effective in reducing the prevalence of low birth weight deliveries by 20% (i.e. reducing the prevalence from 16.3% to 13%) and the conditional risk of perinatal mortality is unchanged in the low birth weight and normal birth weight cohorts, we would expect the resultant overall perinatal mortality rate to be reduced to 38.9 per 1000 deliveries or a 10.8% reduction. Detection of a reduction of this magnitude with 80% power would require an evaluable sample size of greater than 28,000 evaluable deliveries per treatment arm, and the study would need over 37,000 evaluable deliveries per arm to achieve 90% power to detect a reduction of this level in perinatal mortality.

Given the sample sizes associated with the second approach, the best alternative appears to be to power the study to demonstrate the 20% reduction in prevalence of low birth weight deliveries with a commitment to generate point and interval estimates of the impact of the intervention on perinatal

mortality. By examining the risk of perinatal mortality in both the normal and low birth weight infants in the two arms, the trial would provide some insight about impacts of the intervention on mortality other than that mediated through the effect on birth weight.

We also conducted power analyses to examine the effect sizes that could be detected with the planned sample sizes for two additional secondary outcomes, prevalence of SGA infants and incidence of eclampsia/preeclampsia in the target population. Generic information as well as preliminary information from the 2013 MNH Registry indicates that the risk of each of these events in the population of interest is approximately 5%. Under the assumption of 5483 evaluable participants in each treatment arm and an assumed Type I error rate of 0.05 for per-comparison analyses (i.e. no control for multiple comparisons in the analyses of these secondary outcomes), the study will have 70% power to detect a 20% reduction in the risk of each of these outcome measures, 85% power to detect a 24% reduction and 90% power to detect a 26% reduction. Consequently, the study is reasonably powered to examine these secondary outcome measures.

3.3 AVAILABLE POPULATION

The sites of the Global Network have access to approximately 68,000 deliveries annually with 24,000 being potentially eligible pregnancies for this study. This diverse pregnant population is highly accessible and one which, in our international experience, has been eager to participate in non-invasive studies to promote the well-being of both mother and child. The Global Network sites estimate that as few as one third and as many as one half of eligible women will enroll in this protocol, which will allow the study to meet recruitment goals within 18 months.

3.4 PROJECTED RECRUITMENT TIME

Each site will begin recruitment after their site-specific regulatory approvals are in place, the site has been adequately prepared, and training is completed. This will require a staggered start, as the timeline for these activities will vary per site. The projected timeline for this study is approximately 3 years (36 months). This includes a 12 month preparatory phase with 3 months for training, an 18 month recruitment period, and 6 months for data cleaning and analysis. A detailed study timeline is found in **Appendix 4.**

3.5 STUDY MONITORING PLAN

3.5.1 Reporting Serious Adverse Events

Serious Adverse events (SAEs) will be monitored continuously using a special form that will be required for any event that meets the following criteria:

- Results in death;
- Is life-threatening;
- Requires hospitalization or prolongs existing hospitalization;
- Results in persistent or significant disability or incapacity;
- Any other serious or unexpected adverse event that the study investigator(s) feels should be reported.

The specific adverse events to be monitored in this trial include maternal death, upper GI bleeding (vomiting blood), fetal anomaly, gastroschisis, post-partum hemorrhage, or antepartum hemorrhage. The occurrence of any of these events will also trigger completion of the Serious Adverse Events Form.

3.5.2 Method and Timing for Reporting Serious Adverse Events

The Senior Foreign Investigator (SFI) must report the following SAEs by emailing or faxing a copy of the form to RTI as follows:

Within 48 hours of SFI's notification of the event:

- All maternal deaths
- All SAEs with a definite or suspected/probable relationship to the intervention

Within 7 days of SFI's notification of the event:

- All life-threatening events
- All SAEs considered to have a probable or possible relationship to the intervention.

All emailed or faxed forms should also be entered into the DMS and transmitted within 7 days as a back-up to ensure no SAE is missed. Additional reporting procedures include:

- RTI will forward all SAEs to the US-based Principal Investigator (PI) and NIH for further assessment of relationship to study intervention.
- The PI and SFI will be responsible for reporting to their respective IRB and other regulatory authorities per their institutional policy.
- RTI will be responsible for reporting SAEs to the DMC bi-annually at a minimum. The frequency of reporting to the DMC may be increased if the reported events or interim data reviews by the DMC indicate that more frequent safety monitoring is needed.

Any SAE considered unrelated to the intervention is not required to be reported in an expedited manner. These events should be entered into the data management system and transmitted per routine procedures.

3.5.3 Data Monitoring Plan and Stopping Rules

All the Global Network sites will report data to the Global Network Data Coordinating Center, located at RTI International. The data will be used to evaluate protocol adherence and site performance (e.g., recruitment, loss to follow-up, data quality). The DCC will provide standardized progress reports to NICHD and the site investigators on a monthly basis to monitor outcome variables and adverse events.

Oversight of the trial will be handled by two principal groups with different focuses:

1) **Protocol-focused Steering Committee (SC):** The SC is comprised of the Central Study Team from Christiana Care and Jawaharlal Nehru Medical College (JNMC), NICHD, the DCC, and investigators from each of the participating sites (see **Appendix 5**). The Central Study Team, with assistance from NICHD and the DCC, will have primary responsibility for overall study design, development of study materials and procedures, and oversight of study implementation. They will meet via conference call bi-weekly to monitor study progress and ensure proper implementation of the trial. The Site Investigators will be responsible for providing guidance on study design, developing site-specific implementation plans, ensuring study staff are properly trained, and providing oversight of the

study at the site level. The SC will convene via conference call at least once per quarter and will meet in person twice a year to discuss study design and implementation issues. Members of the Central Study team, NICHD, and RTI will also conduct site visits, as the budget allows, to bolster enthusiasm, provide hands-on training and education to the participating staff, and address site-specific issues, if any.

2) **Data Monitoring Committee (DMC)**: The DMC, a standing group that monitors all NICHD-funded Global Network studies, will be responsible for ensuring safe and ethical treatment of study participants through monitoring of the study. The membership will include, at a minimum, a statistician, obstetrician, pediatrician and an expert in international health. The DMC designated by NICHD will review the data collected at approximate 6 month intervals throughout the course of the study. The DMC reports, which are prepared by the Data coordinating center, will include information on study enrollment rates and participant progress through the study, participant compliance with protocol-specified treatment regimens, protocol violations, adverse events, and efficacy outcomes. The focus of the DMC review will be on monitoring participant safety and study progress/futility but data on treatment effectiveness will also be presented to frame the DMC discussions on safety and futility. However, no formal interim analysis of efficacy or effectiveness are planned and the DMC will not be responsible for stopping the trial for efficacy. The DMC will be charged with monitoring adverse events and side effects from LDA. All known associated side effects and specific obstetric or fetal concerns will be considered reportable to the DMC. The study will be reviewed by the DMC bi-annually at a minimum, but may be reviewed more frequently if concerns are raised about participant safety or about adequate process of the study.

3.6 Data Management Procedures

Data will be collected both prospectively and from existing clinical records, using hard copy forms or Android Tablets. Regardless of data capture methodology, all data will be kept confidential. Each participant will be assigned a unique study ID which will be used to identify the participant. Only the screening log will contain the name (which is not transmitted). If hard copy forms are used, they will be retained in a secure location for possible editing or queries at the central data entry site. Data will be entered into computers using the Data Management System (DMS) developed by RTI and the assigned study number. The DMS will also allow site staff to produce project reports and backup the study database. Electronic data will be transferred from each data management computer to a single Research Unit Data Center (RUDC) in each country, creating a complete data repository. At least once a week, data will be transmitted from the RUDC to the DCC at RTI. The data center will conduct training on the DMS system, as needed, and will maintain the central database for the study.

Precision and accuracy of actual data collected will be checked by chart review (random 5%) and internal procedures using the computer program. Monthly audits and incomplete data reports will be performed by a review team consisting of at least of the SFI and the country coordinator. Data editing and error resolution will be performed monthly. In addition, a sample of participants will be visited to confirm their participation, with procedures determined per site. These activities will be shared between the site and the data center.

3.7 QUALITY CONTROL

3.7.1 Training

All study personnel must participate in training on the proper implementation of study procedures and the ethics of conducting research with human subjects before beginning any research activity. The SFI and project coordinator will ensure that all study personnel receive the appropriate training, and obtain the required certification ensuring that they have met the training objectives. RTI will be responsible for developing a certification test. The SFI and project manager will be responsible for overseeing the certification process.

3.7.2 Study Monitoring

Major monitoring responsibilities of the PI/SFI, assisted by the country coordinator, are (1) confirming proper IRB approval; (2) monitoring the delivery of the study intervention; (3) assessing and evaluating the quality of study implementation; (4) ensuring compliance with the intervention, including proper randomization; (5) evaluating accuracy, precision, and completeness of data collected, entered, and transmitted (along with the DCC); (6) ensuring that all personnel are fulfilling their obligations; (7) maintaining morale and enthusiasm of the staff; (8) handling ad hoc problems and maintaining communication; (9) ensuring inter-site consistency; and (10) proposing improvements to the monitoring activities.

NICHD and the DCC staff will conduct site visits as needed. These visits will include review of individual participant records, including supporting data, to ensure protection of study participants, compliance with the protocol, and accuracy and completeness of records. The SFI/PI will make study documents (e.g., logbooks, data forms, staff training certificates) and pertinent hospital/clinic records readily available for inspection by the local IRB, site monitors, and the NICHD for confirmation of the study data.

3.7.3 Drug Quality Assurance and Monitoring

The study drug manufacturer will have a Good Manufacturing Practices (GMP) designation vetted by the FDA and a certificate of authenticity will be provided. Each site will adapt best practice guidelines for drug shipment and storage to the needs and infrastructure of their local environment. Study staff will be trained in on the drug shipment and storage plan to ensure that best practices are maintained at all time. Additionally, participants will receive detailed instruction on proper storage of the study drug at home. Drug stability information will be maintained throughout the study. For quality assurance, a sample of pills from each site will be randomly selected and tested for bioavailability at multiple time points during the study period. A sample from each batch will be tested.

3.7.4 Ultrasound Gestational Age Dating

All study sonographers will be trained to assess gestational age using CRL measurements. A sample of ultrasound images at each site will be reviewed by study investigators to ensure that CRL is accurately measured and gestational age is accurately assigned. If discrepancies are found, the sonographers will be retrained. Ultrasound will be used only for pregnancy dating and no other purpose.

4 DATA FORMS

The following forms will be used for this study:

Form Description	Purpose	Key Data Elements	Data Source
Screening Log	To track screening and enrollment at facility or community level	Contact information, screening and enrollment date and status	Patient report
Initial Screening and Recruitment	To determine eligibility and record consent status	Screening criteria: age, pregnancy history, estimated GA by LMP, consent status/date.	Patient report, obstetrical wheel,
Ultrasound Screening	To confirm eligibility criteria by ultrasound.	GA dating, assessment of fetal anomaly, singleton pregnancy, and fetal heart rate	Ultrasound
Hemoglobin (Hb) Monitoring	To monitor Hb at enrollment and 26-30 GA. It will also capture Hb at 4 weeks post-randomization in subset of 500 women	Hemoglobin	Lab test
Clinical Assessment	To assess clinical status through examination and patient history and provide information on medicine distribution/use	Height, weight, BP, Pulse, patient-reported medical history, use of other medications	Clinical exam, lab tests, patient report
Biweekly Monitoring	To monitor side effects, use of other medications, and drug compliance.	Drug compliance, BP, side effects (nausea, vomiting, rash/hives, diarrhea, vaginal bleeding, gastritis, other), use of other medicines, care seeking, pregnancy complications.	Pill count, patient report, clinical examination
Blood Pressure Monitoring	To monitor BP for active identification of pre-eclampsia	BP, urine protein (if indicated), fetal movement, diagnosis of eclampsia or stroke.	Clinical examination, patient report
Unscheduled Emergency Visit	To monitor unscheduled medical visits or medical emergencies	Patient status, pulse, BP, reason for medical visit/emergency, pill count and patient report on drug compliance, presence of side effects, referrals	Existing medical records, patient reports
Severe Adverse Events	To record fatal, life-threatening, or any other serious, unexpected adverse event	Date/time of event, date/time of resolution, nature of adverse event, management of adverse event, attribution to study (yes/no).	Patient reports, existing medical records
Study Withdrawal	To describe reasons for study termination or withdrawal	Date and reason for termination/withdrawal.	Patient report
Protocol Deviation	To record protocol deviations and corrective actions	Date and nature of deviation/violation, corrective action.	Patient report, study records
Outcomes	Maternal and Fetal outcomes will be documented via the GN's established MNH Registry	Preterm birth (primary outcome); maternal and fetal secondary outcomes.	MNH Registry

5 References

1. March of Dimes, PMNCH, Save the Children, WHO. *Born Too Soon: The Global Action Report on Preterm Birth*. Geneva: World Health Organization; 2012.

2. Mathews TJ, Menacker F, MacDorman MF. Infant mortality statistics from the 2002 period: linked birth/infant death data set. *Natl Vital Stat Rep*. 2004;53:1-29.

3. Anderson RN, Smith BL. Deaths: leading causes for 2001. *Natl Vital Stat Rep*. 2003;52:1-85.

4. Beck S, Wojdyla D, Say L, et al. The worldwide incidence of preterm birth: A systematic review of maternal mortality and morbidity. *Bull World Health Organ*. 2010;88:31-38. doi:10.2471/BLT.08.062554.

5. Goldenberg R, Rouse D. Prevention of Premature Birth. *N Engl J Med*. 1998;339(5):313-320.

6. McCormick MC, Richardson DK. Premature infants grow up. *N Engl J Med*. 2002;346(3):197-198. doi:10.1056/NEJM200201173460310.

7. Saigal S, Doyle LW. An overview of mortality and sequelae of preterm birth from infancy to adulthood. *Lancet*. 2008;371:261-269. doi:10.1016/S0140-6736(08)60136-1.

8. Valero De Bernabé J, Soriano T, Albaladejo R, et al. Risk factors for low birth weight: A review. *Eur J Obstet Gynecol Reprod Biol*. 2004;116:3-15. doi:10.1016/j.ejogrb.2004.03.007.

9. Blencowe H, Cousens S, Oestergaard MZ, et al. National, regional, and worldwide estimates of preterm birth rates in the year 2010 with time trends since 1990 for selected countries: a systematic analysis and implications. *Lancet*. 2012;379:2162-2172. doi:10.1016/S0140-6736(12)60820-4.

10. FitzGerald G a., Oates J a., Hawiger J, et al. Endogenous biosynthesis of prostacyclin and thromboxane and platelet function during chronic administration of aspirin in man. *J Clin Invest*. 1983;71(November 1982):676-688. doi:10.1172/JCI110814.

11. Chavarría ME, Lara-González L, González-Gleason A, García-Paleta Y, Vital-Reyes VS, Reyes A. Prostacyclin/thromboxane early changes in pregnancies that are complicated by preeclampsia. *Am J Obstet Gynecol*. 2003;188:986-992. doi:10.1067/mob.2003.203.

12. Yu Y, Cheng V, Fan J, et al. Differential impact of prostaglandin H synthase 1 knockdown on platelets and parturition. *J Clin Invest*. 2005;115:986-995. doi:10.1172/JCI200523683.

13. Goldenberg RL, Culhane JF, Iams JD, Romero R. Epidemiology and causes of preterm birth. *Lancet*. 2008;371:75-84. doi:10.1016/S0140-6736(08)60074-4.

14. Roberge S, Nicolaides KH, Demers S, et al. Prevention of perinatal death and adverse perinatal outcome using low-dose aspirin: a meta-analysis. *Ultrasound Obstet Gynecol*. 2013;41(January):491-499. doi:10.1002/uog.12421.

15. Schisterman EF, Silver RM, Lesher LL, et al. Preconception low-dose aspirin and pregnancy outcomes: Results from the EAGeR randomised trial. *Lancet*. 2014;384:29-36. doi:10.1016/S0140-6736(14)60157-4.

16. Duley L, Henderson-Smart DJ, Knight M, King JF. Antiplatelet agents for preventing and treating pre-eclampsia. *Cochrane Database Syst Rev*. 2004;(4):CD000492. doi:10.1002/14651858.CD000492.

17. Coomarasamy A, Braunholtz D, Song F, Taylor R, Khan KS. Individualising use of aspirin to prevent pre-eclampsia: A framework for clinical decision making. *BJOG An Int J Obstet Gynaecol*. 2003;110(October):882-888. doi:10.1111/j.1471-0528.2003.03002.x.

18. Coomarasamy A, Honest H, Papaioannou S, Gee H, Khan KS. Aspirin for prevention of preeclampsia in women with historical risk factors: A systematic review. *Obstet Gynecol*. 2003;101:1319-1332. doi:10.1016/S0029-7844(03)00169-8.

19. Heyborne KD. Preeclampsia prevention: Lessons from the low-dose aspirin therapy trials. *Am J Obstet Gynecol*. 2000;183:523-528. doi:10.1067/mob.2000.106757.

20. Newnham JP, Godfrey M, Walters BJ, Phillips J, Evans SF. *Low Dose Aspirin for the Treatment of Fetal Growth Restriction: A Randomized Controlled Trial.*; 1995:370-374.

21. Italian Study of Aspirin in Pregnancy. Low-dose aspirin in prevention and treatment of intrauterine growth retardation and pregnancy-induced hypertension. *Lancet*. 1993;341(8842):396-400.

22. CLASP. CLASP: a randomised trial of low-dose aspirin for the prevention and treatment of pre-eclampsia among 9364 pregnant women. CLASP (Collaborative Low-dose Aspirin Study in Pregnancy) Collaborative Group. *Lancet*. 1994;343:619-629. doi:10.1016/0091-2182(94)90141-4.

23. Duley L, Henderson-Smart DJ, Knight M, King JF. Antiplatelet agents for preventing and treating pre-eclampsia. *Cochrane Database Syst Rev*. 2007;(2):CD000492. doi:10.1002/14651858.CD000492.

24. Knight M, Duley L, Henderson-Smart DJ, King JF. Antiplatelet agents for preventing and treating pre-eclampsia. *Cochrane Database Syst Rev*. 2000:CD000492. doi:10.1002/14651858.CD000492.

25. Kozer E, Nikfar S, Costei A, Boskovic R, Nulman I, Koren G. Aspirin consumption during the first trimester of pregnancy and congenital anomalies: A meta-analysis. *Am J Obstet Gynecol*. 2002;187:1623-1630. doi:10.1067/mob.2002.127376.

26. Werler M, Sheehan JE, Mitchell AA. Maternal medication use and risks of gastroschisis and small intestinal atresia. *Am J Epidemiol*. 2002;155:26-31. doi:10.1093/aje/155.1.26.

27. American College of Obstetricians and Gynecologists. ACOG committee opinion no. 561: Nonmedically indicated early-term deliveries. *Obstet Gynecol*. 2013;121:911-915. doi:10.1097/01.AOG.0000428649.57622.a7.

28. Norgard B, Puho E, Czeizel AE, Skriver M V, Sorensen HT. Aspirin use during early pregnancy and the risk of congenital abnormalities: a population-based case-control study. *Am J Obs Gynecol*. 2005;192:922-923. http://www.ncbi.nlm.nih.gov/pubmed/15746692.

29. Marret S, Marchand L, Kaminski M, et al. Prenatal low-dose aspirin and neurobehavioral outcomes of children born very preterm. *Pediatrics*. 2010;125:e29-e34. doi:10.1542/peds.2009-0994.

30. American College of Obstetricians and Gynecologists. Method for Estimating Due Date. Committee Opinion 611. *Obs Gynecol*. 2014;124:863-866.

31. Henderson J, Whitlock E, O'Connor E, Senger C, Thompson J, Rowland M. Low-Dose Aspirin for the Prevention of Morbidity and Mortality From Preeclampsia : A Systematic Evidence Review for the U . S . Preventive Services Task Force. Evidence Synthesis No. 112. *Rockville, MD Agency Healthc Res Qual*. 2014;14-05207-E(112).

32. Beroyz G, Casale R, Farreiros A, et al. CLASP: A randomised trial of low-dose aspirin for the prevention and treatment of pre-eclampsia among 9364 pregnant women. *Lancet*. 1994;343:619-629. doi:10.1016/S0140-6736(94)92633-6.

33. Vainio M, Kujansuu E, Iso-Mustajärvi M, Mäenpää J. Low dose acetylsalicylic acid in prevention of pregnancy-induced hypertension and intrauterine growth retardation in women with bilateral uterine artery notches. *BJOG An Int J Obstet Gynaecol*. 2002;109:161-167. doi:10.1111/j.1471-0528.2002.01046.x.

34. Villa PM, Kajantie E, Räikkönen K, et al. Aspirin in the prevention of pre-eclampsia in high-risk women: A randomised placebo-controlled PREDO Trial and a meta-analysis of randomised trials. *BJOG An Int J Obstet Gynaecol*. 2013;120:64-74. doi:10.1111/j.1471-0528.2012.03493.x.

35. Steegers EAP, von Dadelszen P, Duvekot JJ, Pijnenborg R. Pre-eclampsia. *Lancet*. 2010;376:631-644. doi:10.1016/S0140-6736(10)60279-6.

36. Kulberg CH. The Cost of Poor Birth Outcomes. 2013. http://www.theplanningcouncil.org/inp/5Wellness/2LowBirthWeight/TheCostofPoorBirthOutcomes.pdf.

37. Hogan JW, Lancaster T. Instrumental variables and inverse probability weighting for causal inference from longitudinal observational studies. *Stat Methods Med Res*. 2004;13:17-48. doi:10.1191/0962280204sm351ra.

38. Abramovici A, Cantu J, Jenkins SM. Tocolytic Therapy for Acute Preterm Labor. *Obstet Gynecol Clin North Am*. 2012;39:77-87. doi:10.1016/j.ogc.2011.12.003.

APPENDIX 1. SCHEDULE OF STUDY PROCEDURES

	6 0/7- 13 6/7 weeks GA	Randomization /Enrollment	Bi-weekly after enrollment	4 weeks after enrollment	monthly after enrollment	16-20 weeks GA	26-30 weeks GA	34 weeks GA	36 weeks GA until delivery	6 weeks Post-Partum	As needed
**Screening**											
• Initial eligibility	X										
• Ultrasound	X										
**Consent**	X										
**Clinical Assessments**											
• Physical exam		X									
• Medical history		X									
• Use of other medicines		X	X								
• Maternal Hb monitoring		X		X			X				
• BP Monitoring		X				X	X	X	X		
**Medication Activities**											
• Instructions for drug use		X									
• Medication compliance			X								
• Resupply					X						
**Outcome Assessment**											
• Preterm Birth										X	
• SGA										X	
• Preeclampsia/ Eclampsia			X							X	X
• Other maternal outcomes			X							X	X
• Other fetal outcomes										X	X
• Unanticipated medical care											X
• Serious Adverse Events											X

APPENDIX 2. SAMPLE INFORMED CONSENT

Global Network for Women's & Children's Health Research

Aspirin Supplementation for Pregnancy Indicated Risk Reduction In Nulliparas (ASPIRIN)

INVESTIGATORS:
[LIST SITE INVESTIGATORS]

SPONSOR:
The Eunice Kennedy Shriver National Institutes of Child Health and Human Development (NICHD)

You are being asked to participate in a research study for pregnant mothers. This project is funded by the U.S. National Institutes of Health. This form provides you with information about the study. A member of the research team will describe the study to you and answer all of your questions. Please read the information below and ask questions about anything you don't understand before deciding whether or not to take part. You may also request that the research staff read the form to you.

What is the purpose of the study?
The purpose of this study is to learn whether a daily 81 mg dose of aspirin given to pregnant women, beginning between 6-13 weeks of pregnancy and continuing until 36 weeks of pregnancy, can reduce the risk of preterm birth (when a baby is born too soon).

Who will be in the study?
A total of 11920 women will be enrolled in this study from seven sites in sub-Saharan Africa, South Asia, and Latin America. In [insert site name], no more than [insert max sample size] will be enrolled. Half of the participants will be randomly selected to take aspirin. The other half will be randomly selected to take placebo, a pill that looks identical to aspirin but does not contain any medicine. This will allow the researchers to compare how well aspirin works to prevent babies being born too soon.

You qualify for this study if you are a healthy pregnant woman between 18-40 years of age, have had an ultrasound showing that your pregnancy is between 6-13 weeks, have never given birth before, have no more than 2 previous pregnancy losses, and have no known problems taking aspirin. If you qualify, you will be randomly assigned to either the treatment group, which will take a daily low dose of aspirin, or the control group, which will take a pill that looks identical to aspirin but does not have any active drug.

What will happen if I join this study?
Before participating, you will be provided with information about the study procedures and given an opportunity to ask questions. If you agree to participate, you will be asked to sign this form to indicate your consent. The research staff will perform a pregnancy test to confirm that you are pregnant and an ultrasound to make sure that the baby is in your womb and has a heartbeat. The ultrasound will also measure the size of your baby to help confirm how many weeks you have been pregnant. After the ultrasound, the research staff will ask you some questions about your health, do a brief medical exam, and conduct a hemoglobin test to make sure you are healthy enough to participate. For the hemoglobin test, a small sample of blood (2-3 drops) will be taken by pricking and gently squeezing your finger and will be used to measure the level of iron in your blood. If the iron level is too low (known as anemia),

you will not be able to participate. Instead, you will be referred to a health provider who will provide care for anemia.

After all these procedures are completed and your eligibility is confirmed, you will be randomly assigned to either the treatment group that takes a daily low dose of aspirin or the control group that takes a daily placebo pill. The aspirin and placebo look identical so there is no way for you or the research staff to know your assigned group. Both groups will receive exactly the same instructions for taking the drug and will participate in exactly the same activities throughout the study. At the time of enrollment, you will be provided with verbal instructions for safe administration and storage of the drug. You may be asked to take your first dose while you are with the research staff.

A member of the research team will set up bi-weekly visits to check on side effects and make sure you are taking the medicine properly. When needed, you will also be given a new supply of medicine. The research staff will conduct special tests at some of the bi-weekly visits to make sure you are not experiencing side effects. The special tests are:

- Blood pressure to assess for the development of pre-eclampsia at the following time points in pregnancy (1) between 16-20 weeks; (2) between 28-30 weeks; (3) at 34 weeks; (4) every 2 weeks after that until delivery. If your blood pressure is high, you will also be asked to provide a small amount of urine (about 2 tablespoons) for additional testing.
- Hemoglobin testing to assess for anemia (low iron in the blood) at 26-30 weeks pregnancy.
- The research staff will choose up to 500 women total from all sites combined who will be asked to participate in additional hemoglobin testing 4 weeks after enrollment.
- To be sure that you do not have more than the normally expected hospital or clinic visits during your pregnancy as a result of study participation, the research staff will ask you about unexpected visits and will need as much information as you can give them. They will also collect information about the reason for your unexpected visit from your clinical record.

The local research staff have been selected because of their skills, knowledge, and familiarity with your community. They will begin bi-weekly visits two weeks after enrollment and will continue these visits every two weeks until you deliver. The research staff are here to support you during the study and should be contacted between visits if you have any questions or concerns.

<u>What are the risks and discomforts</u>

Aspirin has been used for many years to safely treat adults and children. Extensive research shows that the risks of this intervention are minimal. There is a small but rare risk of allergic reactions; therefore, you will not be able to participate if you have had a bad reaction to aspirin in the past or if you have a bad reaction while in the study. Aspirin use in pregnancy has not been well-studied in developing countries with high rates of anemia, although studies of pregnant women in high-income countries do not suggest an increased risk. Nonetheless, hemoglobin levels will be monitored during the study and women who are very anemic at the time of enrollment will be excluded from the study. If you become very anemic during the study, you will be referred to your health provider for care and treatment. Additionally, pregnant women may be at risk for vaginal bleeding, but this risk has also not been supported by recent studies. A commonly reported side effect of aspirin use is gastric discomfort (an upset stomach); however, you will be given pills with a special coating which will help prevent the stomach discomfort that aspirin causes in some people. Any discomfort or side effect should be reported to your health provider and the research staff for further assessment. Based on this assessment, the research staff may decide to stop giving you the study drug.

For babies, there is limited evidence that aspirin use in pregnancy may increase the risk of gastroschisis, a birth defect of the abdominal (belly) wall. Fetal anomalies (birth defects) will be monitored throughout the study.

You may feel temporary discomfort or pain when your blood is taken with a finger stick. To minimize this, we will ensure research staff are well trained in the procedure.

Another possible risk of participating in this study is that your name and personal information may be seen by persons who are not part of the project. To prevent this, you will be given an identification number that will be used in place of your name on all study documents.

<u>What are the benefits of participating?</u>
You will not receive any money from participating in this study, but your participation may provide important information that can be used in the future to prevent babies from being born too soon. Also, there is existing evidence that the use of aspirin in pregnancy can improve the infant's neurological outcomes.

If new information about the benefits or risks of aspirin use in pregnancy becomes available during this study, this information will be given to you by [Insert name of Senior Investigator] or his/her staff.

<u>Will I have to pay for anything?</u>
It will not cost you anything to be in the study.

<u>Is my participation voluntary?</u>
Taking part in this study is voluntary. You have the right to refuse to participate or to withdraw your participation at any time. If you refuse or decide to withdraw, you will not lose any benefits or rights to which you are entitled. These actions will not have any negative effect on the health care you receive from your local health providers. You will still receive your normal medical care.

<u>Can I be removed from this study?</u>
You will be withdrawn from the study if the research staff thinks that your participation may cause you harm. The research staff may also remove you from the study for other reasons at their discretion. Also, the sponsor may stop the study at any time.

<u>What will happen if you are injured by this research?</u>
Although the risk of injury is expected to be very low, all research involves a chance that something bad might happen to you. Despite all safety measures, your participation could result in a reaction or injury. If you or your infant is injured as a result of your participation, you will be provided with emergency care by the study and referred to a doctor for ongoing care, if needed. Ongoing care will not be paid for by the study. [Insert name of Research Institution] and NICHD have not set aside funds to pay you for any such reactions, injuries or related medical care. However, by signing this form, you do not give up any of your legal rights.

<u>What should you do if you have additional questions?</u>
If you have questions about this study or a project-related injury, you should contact [investigator contact]. If you have questions about your or your baby's rights as a project participant, please contact [insert ethics committee contact].

If you have any questions about the study, please call [insert senior investigator].

Agreement to be in this study

I have read this paper about the study or it was read to me. I understand the possible risks and benefits of this study. I know that being in this study is voluntary and I choose to be in this study. I understand I will get a copy of this consent form.

Signature (or thumbprint):_______________________________________ Date:_______________
(Mother)

Print Name:____
(Mother)

Signature (or thumbprint):____ Date:_______________
(Parent/Guardian/Husband)

Print Name:____
(Parent/Guardian/Husband)

APPENDIX 3. OUTCOME DEFINITIONS

PRIMARY OUTCOME:

- ***Preterm Birth:*** delivery at or after 20 0/7 weeks and prior to 37 0/7 weeks.
  - WHO definition: babies born before 37 completed weeks of pregnancy.
  - ACOG definition: Birth occurs between 20 weeks and 37 weeks of pregnancy (http://www.acog.org/~/media/For%20Patients/faq087.pdf)

SECONDARY OUTCOMES:

- ***Preeclampsia:*** characterized by hypertension and proteinuria occurring after the 20th week of pregnancy. Hypertension is a blood pressure of 140/90 mm Hg or above. In severe pre-eclampsia the diastolic blood pressure is usually 110 mm Hg or above and there may also be one or more of the following symptoms: severe headache, blurred vision, nausea and/or vomiting, abdominal pain and a diminished urinary output, i.e., oliguria. ***Early onset Preeclampsia*** develops before 34 weeks of gestation.

- ***Eclampsia:*** Very serious complication of pregnancy characterized by convulsions and coma. It may be preceded by signs of pre-eclampsia or the onset may be rapid and sudden. Eclamptic fits can occur in pregnancy, labour or soon after delivery.

- ***Small for gestational age:*** newborns with weights below the 10th percentile for gestational age.

- ***Perinatal mortality:*** The number of stillbirths and deaths in the first week of life per 1,000 live births, the perinatal period commences at 22 completed weeks (154 days) of gestation and ends seven completed days after birth.

MATERNAL OUTCOMES OF INTEREST

- ***Vaginal bleeding:*** Bleeding during pregnancy (i.e., WHO generally defines as 'light' or 'heavy' bleeding based on clinical symptoms)

- ***Antepartum hemorrhage:*** Bleeding from the genital tract at any time after the 22nd week of pregnancy and before the birth of the baby. There are two main causes of antepartum haemorrhage, placenta praevia and abruption placentae.

- ***Postpartum hemorrhage:*** Blood loss of 1000 ml or more from the genital tract after delivery and up to six weeks post-delivery. Common causes include atony (poor muscle tone) of the uterus or trauma to the genital tract, e.g. tears of the vagina, cervix, or lower segment of the uterus.

- ***Maternal mortality:*** the death of a woman during pregnancy (i.e. conception to delivery) and the puerperium (i.e. up to 42 days after delivery).

- ***Change in maternal hemoglobin:*** Hemoglobin level <7.0 gm/dL or a 3.5 gm/dL decrease between measurements.

FETAL OUTCOMES OF INTEREST

- ***Preterm birth <34 0/7 weeks of pregnancy:*** live birth before 34 0/7 weeks of pregnancy are completed.

- ***Birth weight <2500g and Birth weight <1500g:*** Birth weight is defined as the first weight of the fetus or newborn obtained after birth. For live births, birth weight should preferably be measured within the first hour of life before significant postnatal weight loss has occurred, recorded to the degree of accuracy to which it is measured (WHO). Birth weight <2500 g is defined as low birth weight, whereas birth weight <1500 g is defined as very low birth weight.

- ***Fetal Loss:*** Spontaneous loss ≥ 16 weeks GA plus perinatal mortality.

- ***Late abortion:*** Spontaneous fetal loss ≥ 16 weeks.

- ***Spontaneous abortion:*** Premature expulsion of a non-viable fetus from the uterus at < 20 weeks gestation.

- ***Stillbirth:*** Birth of a baby that shows no signs of life at birth (no gasping, breathing, heart beat or movement). (WHO). It may be a macerated or fresh stillbirth. The signs of maceration are discoloration and peeling of the skin leaving areas of raw tissue. The skull is usually soft, as the brain has become soft. The umbilical cord is usually stained a dark red or black. The amniotic fluid is usually darkly stained. Maceration is the result of the infant being dead for at least 12 hours. Macerated stillborn infants are assumed to have died before the onset of labor. Fresh stillbirths show no sign of maceration and have usually died during labor or shortly before the onset of labor.

- ***Medical termination of pregnancy:*** an operation or other procedure to terminate pregnancy before the fetus is viable.

NOTE: (WHO) at the end of the definition means the definition is from the 2006 WHO guide titled: *Integrated Management of Pregnancy and Childbirth Pregnancy, Childbirth, Postpartum and Newborn Care: A guide for essential practice*.

APPENDIX 4. STUDY TIMELINE

	YEAR 1				YEAR 2				YEAR 3			
	Q1	Q2	Q3	Q4	Q1	Q2	Q3	Q4	Q1	Q2	Q3	Q4
**Preparatory Activities**												
▪ Document Development (Protocol, forms, etc.)												
▪ Procurement of drug and other materials												
▪ Approvals (IRB, ERC, Drug Authorities, etc.)												
▪ Site Preparation												
▪ Training												
**Recruitment**												
**Data Cleaning and Analysis**												

Appendix 5. Aspirin Protocol Steering Committee and Technical Advisors

Central Study Team: Global Network Site 08 (Belgaum, India)

Richard Derman, MD, MPH
Principal Investigator
Thomas Jefferson University
Philadelphia, PA
Richard.Derman@jefferson.edu

Bhalchandra Kodkany, MD, MBBS
Senior Foreign Investigator
KLE University's J N Medical College
Belgaum, India
drkodkany@jnmc.edu

Shivaprasad S. Goudar MD, MHPE
Co-Investigator
KLE University's J N Medical College
Belgaum, India
sgoudar@jnmc.edu

Matthew K. Hoffman, MD, MPH
Lead Investigator for Aspirin Protocol
Christiana Care
Newark, Delaware
Mhoffman@christianacare.org

Mrityunjay C. Metgud, MD
Aspirin Protocol Country Coordinator,
KLE University's J N Medical College
Belgaum, India
metm67@gmail.com

Frances Jaeger, MA, DrPh
Co-Investigator
Thomas Jefferson University
Philadelphia, PA
Frances.Jaeger@jefferson.edu

National Institute of Child Health and Human Development (NICHD)

Marion Koso-Thomas, MD, MPH
Medical Officer, Global Network for Women's and Children's Health
Eunice Kennedy Shriver National Institute of Child Health and Human Development (NICHD)
kosomari@mail.nih.gov

Menachem Miodovnik, MD
Director – Global Network for Women's and Children's Health
Eunice Kennedy Shriver National Institute of Child Health and Human Development (NICHD)

menachem.miodovnik@nih.gov

RTI International

Elizabeth McClure, PhD
Principal Investigator, Data Coordinating Center
mcclure@rti.org

Dennis Wallace, PhD
Co-Principal Investigator, Data Coordinating Center
Senior Statistician
dwallace@rti.org

Jay Hemingway-Foday, MPH, MSW
Protocol Manager for Aspirin Protocol
hemingway@rti.org

Site Investigators

Global Network Site 02 (Democratic Republic of Congo)

Carl Bose, M.D. .
Principal Investigator
University of North Carolina School of Medicine
Chapel Hill, North Carolina
cbose@med.unc.edu

Antoinette Tshefu, M.D, Ph.D., M.P.H
Senior Foreign Investigator
Kinshasa School of Public Health
antotshe@yahoo.com

Adrien Lokangaka, M.D., M.P.H.
Aspirin Protocol Country Coordinator
Kinshasa School of Public Health
adrinloks@yahoo.fr

Global Network Site 03 (Zambia)

Wally Carlo, MD
Principal Investigator
University of Alabama at Birmingham
Birmingham, Alabama
Wcarlo@PEDS.UAB.EDU

Elwyn Chomba, MBChB, DCH, MRCP
Senior Foreign Investigator
University Teaching Hospital
Lusaka, Zambia
echomba@zamnet.zm

Cindy Chirwa, MD
Aspirin Protocol Country Coordinator
Lusaka, Zambia

Cindychirwa78@gmail.com

Global Network Site 06 (Guatemala)

Michael Hambidge, MD
Principal Investigator
University of Colorado Health Care System (UCHSC)
Denver, Colorado
Michael.hambidge@ucdenver.edu

Nancy Krebs, MD
Co-Investigator
UCHSC
Denver, Colorado
Nancy.krebs@ucdenver.edu

Ana Garces, MD, MPH
Senior Foreign Investigator
FANCAP
Guatemala City, Guatemala
agarces@fancap.org

Lester Figueroa, MD
Aspirin Protocol Country Coordinator
FANCAP
Guatemala City, Guatemala
lesterfigueroa@me.com

Global Network Site 09 (Pakistan)

Robert Goldenberg, MD
Principal Investigator
Columbia University
New York, New York
rlg88@columbia.edu

Sarah Saleem, MD
Senior Foreign Investigator
Aga Khan University
Karachi, Pakistan
Sarah.saleem@aku.edu

Global Network Site 11 (Nagpur, India)

Patricia L. Hibberd, MD, PhD
Principal Investigator
Boston University
Boston, Massachusetts
plh0@bu.edu

Archana Patel, MD, DNB, MSCE
Senior Foreign Investigator
Indira Gandhi Government Medical College
Nagpur, India
Dr_apatel@yahoo.com

Prabir B. Das, MD
Aspirin Protocol Country Coordinator
Lata Medical Research Foundation (LMRF)
prabir_das23@rediffmail.com

Global Network Site 12 (Kenya)

Ed Liechty, MD
Principal Investigator
Indiana University School of Medicine
Indianapolis, Indiana
eliecht@iu.edu

Fabian Esamai, MBChB, MMed, PhD
Senior Foreign Investigator
Moi University School of Medicine
Eldoret, Kenya
fesamai2007@gmail.com

Technical Advisory Group

Robert M. Silver, MD
University of Utah Health Sciences Center
bob.silver@hsc.utah.edu

Enrique Schisterman, Ph.D
Chief and Senior Investigator
Epidemiology Branch, DIPHR
Eunice Kennedy Shriver National Institute of Child Health and Human Development
schistee@mail.nih.gov

STATISTICAL ANALYSIS PLAN

Aspirin Supplementation for Pregnancy Indicated Risk Reduction In Nulliparas (ASPIRIN)

The ASPIRIN Study

SAP VERSION: Version 3

SAP DATE: March 18, 2019

SPONSOR: NICHD

PREPARED BY: RTI International

3040 Cornwallis Rd

Research Triangle Park, NC 27709-2104

AUTHOR (S): Dennis Wallace, Beth McClure, Tracy Nolen

Contents

1. BACKGROUND AND PROTOCOL HISTORY 6

2. PURPOSE OF THE ANALYSES 6

3. STUDY OBJECTIVES AND OUTCOMES 7

3.1 PRIMARY HYPOTHESIS AND ASSOCIATED OUTCOMES 7

3.2 SECONDARY HYPOTHESES AND ASSOCIATED OUTCOMES 7

3.3 OTHER OUTCOMES OF INTEREST IN THIS STUDY 8

4. STUDY METHODS 8

4.1 OVERALL STUDY DESIGN 8

4.2 STUDY POPULATION 10

4.3 STUDY ARM ASSIGNMENT AND RANDOMIZATION 11

4.4 MASKING AND DATA LOCK 11

5. ANALYSIS POPULATIONS 12

6. SAMPLE SIZE DETERMINATION 13

6.1 PRIMARY OUTCOME 13

6.2 SECONDARY OUTCOMES 13

7. STATISTICAL / ANALYTICAL ISSUES 14

7.1 GENERAL RULES 14

7.2 ADJUSTMENTS FOR COVARIATES 15

7.3 MISSING DATA APPROACHES: 15

7.4 MULTICENTER STUDIES 15

7.5 MULTIPLE COMPARISONS AND MULTIPLICITY 16

7.6 MASKED DATA REVIEW 16

8. STUDY PARTICIPANTS, TREATMENT EXPOSURE AND COMPLIANCE 16

8.1 PARTICIPANT DISPOSITION 16

8.2 STUDY TREATMENT EXPOSURE AND COMPLIANCE 16
8.3 PROTOCOL DEVIATIONS 17
8.4 DEMOGRAPHIC AND BASELINE CHARACTERISTICS 17
9. EFFICACY ANALYSES 17
9.1 OVERVIEW OF EFFICACY ANALYSIS METHODS 17
9.2 DEFINITION OF ANALYSIS VARIABLES 17
9.4 PRIMARY ANALYSIS 26
9.5 SECONDARY OUTCOME ANALYSES 26
9.6 ANALYSES OF OTHER OUTCOMES OF INTEREST 27
10. SAFETY ANALYSIS: 27
11. INTERIM ANALYSES AND DATA MONITORING 27
12. CHANGES TO THE ANALYSES PLANNED IN THE PROTOCOL 28
13. REFERENCES 28
14. ATTACHMENTS 29

LIST OF ABBREVIATIONS

Acronym	Definition
DCC	Data Coordinating Center
DMC	Data Monitoring Committee
DRC	Democratic Republic of the Congo
EmONC	Emergency Obstetric and Neonatal Care
GA	Gestational Age
GEE	Generalized Estimating Equation
GN	Global Network
ITT	Intention to Treat
LMP	Last menstrual period
MDG	Millennium Development Goals
mITT	Modified intention to treat
MNH	Maternal and Neonatal Health
PP	Per protocol
RCT	Randomized Clinical Trial
SAP	Statistical Analysis Plan
UN	United Nations

1. BACKGROUND AND PROTOCOL HISTORY

Background and Rationale

Preterm delivery, defined as delivery prior to 37 weeks 0/7 days gestation, remains the dominant cause of neonatal morbidity and mortality throughout the world, (March of Dimes, 2012, Matthews, 2004, Anderson, 2003) and directly leads to 28% of neonatal deaths within the first seven days of life. (Beck, 2010) Moreover, it is responsible of up to 50% of pediatric neurodevelopmental disorders. (Goldenberg, 1998) Infants born prematurely are likewise at increased risk for a variety of long-term medical complications such as respiratory, gastrointestinal, cardiovascular, and metabolic disorders. (McCormack, 2002, Saigal, 2008)

Compounding the issue, the risk of preterm birth is highest in developing countries where an estimated 12% of births are preterm compared to 5-7% in developed countries. (Valero De Bernabé, 2004) Of the near 13 million preterm births worldwide in 2005, 11 million were in Africa and Asia. (Blencowe, 2010) Given the tremendous medical, financial and emotional burden of preterm birth in the developing world and the limited resources to provide postnatal care, any interventions with the potential to reduce the risk of preterm birth deserve consideration. If one were to choose an intervention, it would be one that is widely available, inexpensive, and safe for the mother and fetus. Low dose aspirin may be just such an intervention.

We intend to study the effects of LDA in nulliparous women for several reasons. First, although it would be of interest to study the effect of LDA in women at high risk for preterm birth (e.g. prior preterm birth), such women may undergo interventions intended to decrease their risk of preterm birth. Thus, a study in patients with prior preterm birth would have numerous potential confounders. Conversely, multiparous women with prior term births would be at very low risk for preterm birth and also be a suboptimal population to study. Nulliparous women appear to be an ideal population since they will not undergo special interventions in an attempt to avoid preterm birth. Also, it appears that the risk of preterm birth in nulliparous women is higher than for the general obstetric population. In summary, available data suggest that LDA may be a safe, widely available and inexpensive intervention that may significantly reduce the risk of preterm birth. However, this possibility needs to be proven in a properly designed RCT with preterm birth as the primary outcome. Such a clinical trial in a racially, ethnically and geographically diverse population could best be accomplished through the established infrastructure of the Global Network for Women's and Children's Health Research (Global Network or GN).

Protocol History

The protocol was initiated in March 2016 under Protocol version 1.1. Protocol amendment 1.2 was generated in December 2016.

This study completed randomization during the summer of 2018, with follow-up completed approximately 9 months after the last participant is randomized. No formal interim analyses were planned nor completed for this study and as such, the first formal assessment for efficacy is planned for after completion of primary follow-up.

2. PURPOSE OF THE ANALYSES

This statistical analysis plan (SAP) contains detailed information about statistical analyses to be performed to assess the primary and secondary hypotheses outlined in the protocol (i.e. to evaluate

whether women who are treated with LDA daily beginning between 6 0/7 weeks and 13 6/7 weeks GA through 36 0/7 weeks GA will have a reduced risk of preterm birth from all causes, and whether these women will have reduced risk of small for gestational age (SGA) deliveries, reduced risk of eclampsia and preeclampsia during pregnancy, and reduced risk of infants who experience perinatal mortality. The results of these analyses will be included in the study manuscript(s). Additional exploratory analyses may be performed to support further manuscript development. These analyses will not require an update to the SAP, but abbreviated analysis plans will be prepared prior to conducting those analyses.

3. STUDY OBJECTIVES AND OUTCOMES

3.1 Primary Hypothesis and Associated Outcomes

Our primary hypothesis is that nulliparous women with no more than two previous first trimester pregnancy losses who are treated with LDA daily beginning between 6 0/7 weeks and 13 6/7 weeks GA through 36 0/7 weeks GA will reduce the risk of preterm birth from all causes.

The primary outcome of this study is preterm birth, which will be defined as delivery at or after 20 0/7 weeks and prior to 37 0/7 weeks. This will be determined based on actual date of delivery in comparison to the projected EDD, independent of whether or not the preterm delivery is indicated or spontaneous.

3.2 Secondary Hypotheses and Associated Outcomes

The study will also test three secondary hypotheses:

- Infants of women who take antenatal daily LDA initiated at 6 0/7 to 13 6/7 weeks GA will be at lower risk of being small for gestational age (SGA).
- Women who take antenatal daily LDA initiated at 6 0/7 to 13 6/7 weeks GA will have lower risk of: eclampsia and preeclampsia. Note that this hypothesis will be operationalized as evidence of hypertensive disorders of pregnancy as defined in more detail in Sections 9 and 12.
- Infants of women who take antenatal daily LDA initiated at 6 0/7 to 13 6/7 weeks GA will be at lower risk of perinatal mortality.

These secondary hypotheses will be tested using the following outcomes.

- Small for gestational age infants include newborns with weights below the 10th percentile for gestational age, as defined by Intergrowth Standards.

- Risk of Pregnancy Induced Hypertension (eclampsia and/or preeclampsia) defined as a binary outcome of any of new onset hypertension that occurs beyond 20 weeks gestational age, preeclampsia, or eclampsia happening between initiation of study dosing and participant completion of the study at 6 weeks after delivery. Eclampsia is a serious complication of pregnancy characterized by convulsions and coma. It may be preceded by signs of pre-eclampsia or the onset may be rapid and sudden and can occur in pregnancy, labor or soon after delivery. Preeclampsia is characterized by hypertension and proteinuria occurring after the 20th week of pregnancy. See Sections 9 and 12 for more detail.

- Perinatal mortality is a binary outcome that includes any stillbirth occurring after 20 weeks pregnancy and any early neonatal death that occurs within 7 days after delivery.

3.3 Other Outcomes of Interest in this Study

Study analyses will examine a number of additional maternal and fetal or neonatal outcomes to assess whether use of LDA daily beginning between 6 0/7 weeks and 13 6/7 weeks has an effect on those outcomes. Specific maternal and fetal/neonatal outcomes include:

Maternal outcomes:

- Vaginal bleeding
- Antepartum hemorrhage
- Postpartum hemorrhage
- Maternal mortality
- Late abortion
- Preterm, preeclampsia

***Fetal/Neonatal* outcomes**:

- Preterm birth <34 0/7 weeks of pregnancy
- Birth weight <2500g and <1500g
- Fetal loss
- Spontaneous abortion
- Stillbirth
- Medical termination of pregnancy

Detailed definitions of these outcomes are included in Section 9. Change in maternal hemoglobin is an other/exploratory outcome collected for this study. Analyses of these data will be performed for a secondary manuscript and are not covered under this analysis plan.

4. STUDY METHODS

4.1 Overall Study Design

The ASPIRIN trial was conducted as a randomized, placebo-controlled, double-blinded multicenter clinical trial to assess the efficacy of LDA in the reduction of preterm birth. Women were randomized equally to receive either daily LDA (81 mg) or an identical appearing placebo beginning between 6 0/7 weeks and 13 6/7 weeks GA and continuing until 36 0/7 weeks GA or delivery.

The study intervention is 81 mg of aspirin administered daily beginning between 6 0/7 weeks and 13 6/7 weeks through 36 0/7 weeks or delivery. Following randomization, each woman was to be provided with a supply of either LDA or an identical appearing placebo. The drug or placebo were enteric coated pills, provided in identical, child-resistant packaging, with written instructions for use. To ensure that literacy levels did not affect proper use, the study staff was to provide verbal instructions at randomization and reinforce these guidelines at subsequent follow-up visits. Each woman was to also receive a back-up supply of medication or placebo, which will be maintained throughout the duration of the study. The purpose of the backup is to bridge circumstances wherein the woman misses or is late to a planned follow-up visit or the primary supply of study drug/placebo is misplaced or destroyed. Medication compliance was to be monitored, and the drug/placebo supply was to be replenished during routine study visits.

A study schematic is shown below

Study Schematic

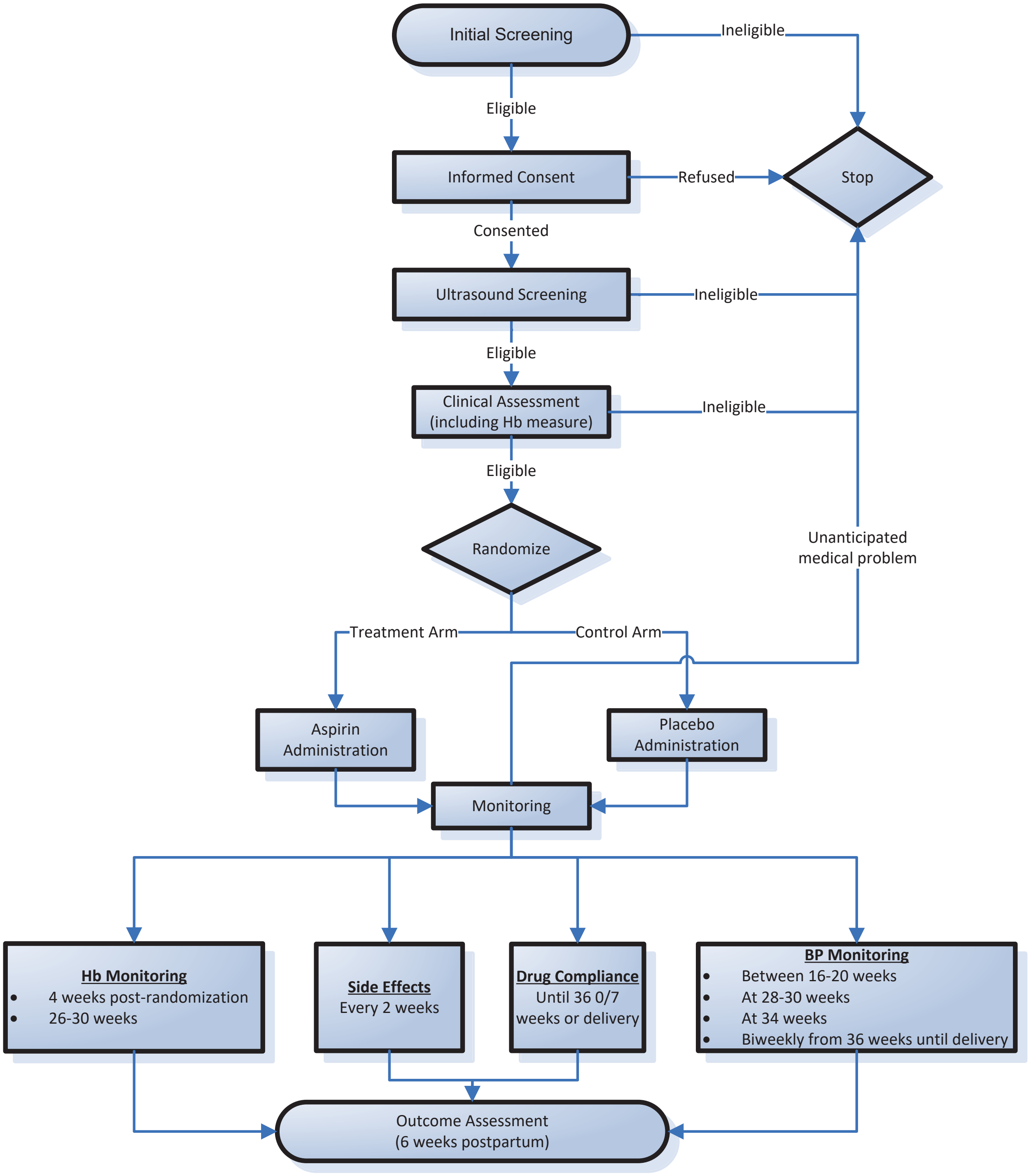

4.2 Study Population

Studying the effect of LDA in women at very high risk for preterm birth (e.g., prior preterm birth) would have numerous confounders, as these women are likely to undergo interventions intended to

decrease their risk. Conversely, multiparous women with prior term births would be at very low risk for preterm birth, making them a suboptimal study population. Nulliparous women appear to be the ideal population to study the effects of LDA since they will not undergo special interventions to avoid preterm birth. Multiple studies have shown that the risk of preterm birth in nulliparous women is higher than for the general obstetric population.

Inclusion criteria

- Nulliparous women between 18 – 40 years of age. Minors who are ≥ 14 years of age may be enrolled if permitted by the country's ethical guidelines.
- No more than two previous first trimester pregnancy losses
- No medical contraindications to aspirin;
- Single live intrauterine pregnancy (IUP) between 6 0/7 and 13 6/7 weeks GA corroborated by an early dating ultrasound and with a fetal heart rate detected.

Exclusion criteria

- Women prescribed daily aspirin for more than 7 days;
- Multiple gestations;
- Fetal anomaly by ultrasound (Note most fetal anomalies are not detectable by ultrasounds done at this early gestation. Subsequent discovery of a fetal anomaly is not viewed as an exclusion.);
- Hemoglobin < 7.0 g/dl at screening;
- Systolic blood pressure ≥ 140 and diastolic ≥ 90 at screening;
- Any other medical conditions that may be considered a contraindication per the judgment of the site investigator (e.g., Lupus, Type 1 Diabetes, or any other known significant disease)

4.3 Study Arm Assignment and Randomization

Randomization of subjects was carried out to obtain a 1:1 allocation ratio between the treatment and placebo arms. Randomization was stratified by site. A computer algorithm generated by the data coordinating center (DCC) was created the random assignment to one of the treatment arms based on randomly permuted block design with randomly varied block sizes. The block sizes were known only by the DCC personnel. A total of 11,920 nulliparous women were to be enrolled (5960 per group) across seven sites in sub-Saharan Africa, South Asia, and Latin America. For balance, each site was to enroll no more than 25% of the total sample.

To study the possible effect of LDA on anemia, an ancillary study was to enroll at least 500 women to obtain hemoglobin measure at 4 weeks after initiation of therapy, with each site contributing at least 75 women to that sample. Some sites ultimately collected hemoglobin 4-weeks post randomization for all participants. In addition, hemoglobin measures were to be obtained from all women at 26 to 30 weeks gestational age. The analyses of this ancillary study will be described in a separate analysis plan document and will not be included as part of the primary study publication.

4.4 Masking and Data Lock

Both the formulation of the medication and placebo were procured from two manufacturers, with one manufacturer supplying materials for Pakistan participants and the other manufacturer supplying

materials for the participants at the other sites. The packaging was standardized across sites and labeled as ASA 81 mg/placebo, with the expiration data and a unique identifier. A certificate of authenticity was to likewise be provided. Manufacturing details are maintained in the study file.

Throughout the study, research staff and local health providers were masked to treatment status unless there was a serious adverse event potentially related to the treatment modality that requires unmasking for safety reasons. There was one pharmacist at each site who was to remain unmasked to monitor randomization, drug supply, and safety as needed.

Data Lock: Data will be locked at the site level with final analysis data sets generated and locked once data from all sites have been received and all queries processed. For each site, data collection for the protocol will stop approximately 9 months after the last pregnant woman was randomized into the study at that site. Site will transmit all data forms to the site within three months of this date, and data will be locked three to six months post last follow-up visit. Note that other than for the cases on individual unmasking described above, which are anticipated to be minimal, the study will not be unmasked until after data lock.

5. ANALYSIS POPULATIONS

Safety (SAF) Population

The safety population will comprise all randomized participants who received any study treatment grouped by actual treatment received, irrespective of amount or duration of treatment received. Unless specified otherwise, data for this population will be analyzed as available (i.e. irrespective of protocol deviation occurrence, subsequent study participation termination, or study drug withdrawal).

Modified Intention to Treat (mITT) Population

The primary analysis population will be the modified intention to treat population, which includes all eligible, randomized participants who provide any post-baseline outcome data and who deliver at 20 weeks gestational age or greater. All participants will be assigned to the arm to which they were randomized irrespective of treatment received. Unless specified otherwise, data for this population will be analyzed as available (i.e. irrespective of protocol deviation occurrence, subsequent study participation termination, or study drug withdrawal). This population will also be used for many of the secondary analyses. Note that this population represents a change from the protocol, which indicates that the ITT population will be used for the primary analyses. This change was made prior to any unmasking.

Intention to Treat (ITT) Population

The intention to treat population will include all eligible, randomized participants. All participants will be assigned to the arm to which they were randomized irrespective of treatment received. Unless specified otherwise, all participants will be included in analyses using this population (with data imputed as needed and irrespective of protocol deviation occurrence, subsequent study participation termination, or study drug withdrawal). This population will be used to conduct sensitivity analyses for the primary outcome.

Per Protocol (PP) Population

This population will be used to conduct sensitivity analyses for the primary outcome. This population will exclude all or part of the data obtained from any eligible, randomized participants who deliver at 20 weeks gestational age or greater that did not receive at least 90% of the full amount of intended randomized study therapy or are considered to have substantially deviated from the protocol in a manner that may impact study outcome or treatment receipt. The population will also exclude individuals who were randomized after 10 weeks, 6 days gestational age. Participants will be grouped by actual treatment received. Treatment receipt reasons and substantial deviations that will lead to exclusion include:

- Documented receipt of aspirin while on study outside of assigned study drug as identified by a reported protocol deviation
- Not receiving treatment after randomization: All data from these participants will be excluded.
- Receipt of less than 90% of planned total doses after randomization where the number of planned doses will be calculated as those expected between randomization and the earliest of end of pregnancy and 36 0/7 weeks GA irrespective of if study drug administration was prematurely discontinued for any reason prior to that point: All data from these participants will be excluded.

Receiving more than the planned total doses of study drug will generally NOT be an exclusion criterion for the PP population. Additional exclusion reasons may be identified after completion of participant enrollment. Any participant deviations that will exclude participants or their data from the PP analyses will be identified by the study team, prior to review of efficacy and safety data, as a part of a masked data review to reduce the opportunity for introducing of bias and will be documented in an addendum to this analysis plan.

6. SAMPLE SIZE DETERMINATION

6.1 Primary Outcome

The risk of preterm birth < 37 weeks gestation in untreated nulliparous women was estimated to be about 8.0% for the EAGeR trial (Abramovitch, 2012 and Schusterman, 2014). Unpublished data for 2012 from the GN sites shows preterm birth rates, ranging from 2.9% to 9.8%, while WHO estimates the range for the countries in which GN sites are located to be 7.7% to 16.7% 1. In developing sample size estimates we considered risks in the range of 8% to 14% and ultimately selected sample sizes based on a conservative estimate of 8%. Because the association of LDA and preterm birth may differ in international settings, we examined sample size requirements for reductions of 40%, 25%, and 20%. As shown in the protocol, the number of evaluable participants needed to detect a 20% reduction from a usual rate of 8% preterm births with 90% power is 5,483 per treatment arm. To account for the loss of evaluable subjects due to miscarriages, which are anticipated to occur in approximately 5% of participants) and loss to follow-up (assumed to be in the 1% to 2% range), sample sizes were increased by 8% to obtain a final sample size of 11,920 (or 5,960 per treatment arm).

6.2 Secondary Outcomes

One of the key secondary objectives of this trial is to evaluate the effect of low dose aspirin on perinatal mortality. We evaluated the reduction in perinatal mortality that could be detected with 80% power and 90% power under the assumption that the sample size for the study would be based

on that needed to achieve the primary aim of demonstrating a 20% reduction in low-birth weight infants. The analyses were based on the assumption that the perinatal mortality rate in the GN population is approximately 45 perinatal deaths per 1000 deliveries (unpublished data from the MNH Registry for 2013). With this estimated underlying mortality rate and 5,483 evaluable subjects per treatment arm required to demonstrate the 20% reduction in low birth weight prevalence as outlined in the primary aim, the study will have 80% power to detect a 24% reduction in perinatal mortality and 90% power to detect a 27% reduction in perinatal mortality.

To determine the sample size actually needed to demonstrate the reduction in perinatal mortality actually expected if the intervention results in a 20% reduction in prevalence of low birth weight infants, we examined both the prevalence of low birth weight infants across the GN sites and the perinatal mortality risk in both the low birth weight and normal birth weight infants. Based on 2013 MNH Registry data, the prevalence of low birth weight among all GN deliveries was 16.3% and the perinatal mortality rates were 20.5 per 1000 deliveries among normal birth weight infants and 162.3 per 1000 deliveries among low birth weight infants; these values yield a weighted average perinatal mortality rate of 43.6 per 1000 deliveries. If the low dose aspirin intervention is effective in reducing the prevalence of low birth weight deliveries by 20% (i.e. reducing the prevalence from 16.3% to 13%) and the conditional risk of perinatal mortality is unchanged in the low birth weight and normal birth weight cohorts, we would expect the resultant overall perinatal mortality rate to be reduced to 38.9 per 1000 deliveries or a 10.8% reduction. Detection of a reduction of this magnitude with 80% power would require an evaluable sample size of greater than 28,000 evaluable deliveries per treatment arm, and the study would need over 37,000 evaluable deliveries per arm to achieve 90% power to detect a reduction of this level in perinatal mortality.

Given the sample sizes associated with the second approach, the best alternative appears to be to power the study to demonstrate the 20% reduction in prevalence of low birth weight deliveries with a commitment to generate point and interval estimates of the impact of the intervention on perinatal mortality. By examining the risk of perinatal mortality in both the normal and low birth weight infants in the two arms, the trial would provide some insight about impacts of the intervention on mortality other than that mediated through the effect on birth weight.

We also conducted power analyses to examine the effect sizes that could be detected with the planned sample sizes for two additional secondary outcomes, prevalence of SGA infants and incidence of eclampsia/preeclampsia in the target population. Generic information as well as preliminary information from the 2013 MNH Registry indicates that the risk of each of these events in the population of interest is approximately 5%. Under the assumption of 5483 evaluable participants in each treatment arm and an assumed Type I error rate of 0.05 for per-comparison analyses (i.e. no control for multiple comparisons in the analyses of these secondary outcomes), the study will have 70% power to detect a 20% reduction in the risk of each of these outcome measures, 85% power to detect a 24% reduction and 90% power to detect a 26% reduction. Consequently, the study is reasonably powered to examine these secondary outcome measures.

7. STATISTICAL / ANALYTICAL ISSUES

7.1 General Rules

All statistical computations will be performed and data summaries will be created using SAS 9.3 or higher. If additional statistical packages are required, these will be discussed in the study report. For summaries of study data, categorical measures will be summarized in tables listing the frequency and the percentage of participants in each study arm; continuous data will be summarized by presenting

mean, standard deviation, median, minimum, and maximum; and ordinal data will be summarized by only presenting median and the limits of the interquartile range.

7.2 Adjustments for Covariates

As this is a randomized trial and the randomization process was not compromised during execution, the treatment effect estimate from trial is unbiased, even without adjusting for any baseline covariates. Therefore, no adjustments for covariates other than site are planned for the primary analysis. However, baseline clinical and demographic variables that are known predictors of neonatal mortality and maternal outcomes will be included in secondary analyses of intervention efficacy.

7.3 Missing Data Approaches:

The study is likely to have two primary sources of missing data. Since the primary interest is in delivery outcomes for women who deliver after 20 weeks, the first primary source for missing data is early pregnancy losses due to miscarriage or medical termination of pregnancy (MTP) prior to 20 weeks gestational age. The second primary reason for missing data will be participant loss to follow-up. Each of these scenarios is treated separately below.

Women are being enrolled in the ASPIRIN trial when they are between 6 0/7 weeks and 13 6/7 weeks gestational age. Depending on the distribution of study participants over this gestational age range we anticipate that between 2% and 8% of the women in the study will have a pregnancy loss prior to 20 weeks. For the primary outcome of preterm birth, these women will be excluded from the analyses and hence be considered missing at random. However, because we recognize that these women may not have the same risk of preterm delivery as those followed past 20 weeks, we will generate two additional sensitivity analyses to assess whether inference is robust to this assumption. For the first analyses, these early terminations will be treated as preterm deliveries in a full ITT analysis. For the second approach, the mITT population will be used with inverse probability weighting used to account for the individuals lost prior to 20 weeks.

Based on the historical data available from the MNH registry, we anticipate that the rate of missing responses for maternal and neonatal morbidity and mortality measures are likely to be 2% or less. Although we do not expect this rate to change over the course of this trial, we will examine data from both arms to ensure that the missing rate is comparable in the participants assigned to the Aspirin and Placebo treatment arms. We will examine patterns of missing data by arm and site. If more than 2% of data in either or both arms are missing, we will conduct sensitivity analysis on the effect of the missing on the inference by using use multiple imputation procedures. Multiple imputation assumes that the mechanism that caused the missing is independent of the value of the unobserved data (i.e. data are missing at random – MAR). Although there is no reason to believe that the missing data on the use would not be MAR, multiple imputation processes have been shown to be robust even if the missing mechanism is dependent on the values of the unobserved data, i.e. even is the missing data are non-ignorable non-response. The results of all sensitivity analyses will be presented in the final report.

7.4 Multicenter Studies

For this multicenter study, randomization of study participants was stratified within site. Consequently, for all test-based analyses of treatment effect and model-based primary and secondary analyses, site will be included as a fixed effect in the models. As an ancillary analysis associated with the primary outcome (risk of premature delivery) as well as secondary outcomes, we will examine

descriptively whether the treatment effects vary across sites; however, these analyses of site differences in treatment effect are considered to be descriptive because sample sizes are likely inadequate to provide adequate power to detect site by treatment interaction effects.

7.5 Multiple Comparisons and Multiplicity

There is only one formal hypothesis test for this study. As such, a statistical test will be conducted at a 5% type I error rate (two-sided) for the primary efficacy measure, and no adjustments for multiplicity will be made. All analyses of secondary outcomes are exploratory in nature; therefore, p-values and confidence intervals are provided for descriptive purposes only. All p-values provided for any baseline and demographic characteristics and safety parameters will be for descriptive purposes only. As such, unless otherwise specified, p-values presented will be on a per analysis basis, with no further control for multiple tests.

7.6 Masked Data Review

A masked data review process will be performed by members of the protocol team prior to final data lock to define study analysis populations and to adjudicate key outcome variables. Key questions that will be addressed during this review will be definition of individuals to be included in the per protocol population, adjudication of the primary outcome variable and selected secondary outcomes for this study. Detailed documentation of the masked data review and resulting decisions will be described in a SAP addendum.

8. STUDY PARTICIPANTS, TREATMENT EXPOSURE AND COMPLIANCE

8.1 Participant Disposition

Participant eligibility status will be summarized by study arm and overall disposition of study participants will be described using a standard CONSORT diagram. The number of participants randomized; completing or discontinuing from study therapy; and completing the study follow-up will be summarized by study arm. Reasons for study withdrawal will also be summarized by treatment arm to assess whether they are balanced across treatment arm and to assure that any imbalances are unlikely to affect inference.

8.2 Study Treatment Exposure and Compliance

Each participant will take daily LDA or placebo from the time of randomization until 36 0/7 weeks GA or delivery. Drug compliance will be routinely monitored throughout this period through pill counts and the completion of a medicine compliance form each time the pill supply is replenished. An interval medical obstetrical history will also be taken during these scheduled visits. Study staff will also provide reminders about proper drug administration and the importance of compliance during bi-weekly visits to monitor side effects.

- As a part of the analyses, characteristics of study drug exposure will be summarized by study arm. Characteristics that will be included in this analysis summary include
  - Gestational age at initiation of first dose treated as a binary measure before and after 10 weeks.
  - Gestational age when last dose was taken
  - Number of individuals with dose withheld under clinical direction
  - Percentage of possible days on which dose was taken:

    - As a measure of adherence where the number of planned/possible doses excludes any periods where the participants was asked by a health provider or study staff to stop taking the study medication as a result of a medication side effect or unexpected medical event
    - As a measure of exposure where the number of planned/possible doses includes all days between randomization and earliest of end of pregnancy and 36 0/7 weeks GA
  - Summarize the percent of doses missed for planned doses prior to 16 weeks GA vs. after 16 weeks GA

8.3 Protocol Deviations

Protocol deviations will be summarized by treatment arm and by site with information such as type of deviation, time of occurrence, and reason. Incidence rate of protocol deviations will also be summarized overall and for each protocol deviation category by treatment arm. Incidence rate of protocol deviations will be calculated as: number of deviations divided by the number of participant weeks at the site. The comments section will be reviewed to examine overarching themes, and these will be included in the final report.

8.4 Demographic and Baseline Characteristics

Demographic and baseline clinical characteristics for the study participants will be summarized by treatment group using the general analysis rules describe above for the mITT, ITT, PP and SAF populations. Variables of interest include maternal age, maternal education, gravidity, maternal weight and BMI, previous pregnancy loss, number of antenatal care visits, delivery attendant, delivery location, and type of delivery.

9. EFFICACY ANALYSES

9.1 Overview of Efficacy Analysis Methods

The primary efficacy analyses will be performed using the mITT population. All other efficacy analyses will be performed either using the mITT, ITT or per-protocol population as detailed below. The data will be summarized by treatment arm, overall and by country. All model-based analyses and test-statistics examining the treatment effect will adjust for GN site as a randomization variable. Additional details are presented in the sections below.

9.2 Definition of Analysis Variables

The following table defines each of the effectiveness and process analysis variables.

Variable	Type	Definition
**Primary Outcome**		
Preterm birth	Binary	The unit of analysis is the pregnancy delivery with the outcome classified as "Yes" if the delivery is classified as preterm and "No" if not. Preterm is defined as delivery at or after 20 0/7 weeks and prior to 37 0/7 weeks and includes both live births and stillbirths. This definition is consistent with<br>- WHO definition: babies born before 37 completed weeks of pregnancy.<br>- ACOG definition: Birth occurs between 20 weeks and 37 weeks of pregnancy<br>Analysis Population: mITT
**Secondary Outcomes**		

Variable	Type	Definition
Preeclampsia and eclampsia (or more appropriately hypertensive disorders of pregnancy)	Binary	The unit of analysis is the woman randomized to the trial, but the outcome will be defined by the characterization of evidence of hypertensive disorder, including either preeclampsia or eclampsia occurring during the pregnancy. The algorithm for defining this endpoint is included as an addendum to this SAP in HypertensionAlgortihm_V1.1_10052018 (amendments to this algorithm may occur as part of the endpoint adjudication process and will be documented in new versions of this addendum accordingly).<br>• Briefly, any of the following will count as evidence of hypertensive disease:<br>o Any reported SAE of preeclampsia or eclampsia.<br>o MNH registry report of hypertensive disease, preeclampsia or eclampsia.<br>o Reports of elevated blood pressures that meet criteria based on the ACOG 2013 "Hypertension in Pregnancy" Task Force Report at any point after 20 weeks GA.<br>▪ The only case that will programmatically qualify for evidence of hypertension is: Any individual with **2 consecutive timepoints** with ≥ 140 mm Hg systolic **or** ≥ 90 mm Hg diastolic where those timepoints occur more than a week (7 days) apart. The criteria (e.g. elevated systolic or elevated diastolic) must be consistent for the two consecutive visits.<br>• For individuals that do meet the above criteria<br>o If they have less no reports of elevated blood pressure or a single report of elevated blood pressure that is followed by a normal value, they will not be considered to have evidence of hypertensive disease.<br>o If they have any other reports of elevated blood pressure, their outcome classification will be adjudicated in a masked manner by clinical experts.<br><br>Will excluded any MTP but include withdrawals and terminations as data are available.<br>Analysis Population: mITT<br><br>.

**Variable**	**Type**	**Definition**
Small for Gestational Age	Binary	The unit of analysis is the infant delivered from each individual pregnancy. The outcome will be classified as "Yes" if the infant is classified as SGA, defined as a newborn with weight below the 10th percentile for gestational age" and "No" otherwise. Note that growth curves appropriate for each GN site based on Intergrowth standards will be used to define the expected gestational age distribution.<br>For any outcomes involving birth weight, the reported weight will only be used to calculate that outcome if collected within 4 days of birth. Any weight captured after 4 days will be excluded.<br>Analysis Population: mITT
Perinatal Mortality	Binary	The unit of analysis is the infant delivered from each individual pregnancy. The outcome will be classified as "Yes" if the infant is a fresh or macerated stillbirth or a death in the first week of life; the perinatal period commences at 20 completed weeks (154 days) of gestation and ends seven completed days after birth. Pregnancies terminated prior to 20 completed weeks of gestation will be treated as missing for this outcome.<br>Analysis Population: mITT

Variable	Type	Definition
Other Outcomes of Interest—Maternal Outcomes		
Preterm, preeclampsia	Binary	The unit of analysis is the woman randomized to the trial who delivers after 20 weeks gestational age. The outcome will be defined as delivered at less than 34 weeks GA AND also meeting the criteria for the endpoint “Preeclampsia and eclampsia (or more appropriately hypertensive disorders of pregnancy).”<br>Analysis population: mITT
Vaginal Bleeding	Binary	The unit of analysis will be individual woman. Per the protocol, Vaginal Bleeding is defined as: “Bleeding during pregnancy (i.e., WHO generally defines as ‘light’ or ‘heavy’ bleeding based on clinical symptoms).” This outcome will be defined based on Question 2e of Form ASP06 and will be classified as “Yes” if the response to this question is Yes at any bi-weekly visit, and No if all completed biweekly visits have either a No or a DK response. Any woman with at least one biweekly visit form will be included in the denominator; the outcome will be classified as missing if a subject has no biweekly visit forms.<br>Analysis population: ITT
Antepartum Hemorrhage	Binary	The unit of analysis will be individual woman with a delivery after 22 weeks. Per the protocol, antepartum hemorrhage is defined as:” Bleeding from the genital tract at any time after the 22nd week of pregnancy and before the birth of the baby. There are two main causes of antepartum hemorrhage, placenta previa and abruption placentae.” This outcome will be defined based on Question A8b from Form MN02 from the MNH Registry. The event is defined as follows in the MNH Registry Manual of Operations: “Defined as blood loss of > 1000 cc of blood prior to delivery.”<br>Analysis population: mITT

Variable	Type	Definition
Postpartum Hemorrhage	Binary	The unit of analysis will be individual woman. Per the protocol, postpartum hemorrhage is defined as:" Blood loss of 1000 ml or more from the genital tract after delivery and up to six weeks post-delivery. Common causes include atony (poor muscle tone) of the uterus or trauma to the genital tract, e.g. tears of the vagina, cervix, or lower segment of the uterus." This outcome will be defined based on Question A8c from Form MN02 or Question B7 from Form MN03 from the MNH Registry. The event is defined as follows in the MNH Registry Manual of Operations: Defined as blood loss of > 1000 cc of blood after delivery prior to six-week visit.<br>Analysis population: Safety
Maternal mortality through 42 days	Binary	The unit of analysis will be women randomized. Per the protocol, maternal mortality is defined as: "the death of a woman during pregnancy (i.e. conception to delivery) and the puerperium (i.e. up to 42 days after delivery)." The event will be defined as "Yes" if the woman dies prior to 42 days after delivery and "No" otherwise. This outcome measure will be obtained from forms MN02 and MN03 in the MNH Registry. If the 42-day outcome is missing and the woman was alive at the last known follow-up, this outcome will be treated as missing.<br>Analysis population: ITT
Late Abortion	Binary	The unit of analysis will be individual woman. Per the protocol, late abortion is defined as: "Spontaneous fetal loss ≥ 16 weeks." This outcome will be defined based on a response of 4 for Question A7 from Form MN02 from the MNH registry and will be classified as "Yes" if that response is listed as the Mode of Delivery and the pregnancy has a GA of 16 weeks or greater and "No" otherwise. Per the MNH Registry Manual of Operations, that mode of delivery should be used if "A delivery prior to 20 weeks gestation (or <500 g). Miscarriage is also known as spontaneous abortion." If the delivery is after 20 weeks and is classified as a miscarriage on the form, the outcome will be reclassified as a stillbirth. Individuals with loss prior to 16 weeks will be excluded from the analyses.<br>Analysis population: ITT

Variable	Type	Definition
Other Outcomes of Interest—Fetal Outcomes		
Preterm birth <34 0/7 weeks of pregnancy	Binary	The unit of analysis will be the individual delivered fetus or infant (either live birth or stillbirth); note that per the protocol, the event is defined as: "live birth before 34 0/7 weeks of pregnancy are completed," so this definition represents a departure from the protocol. This will be defined as a secondary variable in the analysis data set based on gestational age as determined from ASP forms and neonatal outcome as determined from Form MN02 from the MNH Registry.<br>Analysis population: mITT
Birth weight < 2500g	Binary	The unit of analysis will be the individual infant and per the protocol, the event is defined as: "Birth weight is defined as the first weight of the fetus or newborn obtained after birth. For live births, birth weight should preferably be measured within the first hour of life before significant postnatal weight loss has occurred, recorded to the degree of accuracy to which it is measured (WHO). Birth weight <2500 g is defined as low birth weight" This outcome measure will be a derived measure obtained using the birth weight information provided on Questions C3-C5 from Form MN02 from the MNH Registry. If the actual measure is obtained, the binary variable will be generated from Question C3; otherwise it will be based on the response to Question C5.<br>For any outcomes involving birth weight, the reported weight will only be used to calculate that outcome if collected within 4 days of birth. Any weight captured after 4 days will be excluded.<br>Analysis population: mITT

Variable	Type	Definition
Birth weight < 1500g	Binary	The unit of analysis will be the individual infant and per the protocol, the event is defined as: "Birth weight is defined as the first weight of the fetus or newborn obtained after birth. For live births, birth weight should preferably be measured within the first hour of life before significant postnatal weight loss has occurred, recorded to the degree of accuracy to which it is measured (WHO). Birth weight <1500 g is defined as very low birth weight." This outcome measure will be a derived measure obtained using the birth weight information provided on Questions C3-C5 from Form MN02 from the MNH Registry. If the actual measure is obtained, the binary variable will be generated from Question C3; otherwise it will be based on the response to Question C5.<br>For any outcomes involving birth weight, the reported weight will only be used to calculate that outcome if collected within 4 days of birth. Any weight captured after 4 days will be excluded.<br>Analysis population: mITT
Fetal Loss	Binary	The unit of analysis will be the individual delivered fetus or infant and per the protocol, the event of fetal loss is defined as: "Spontaneous loss ≥ 16 weeks GA plus perinatal mortality." This binary outcome measure will be defined in the analysis data set based on gestational age as measured via the ASP forms and mortality outcomes as determined from Forms MN02 and MN03 from the MNH Registry. Any death of an infant greater than 16 weeks GA and prior to 7 days post-delivery will be classified as "Yes" and live births classified as alive at 7 days will be classified as "No." Any fetal loss (including MTP or miscarriage) that occurs prior to 16 weeks will be treated as a missing value.<br>Analysis population: ITT

**Variable**	**Type**	**Definition**
Spontaneous abortion	Binary	The unit of analysis is the individual pregnancy. Per the protocol, the binary outcome of spontaneous abortion is defined as: "Premature expulsion of a non-viable fetus from the uterus at < 20 weeks gestation." This outcome will be defined based on Questions A7-4 or C1 from Form MN02 from the MNH registry and will be classified as "Yes" if the response to A7-4 is listed as the Mode of Delivery or the response to C1. Fetal/Neonatal outcome is answered 1. Miscarriage and "No" otherwise. Per the MNH Registry Manual of Operations, that mode of delivery should be used if "A delivery prior to 20 weeks gestation (or <500 g). Miscarriage is also known as spontaneous abortion."<br>Analysis population: ITT
Stillbirth	Binary	The unit of analysis will be the individual infant that delivers at 20 weeks gestational age or greater during the specified analysis period. The event will be classified as "Yes" if the infant is classified as a non-macerated still birth or with maceration status unknown via Forms MN02 and MN05 in the MNH Registry and "No" if the baby is born alive as specified via Form MN02 in the MNH Registry. Note that infants classified as Macerated still births are not included in this population. Also note that deliveries classified as miscarriages but that have GA of 20 weeks or greater will be reclassified as stillbirths.<br>Analysis population: mITT
Medical Termination of Pregnancy (MTP)	Binary	The unit of analysis is the individual pregnancy. Per the protocol, the binary outcome of MTP is defined as: "an operation or other procedure to terminate pregnancy before the fetus is viable." This outcome will be defined based on Question A7-5 or Question from Form MN02 from the MNH registry and will be classified as "Yes" if the response 5 is listed as the Mode of Delivery on Question A7 or if Question C1. Fetal/neonatal outcome is answered 2. MTP and "No" otherwise. Per the MNH Registry Manual of Operations, that mode of delivery should be used if "This response includes any medical termination of pregnancy, including abortion (therapeutic abortion, medical abortion, etc.)."<br>Analysis population: ITT

9.4 Primary Analysis

The primary analysis will compare the risk of preterm birth between the two treatment arms using two complementary approaches. First, the formal test of the primary hypothesis that the risk of preterm birth differs between the two arms will utilize a modified intention-to-treat approach based on a Cochran-Mantel-Haenszel test stratified by Global Network sites. The approach is characterized as a modified intention to treat approach because the analysis population for the test will be all randomized pregnancies for which the delivery occurs after 20 weeks. Earlier deliveries (miscarriages) will be considered missing completely at random for purposes of this analysis.

In order to evaluate the sensitivity of the primary hypothesis test to the assumption by which miscarriages are treated as randomly missing and to control for potential confounders of the treatment effect, additional analyses will be conducted using a series of three generalized linear models. These generalized linear models will be fit with the binary outcome of preterm (<37-week delivery) as the outcome measure. The initial model will include terms for treatment, site, and a treatment by site interaction. If the treatment by site interaction term is found to be significant ($p<0.05$), then all subsequent models will include the interaction term and all effects will also be reported by site, although formal inference will be based on the average effect across sites. If the interaction term is not significant, it will be removed from the model and this initial model be used to estimate an unadjusted treatment effect controlling for site. The second series of models will include any demographic or clinical variable found to differ significantly between the treatment arms in the preliminary analyses described above. These models will be used to generate adjusted estimates of the treatment effect controlling for potential confounders. The third set of models will utilize extensions of the generalized linear model that incorporate inverse probability weighting to evaluate the sensitivity of the inference to treatment of miscarriages as randomly missing (Hogan, 2004). As described in Section 7.3, we will also conduct sensitivity analyses using the full ITT population; for these analyses, missing data due to loss to follow-up will be handled using multiple imputation, and these early terminations will be treated as preterm deliveries.

9.5 Secondary Outcome Analyses

This study has been designed to evaluate the differences between the two treatment arms for three secondary outcomes: the risk of perinatal mortality; the risk of eclampsia/preeclampsia, and the risk of a SGA delivery. As with the primary analyses, each of these outcomes will be examined individually using descriptive tests of hypotheses that the risk differs between the two arms based on Cochran-Mantel-Haenszel tests stratified by Global Network sites. The analyses for each of these 3 secondary outcomes will utilize the same modified intention-to-treat population used for the primary outcome. Each of these hypothesis tests will be conducted at the 0.05 level of significance with no adjustment for multiple comparisons. In addition to this set of hypothesis tests, model-based analyses comparable to those conducted for the primary analyses will be used to evaluate potential heterogeneity of treatment effect across sites and evaluate potential confounding. For each of the 3 outcomes, an initial model will include terms for treatment, site, and a treatment by site interaction. If the treatment by site interaction term is found to be significant ($p<0.05$), then all subsequent models will include the interaction term and all effects will be reported by site; because the study will lack power to detect site-specific effects, all p-values will be reported for the average effect and site effects will include point and interval estimates. If the interaction term is not significant, it will be removed from the model and this initial model be used to estimate an unadjusted treatment effect controlling for site. The second series of models will include any demographic or clinical variable found to differ significantly between the treatment arms in the preliminary analyses described above. These models

will be used to generate adjusted estimates of the treatment effect controlling for potential confounders.

9.6 Analyses of Other Outcomes of Interest

In addition to these planned formal primary and secondary outcome analyses, we will also conduct exploratory outcomes for a number of other binary outcomes. The analytic approach for these exploratory analyses, will be comparable to that planned for the primary and secondary analyses but will focus on estimation of effect sizes and generation of hypotheses, therefore the analytic approach will be comparable to the effect estimation approach described for the primary analysis. Initially, contingency tables will be used to generate estimates of the risk of each outcome for the two treatment arms, both overall and separately by Global Network (GN) site and across all sites, and the relative risk associated with treatment will be estimated by site and across sites controlling for site. For each outcome a series of generalized linear models analogous to those described for the primary outcome will be used to generate unadjusted estimates of risk, estimates of risk adjusted for potential confounders, and estimates of risk adjusted to account for any possible differences in risk between the arms associated with miscarriages.

10. SAFETY ANALYSIS:

No formal safety analyses are planned as a part of this study, although rates of pregnancy complications identified by ultrasound will be summarized as will SAEs and hospitalizations. No formal hypothesis tests will be conducted, but descriptive p-values generated using Cochran-Mantel-Haenszel statistics with stratification by GN site will be generated.

11. INTERIM ANALYSES AND DATA MONITORING

No formal interim efficacy analyses were planned nor conducted for the study. Because the study was planned with an enrollment period of 24 months with the duration between randomization and determination of study endpoint being 6 to 8 months, preliminary analyses indicated that all participants would have completed randomization well before sufficient data could be accumulated for an interim efficacy analysis.

While no formal efficacy analysis was planned, the Data Monitoring Committee (DMC) reviewed the study at periodic intervals, to evaluate whether any safety or study progress issues warrant possible study stopping. These periodic reviews were based on DMC reports prepared by the Data coordinating center, which included information on study enrollment rates and participant progress through the study, participant compliance with protocol-specified treatment regimens, protocol violations, adverse events, and efficacy outcomes. The focus of the DMC review was on monitoring participant safety and study progress/futility but data on treatment effectiveness was also presented to frame the DMC discussions on safety and futility.

The primary focus of the DMC was on the safety of study participants and the DMC monitored adverse events and side effects from LDA. All known associated side effects and specific obstetric or fetal concerns were considered reportable to the DMC.

The DMC also reviewed the study progress for study progress and futility, with futility for these analyses defined in terms of inability to achieve study enrollment goals. To effectively complete the study within the parameters defined by NICHD and the Global Network Steering Committee, the goal

of study was to complete enrollment in a 24-month period, and enrollment be completed within 30 months. Consequently, cumulative enrollment for the study should at worst case achieve a minimum of 20%, 40%, 60%, and 80% of total enrollment at the 6, 12, 18, and 24-month DMC reviews starting from the time the study is first opened to enrollment. Should the study have failed to achieve at least 90% of the cumulative target at any review, the DMC would have requested from the protocol committee an action plan for increasing enrollment that should be delivered to the DMC for review within a 1-month period. Should the DMC have considered the plan inadequate or should the plan not result in increased enrollment by the next review, the DMC would have considered recommending to the NICHD closing the study for futility.

12. CHANGES TO THE ANALYSES PLANNED IN THE PROTOCOL

Additional sensitivity analyses beyond those included in the original protocol have been included in Sections 7.3 and 9 to account for loss-to-follow-up and to account for early pregnancy losses. Also, one secondary outcome variable has been modified and on secondary outcome variable has been added. The outcome of risk of preeclampsia or eclampsia has been modified to clarify that this outcome is the risk of a hypertensive disorder of pregnancy. This modification was made to clarify the true variable of interest, reflecting the data collection process used in the MNH registry. The outcome of preterm, preeclampsia has been added to the secondary outcome list to reflect additional research that was reported on this outcome after the study was launched.

13. REFERENCES

.

Abramovici A, Cantu J, Jenkins SM. Tocolytic Therapy for Acute Preterm Labor. Obstet Gynecol Clin North Am. 2012; 39:77-87. doi: 10.1016/j.ogc.2011.12.003.

Anderson RN, Smith BL. Deaths: leading causes for 2001. Natl Vital Stat Rep. 2003;52:1-85.

Beck S, Wojdyla D, Say L, et al. The worldwide incidence of preterm birth: A systematic review of maternal mortality and morbidity. Bull World Health Organ. 2010;88:31-38.

Blencowe H, Cousens S, Oestergaard MZ, et al. National, regional, and worldwide estimates of preterm birth rates in the year 2010 with time trends since 1990 for selected countries: a systematic analysis and implications. Lancet. 2012;379:2162-2172. doi:10.1016/S0140-6736(12)60820-4.

Goldenberg R, Rouse D. Prevention of Premature Birth. N Engl J Med. 1998;339(5):313-320.

Hogan JW, Lancaster T. Instrumental variables and inverse probability weighting for causal inference from longitudinal observational studies. Stat Methods Med Res. 2004;13:17-48. doi:10.1191/0962280204sm351ra.

Liang KY and Zeger SL. Longitudinal Data Analysis Using Generalized Linear Models. Biometrika. 1986:73(1): 13-22.

March of Dimes, PMNCH, Save the Children, WHO. Born Too Soon: The Global Action Report on Preterm Birth. Geneva: World Health Organization; 2012.

Mathews TJ, Menacker F, MacDorman MF. Infant mortality statistics from the 2002 period: linked birth/infant death data set. Natl Vital Stat Rep. 2004;53:1-29

Maine D, Rosenfield A, McCarthy J, Kamara A, Lucas AO: Safe Motherhood Programs: options and issues. New-York: Columbia University; 1991.

McCormick MC, Richardson DK. Premature infants grow up. N Engl J Med. 2002;346(3):197-198. doi:10.1056/NEJM200201173460310.

McClure EM, Pasha O, Goudar SS, et al; Global Network Investigators. Epidemiology of stillbirth in low-middle income countries: a Global Network Study. Acta Obstet Gynecol Scand. 2011;90(12):1379-85.

Oestergaard MZ, Inoue M, Yoshida S, Mahanani WR, Gore FM, Cousens S, Lawn JE, Mathers CD; United Nations Inter-Agency Group for Child Mortality Estimation and the Child Health Epidemiology Reference Group. Neonatal mortality levels for 193 countries in 2009 with trends since 1990: a systematic analysis of progress, projections, and priorities. PLoS Med. 2011;8(8):e1001080.

Schisterman EF, Silver RM, Lesher LL, et al. Preconception low-dose aspirin and pregnancy outcomes: Results from the EAGeR randomised trial. Lancet. 2014;384:29-36. doi:10.1016/S0140-6736(14)60157-4.

Saigal S, Doyle LW. An overview of mortality and sequelae of preterm birth from infancy to adulthood. Lancet. 2008;371:261-269. doi:10.1016/S0140-6736(08)60136-1.

Valero De Bernabé J, Soriano T, Albaladejo R, et al. Risk factors for low birth weight: A review. Eur J Obstet Gynecol Reprod Biol. 2004;116:3-15. doi:10.1016/j.ejogrb.2004.03.007.

14. ATTACHMENTS

Table 1	Participant Distribution
Table 2	Baseline Characteristics
Table 3	Patient Medication Compliance
Table 4	Reasons for Noncompliance
Table 5	Primary and Secondary Efficacy Outcomes
Table 6	Key Safety (SAE) Outcomes
Table 7	Protocol Deviations
Figure 1	Consort Diagram

Table 1. Participant Distribution

GN Site	Screened N	Consented N (%)	Eligible N (%)	Randomized N (%)	Analysis Population Membership N (%)				Withdrew consent N (%)	Study Terminations - N (%)		
					Safety	MITT	ITT	PP		Terminated	Participant moved away from study area	Investigator terminated participant
GN02: DRC												
GN03: Zambia												
GN06: Guatemala												
GN08: India (Belagavi)												
GN09: Pakistan												
GN11: India (Nagpur)												
GN12: Kenya												
Total												

NOTE: Analysis population membership % will be out of randomized.

Table 2: Baseline Characteristics – For ITT and MITT Populations

Variable	Placebo	Aspirin	Total
Randomized, N			
Maternal Age, N (%)			
< 20			
20-29			
> 29			
Median (P25, P75)			
Projected GA at Enrollment, N (%)			
6,0 - 7,6			
8,0 - 9,6			
10,0 - 10,6			
11,0-11,6			
12,0 - 13,6			
Median (P25, P75)			
Maternal Education, N (%)			
No formal			
Primary			
Secondary			
University+			
Maternal Height			
Mean (StdDev)			
Median (Min, Max)			
Maternal Weight			
Mean (StdDev)			
Median (Min, Max)			
BMI			

Variable	Placebo	Aspirin	Total
Mean (StdDev)			
Median (Min, Max)			
Number of Antenatal Care Visits			
Median (P25, P75)			
Delivery Attendant, N (%)			
Physician			
Nurse/Nurse midwife			
TBA			
Family/Self/Other			
Delivery Location, N (%)			
Hospital			
Clinic/Health Center			
Home/Other			
Delivery Mode, N (%)			
Vaginal			
C-Section			
Miscarriage			
MTP			

Table 3: Participant Medication Exposure and Compliance – For MITT and ITT Populations

Compliance/Exposure Parameter	Placebo (N = XXXX)	Aspirin (N = XXXX)
No Drug		
Gestational age at first dose (weeks)		
Mean (StdDev)		
Median (Min, Max)		
Gestational age at last dose (weeks)		
Mean (Std Err)		
Min, Max		
Total number of days doses taken		
Mean (Std Err)		
Min, Max		
Level of Adherence: N (%)		
0% – 30%		
30% – 50%		
50% – 70%		
70% – 90%		
>90%		
Level of Exposure: N (%)		
0% – 30%		
30% – 50%		
50% – 70%		
70% – 90%		
>90%		

NOTE: Summaries of gestational age at first and last dose excludes individuals who never initiated treatment while such individuals are included in summaries of all other parameters.

Table 3a: Medication Compliance by Site – MITT Population

		Level of compliance					
GN Site	Enrolled N	Total Dosed N	< 30% N (%)	30-50% N (%)	50-70% N (%)	70-90% N (%)	> 90% N (%)
GN02: DRC							
GN03: Zambia							
GN06: Guatemala							
GN08: India (Belagavi)							
GN09: Pakistan							
GN11: India (Nagpur)							
GN12: Kenya							

Table 4: Reasons for Medication Noncompliance – MITT Population

Measures of Non-Compliance	Placebo	Aspirin
Number of Biweekly Visits N		
Visits where pills not taken as instructed, N (%)		
Reasons for Noncompliance, N (%)		
Another person took pills		
Pills were lost		
Pills made me feel sick		
Forgot to take pills		
Travelled and did not bring pills		
Didn't understand instructions for pills		
Pills were destroyed		
Other		

Table 5a: Primary and Secondary Efficacy Outcomes

	Placebo N = XXXX	Aspirin N = XXXX	p-value	Unadjusted RR (95% CI)	Adjusted RR (95% CI)
**Primary Outcome, N included**					
Preterm Delivery, N (%)					
**Secondary Efficacy Outcomes**					
Hypertensive Disorders, N (%)					
Small for Gestational Age, N (%)					
Perinatal Mortality, N (%)					
**Other Maternal Outcomes of Interest**					
Vaginal bleeding, N (%)					
Antepartum hemorrhage, N (%)					
Postpartum Hemorrhage, N (%)					
Maternal mortality through 42 days, N (%)					
Late Abortion, N (%)					
Preterm Preeclampsia, N (%)					
**Other Fetal Outcomes of Interest**					
Preterm birth <34 0/7 weeks of pregnancy, N (%)					
Birth weight < 2500g, N (%)					
Birth weight < 1500g, N (%)					
Fetal Loss, N (%)					
Spontaneous abortion, N (%)					
Stillbirth, N (%)					
Medical Termination of Pregnancy (MTP), N (%)					

NOTE: Table contains outcomes summarize using MITT population as well as outcomes summarized using ITT population. Final table will clearly identify which population is used for each outcome. Birth weight will be summarized both including and excluding estimated weights. Adjusted RR will not be presented in primary paper. Footnotes will be included to describe p-value is from CMH test as well as models used to obtain RR estimates.

Table 5b: Primary and Secondary Efficacy Outcomes by Site

NOTE: Table will also include fetal outcomes of interest.

Outcome Measure	Risk of Outcome			
	Placebo N = XXXX	Aspirin N = XXXX	RR (95% CI)	p-Value
**Preterm Delivery, N (%)**				
GN01: DRC				
GN03: Zambia				
GN12: Kenya				
GN06: Guatemala				
GN08: Belagavi				
GN11: Nagpur				
GN09: Pakistan				
**Hypertensive Disorders, N (%)**				
GN01: DRC				
GN03: Zambia				
GN12: Kenya				
GN06: Guatemala				
GN08: Belagavi				
GN11: Nagpur				
GN09: Pakistan				
**Small for Gestational Age**				
GN01: DRC				
GN03: Zambia				
GN12: Kenya				
GN06: Guatemala				
GN08: Belagavi				
GN11: Nagpur				

Outcome Measure	Risk of Outcome			
	Placebo N = XXXX	Aspirin N = XXXX	RR (95% CI)	p-Value
GN09: Pakistan				
**Perinatal Mortality**				
GN01: DRC				
GN03: Zambia				
GN12: Kenya				
GN06: Guatemala				
GN08: Belagavi				
GN11: Nagpur				
GN09: Pakistan				
**Preeclampsia**				
GN01: DRC				
GN03: Zambia				
GN12: Kenya				
GN06: Guatemala				
GN08: Belagavi				
GN11: Nagpur				
GN09: Pakistan				
**Eclampsia**				
GN01: DRC				
GN03: Zambia				
GN12: Kenya				
GN06: Guatemala				
GN08: Belagavi				
GN11: Nagpur				
GN09: Pakistan				

NOTE: Table contains outcomes summarize using MITT population as well as outcomes summarized using ITT population. Final table will clearly identify which population is used for each outcome. Birth weight will be summarized both including and excluding estimated weights. Footnotes will be included to describe p-value and RR is from unadjusted model that includes a site by treatment group interaction term. The p-value for the interaction term will also be presented.

Table 6: Serious Adverse Event Summary

Variable	Placebo N (%)	Aspirin N (%)	Relative Risk RR (95% CI)	p-Value
Randomized, N				
Participants with at least one SAE[1]				
Maternal death[1], N (%)				
Fetal loss after 20 weeks[1], N (%)				
Fetal anomaly[1], N (%)				
Antepartum hemorrhage[1], N (%)				
Post-partum hemorrhage[1], N (%)				
Preeclampsia/Eclampsia[1], N (%)				
Upper GI bleeding[1], N (%)				
Gastroschisis, N (%)				
At least one Other SAE[1], N (%)				

The denominator for this table is any woman included in the safety population.

Table 7a: Summary of Protocol Deviations by Treatment Arm

Variable	Arm 1	Arm 2	Total
Randomized, N (%)	XXXX	XXXX	XXXX
At least one protocol deviation, N (%)	XXX (Y.Y)	XXX (Y.Y)	XXX (Y.Y)
Protocol deviations, N	XX	XX	XX
Study participant enrolled but did not meet inclusion criteria	XX	XX	XX
Study participant enrolled but met criteria requiring exclusion	XX	XX	XX
Study participant enrolled twice	XX	XX	XX
Medication card was lost or destroyed	XX	XX	XX
The study participant was incorrectly randomized	XX	XX	XX
The randomization assignment was revealed to study personnel, participant or both	XX	XX	XX
Other deviation	XX	XX	XX
Protocol Deviation Rate (N/Person Month), Mean (StdDev)	X.X	X.X	X.X

Table 7b: Summary of Protocol Deviations by Site

Variable	GN02	GN03	GN06	GN08	GN09	GN11	GN12
Randomized, N (%)	XXXX	XXXX	XXXX	XXXX	XXXX	XXXX	XXXX
At least one protocol deviation, N (%)	XXX (Y.Y)	XXX (Y.Y)	XXX (Y.Y)	XXX (Y.Y)	XXX (Y.Y)	XXX (Y.Y)	XXX (Y.Y)
Protocol deviations, N	XX	XX	XX	XX	XX	XX	XX
Study participant enrolled but did not meet inclusion criteria	XX	XX	XX	XX	XX	XX	XX
Study participant enrolled but met criteria requiring exclusion	XX	XX	XX	XX	XX	XX	XX
Study participant enrolled twice	XX	XX	XX	XX	XX	XX	XX
Medication card was lost or destroyed	XX	XX	XX	XX	XX	XX	XX
The study participant was incorrectly randomized	XX	XX	XX	XX	XX	XX	XX
The randomization assignment was revealed to study personnel, participant or both	XX	XX	XX	XX	XX	XX	XX
Other deviation	XX	XX	XX	XX	XX	XX	XX
Protocol Deviation Rate (N/Person Month), Mean (StdDev)	X.X	X.X	X.X	X.X	X.X	X.X	X.X

Figure 1: Consort Diagram

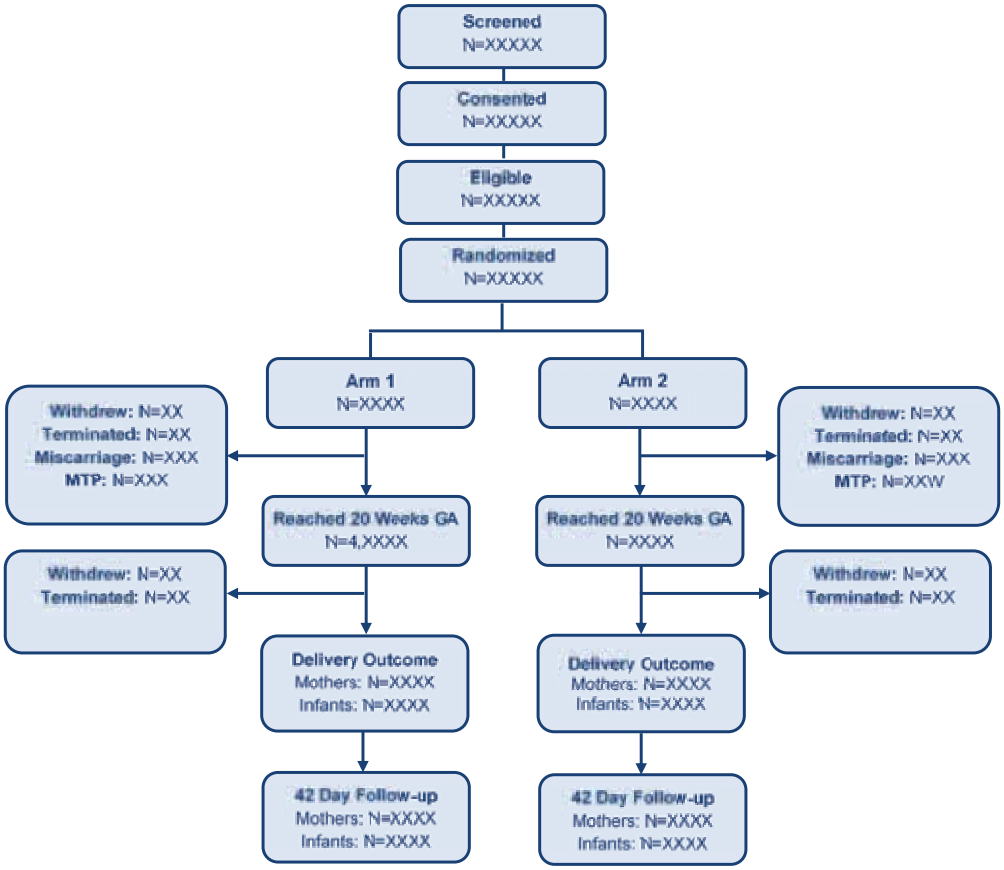

Supplement: Supplement 1. — Trial Protocol and Statistical Analysis Plan [file jamanetwopen-e2611402-s001.pdf]
