# Supplementary material for: Aspirin and Preterm Birth Among Pregnant People With Increased Heat Exposure: Secondary Analysis of a Randomized Clinical Trial
Source: JAMA Netw Open. 2026 May 6;9(5):e2611402. doi: 10.1001/jamanetworkopen.2026.11402 (PMC13150641; doi:10.1001/jamanetworkopen.2026.11402)
Supplement: Supplement 2. — eTable 1. Mean daily maximum, daily minimum, and daily mean wet bulb globe temperatures (in degrees Celsius) by site of the Global Network for Women’s and Children’s Health Research low-dose ASPIRIN trial, during the study period Table 2. Time-varying associations between exposure to averaged maximum wet bulb globe temperature exceeding the site-specific 75th percentile at specific weeks before birth and risk of preterm birth (adjusted odds ratios and 95% confidence intervals) [file jamanetwopen-e2611402-s002.pdf]

Supplemental Online Content

Meltzer GY, Duttweiler LP, Saleem S, et al. Aspirin and preterm birth among pregnant people with increased heat exposure. *JAMA Netw Open*. 2026;9(5):e2611402. doi:10.1001/jamanetworkopen.2026.11402

eTable 1. Mean daily maximum, daily minimum, and daily mean wet bulb globe temperatures (in degrees Celsius) by site of the Global Network for Women's and Children's Health Research low-dose ASPIRIN trial, during the study period

eTable 2. Time-varying associations between exposure to average maximum wet bulb globe temperature exceeding the site-specific 75th percentile at specific weeks before birth and risk of preterm birth (adjusted odds ratios[a] and 95% confidence intervals)

This supplemental material has been provided by the authors to give readers additional information about their work.

eTable 1. Mean daily maximum, daily minimum, and daily mean wet bulb globe temperatures (in degrees Celsius) by site of the Global Network for Women's and Children's Health Research low-dose ASPIRIN trial, during the study period

Site	Maximum WBGT	Minimum WBGT	Mean WBGT	Maximum Heat Index	Minimum Heat Index	Average Heat Index	Maximum Air Temperature	Minimum Air Temperature	Average Air Temperature
Democratic Republic of Congo	26.4	18.0	21.6	35.1	22.8	27.8	31.4	22.1	26.1
Zambia	21.6	12.5	16.9	27.6	16.9	21.9	27.5	17.1	22.0
Kenya	21.7	13.1	17.2	27.6	17.4	22.0	27.6	17.4	21.9
Nagpur, India	26.1	17.0	21.5	34.9	22.1	28.1	33.2	21.9	27.1
Belagavi, India	24.8	16.5	20.6	32.3	21.2	26.4	31.9	21.0	26.0
Guatemala	18.4	9.2	13.3	23.4	13.5	17.7	23.6	13.6	17.9
Pakistan	25.8	17.4	21.7	34.6	22.9	28.6	32.5	21.6	26.5

WBGT = wet bulb globe temperature.

eTable 2. Time-varying associations between exposure to average maximum wet bulb globe temperature exceeding the site-specific 75th percentile at specific weeks before birth and risk of preterm birth (adjusted odds ratios[a] and 95% confidence intervals)

Weeks before birth	Entire Cohort	Aspirin Group	Placebo Group
19	**1.10 (1.02-1.18)**	1.06 (0.95-1.18)	**1.14 (1.03-1.25)**
18	**1.07 (1.02-1.12)**	1.04 (0.97-1.12)	**1.10 (1.03-1.17)**
17	**1.04 (1.01-1.08)**	1.03 (0.98-1.07)	**1.06 (1.02-1.11)**
16	**1.02 (1.00-1.04)**	1.01 (0.98-1.05)	1.03 (0.99-1.06)
15	1.00 (0.97-1.03)	1.00 (0.96-1.04)	1.00 (0.96-1.04)
14	0.98 (0.95-1.02)	0.99 (0.94-1.04)	0.98 (0.94-1.03)
13	0.98 (0.94-1.01)	0.98 (0.93-1.04)	0.97 (0.92-1.02)
12	0.97 (0.94-1.01)	0.98 (0.93-1.03)	0.97 (0.92-1.02)
11	0.97 (0.95-1.00)	0.98 (0.94-1.02)	0.97 (0.93-1.01)
10	0.98 (0.96-1.00)	0.98 (0.95-1.01)	0.98 (0.95-1.02)
9	0.99 (0.97-1.01)	0.98 (0.95-1.02)	1.00 (0.97-1.03)
8	1.00 (0.97-1.03)	0.99 (0.94-1.03)	1.01 (0.97-1.06)
7	1.01 (0.97-1.05)	0.99 (0.94-1.05)	1.03 (0.97-1.08)
6	1.01 (0.97-1.06)	0.99 (0.94-1.06)	1.03 (0.98-1.09)
5	1.02 (0.98-1.05)	1.00 (0.95-1.05)	1.03 (0.98-1.09)
4	1.02 (0.99-1.05)	1.00 (0.96-1.05)	1.03 (0.99-1.07)
3	1.01 (0.99-1.04)	1.01 (0.97-1.04)	1.02 (0.98-1.06)
2	1.01 (0.97-1.04)	1.01 (0.96-1.06)	1.01 (0.96-1.06)
1	1.00 (0.95-1.06)	1.01 (0.94-1.09)	0.99 (0.92-1.07)

[a] Pooled mixed-effects logistic distributed lag model adjusted for maternal age, infant sex, and gestational age, with random effects for site. Bolded weeks are statistically significant
