# Supplementary material for: Aspirin and Preterm Birth Among Pregnant People With Increased Heat Exposure: Secondary Analysis of a Randomized Clinical Trial
Source: JAMA Netw Open. 2026 May 6;9(5):e2611402. doi: 10.1001/jamanetworkopen.2026.11402 (PMC13150641; doi:10.1001/jamanetworkopen.2026.11402)
Supplement: Supplement 3. — Data Sharing Statement [file jamanetwopen-e2611402-s003.pdf]

Data Sharing Statement

Meltzer. Aspirin and Preterm Birth Among Pregnant People With Increased Heat Exposure. *JAMA Netw Open*. Published May 06, 2026. doi:10.1001/jamanetworkopen.2026.11402

Data
Additional Information: Aspirin Supplementation for Pregnancy Indicated Risk Reduction In Nulliparas (ASPIRIN) https://clinicaltrials.gov/study/NCT02409680?cond=Preterm%20Birth&intr=Aspirin&aggFilters=status:com&rank=2 ClinicalTrials.gov ID: NCT02409680
Data available: Yes
Data types: Deidentified participant data, Data dictionary
How to access data: https://dash.nichd.nih.gov
When available: With publication

Supporting Documents
Document types: Statistical/analytic code, Other (please specify)
Additional Information: Trial Protocol
How to access documents: mcclure@rti.org
When available: With publication

Additional Information
Who can access the data: Data will be available upon request.
Types of analyses: For any purpose
Mechanisms of data availability: Through the National Institute of Child Health and Development Data and Specimen Hub
